# Quantitative trait loci for leaf chlorophyll fluorescence parameters, chlorophyll and carotenoid contents in relation to biomass and yield in bread wheat and their chromosome deletion bin assignments

**DOI:** 10.1007/s11032-013-9862-8

**Published:** 2013-04-10

**Authors:** I. Czyczyło-Mysza, M. Tyrka, I. Marcińska, E. Skrzypek, M. Karbarz, M. Dziurka, T. Hura, K. Dziurka, S. A. Quarrie

**Affiliations:** 1 The F. Górski Institute of Plant Physiology, Polish Academy of Sciences, Kraków, Poland; 2 Department of Biochemistry and Biotechnology, Rzeszow University of Technology, Rzeszów, Poland; 3 Institute of Applied Biotechnology and Basic Sciences, University of Rzeszow, Rzeszów, Poland; 4 Faculty of Biology, Belgrade University, Belgrade, Serbia; 5 Newcastle University, Newcastle upon Tyne, UK

**Keywords:** Chlorophyll fluorescence kinetics, Photosynthetic pigment content, Yield and biomass, Quantitative trait loci, Deletion bin, *Triticum aestivum*

## Abstract

Relatively little is known of the genetic control of chlorophyll fluorescence (CF) and pigment traits important in determining efficiency of photosynthesis in wheat and its association with biomass productivity. A doubled haploid population of 94 lines from the wheat cross Chinese Spring × SQ1 was trialled under optimum glasshouse conditions for 4 years to identify quantitative trait loci (QTL) for CF traits including, for the first time in wheat, JIP-test parameters per excited cross section (CS_m_): ABS/CS_m_, DI_o_/CS_m_, TR_o_/CS_m_, RC/CS_m_ and ET_o_/CS_m_, key parameters determining efficiency of the photosynthetic apparatus, as well as chlorophyll and carotenoid contents to establish associations with biomass and grain yield. The existing genetic map was extended to 920 loci by adding Diversity Arrays Technology markers. Markers and selected genes for photosynthetic light reactions, pigment metabolism and biomass accumulation were located to chromosome deletion bins. Across all CF traits and years, 116 QTL for CF were located on all chromosomes except 7B, and 39 QTL were identified for pigments on the majority of chromosomes, excluding 1A, 2A, 4A, 3B, 5B, 1D, 2D, 5D, 6D and 7D. Thirty QTL for plant productivity traits were mapped on chromosomes 3A, 5A, 6A, 7A, 1B, 2B, 4B, 6B, 7B, 3D and 4D. A region on chromosome 6B was identified where 14 QTL for CF parameters coincided with QTL for chlorophyll content and grain weight per ear. Thirty-five QTL regions were coincident with candidate genes. The environment was shown to dominate in determining expression of genes for those traits.

**Electronic supplementary material:**

The online version of this article (doi:10.1007/s11032-013-9862-8) contains supplementary material, which is available to authorized users.

## Introduction

Conventional plant breeding improves plant productivity during cycles of crossing and selection of appropriate genotypes which lead to phenotypic expression of the desired traits, e.g. increase of grain yield. Increasing yield means increasing either the harvest index (the proportion of biomass converted into harvestable grains) or total plant above-ground biomass. Thus, increasing biomass is a major target for breeders, and photosynthetic efficiency is a major physiological determinant of net carbon gain. Therefore, insights into plant productivity can be gained by studying the activity of components of the photosynthetic apparatus, such as chlorophyll fluorescence (CF) kinetics, and assimilation pigment contents (Bolhar-Nordenkampf and Öquist 1993). Such studies provide pivotal information about the photochemical efficiency of photosystem II (PSII) and the amount of excitation energy trapped in PSII reaction centres during photosynthesis. Moreover, such measurements allow estimates to be made of light energy absorption, amount of energy dissipated from PSII, numbers of active reaction centres and amount of energy used for electron transport (Baker and Rosenqvist 2004). Chlorophyll is one of the most important molecules associated with photosynthesis, having important functions in the absorption and exploitation of light energy, thereby influencing photosynthetic efficiency (Pan and Dong 1995). Further elucidation of the molecular mechanisms of CF and their interactions with assimilation pigments will thus have potential significance for yield improvement.

Chlorophyll fluorescence parameters have been used to detect even subtle differences in activity of the photosynthetic apparatus among genotypes of crop plants (Smillie and Nott 1982; Maxwell and Johnson 2000; Pereira et al. 2000; Fracheboud and Leipner 2003; O’Neill et al. 2006). Furthermore, Hura et al. (2009) found significant correlations between the yield of triticale and some parameters of chlorophyll fluorescence and leaf gas exchange.

The routine use now of molecular markers for genetic analysis with mapping populations has enabled the identification of quantitative trait loci (QTL) involved in the expression of important agronomic traits of wheat, such as productivity (e.g. Börner et al. 2002; Campbell et al. 2003; Groos et al. 2003; Quarrie et al. 2005; Kumar et al. 2006, 2007; Huang et al. 2006; Wang et al. 2009; Maccaferri et al. 2011). However, relatively little is known about the genetic control and chromosomal location of genes regulating physiological characteristics, especially components of photosynthesis. Chlorophyll fluorescence and pigment contents have become some of the most powerful and widely-used traits in photosynthesis research available to plant physiologists (Pan and Dong 1995; Maxwell and Johnson 2000; Zhang et al. 2009). Understanding the genetic determinants of chlorophyll fluorescence parameters and chlorophyll contents would help establish a genetic basis for increasing biomass and therefore yield in wheat. Few QTL studies have been reported so far on CF parameters in wheat (Yang et al. 2007a; Liang et al. 2010; Zhang et al. 2010; Czyczyło-Mysza et al. 2011; Kumar et al. 2012; Li et al. 2012) and wheat leaf chlorophyll contents (Cao et al. 2004; Quarrie et al. 2006; Yang et al. 2007a; Zhang et al. 2009, 2010). As far as we are aware, QTL for the JIP-test parameters (Strasser and Strasser 1995) ABS/CS_m_, DI_o_/CS_m_, TR_o_/CS_m_, RC/CS_m_ and ET_o_/CS_m_, based on phenomenological energy fluxes per excited cross section (CS_m_), have not previously been reported for wheat, though QTL associated with some of the JIP-test parameters were recently reported in soybean (Yin et al. 2010).

Establishing the coincidence of QTL for physiologically-related traits provides powerful evidence for causal relationships amongst traits (Prioul et al. 1997). However, for this approach to be effective, the test genome needs to be well covered with markers so that the majority of loci having significant associations with those traits can be identified. The hexaploid wheat genome, with 21 chromosome pairs, is large in comparison with other crop genomes (typically 3,000–4,000 cM, e.g. Marino et al. 1996; Chalmers et al. 2001; Paillard et al. 2003; Quarrie et al. 2005). “Saturating” the hexaploid wheat genome with markers therefore requires many hundreds of markers.

The genetic map presented initially by Quarrie et al. (2005) and extended by Habash et al. (2007) was constructed with a population of 95 doubled haploids from the cross Chinese Spring × SQ1 and had over 500 marker loci with a total map length of 3,522 cM. Nevertheless, it was estimated to cover only 85–90 % of the genome. Therefore, to increase the coverage of the genome, additional markers have been put on the map using Diversity Arrays Technology (DArT). DArT, first reported over 10 years ago for rice (Jaccoud et al. 2001), has become increasingly popular, with over 60 species screened so far, including bread wheat (Akbari et al. 2006; Semagn et al. 2006). This microarray-based technology detects differences in thousands of unique spots representing individual DNA fragments leading to high-throughput, robust, cost- and time-efficient characterization of genotypes with dominant markers. In wheat, DArT markers have been applied to study genetic diversity (Stodart et al. 2007; White et al. 2008), to identify genetic markers linked with economically important traits in association studies (Crossa et al. 2011; Bordes et al. 2011), and for QTL mapping approaches (Prins et al. 2011; Basnet et al. 2012).

The objectives of the work reported here were thus to expand the existing genetic map from Chinese Spring × SQ1 by adding DArT markers, and use it to identify QTL for key measures of photosynthetic activity, namely chlorophyll fluorescence kinetics parameters and chlorophyll and carotenoid contents, and to test their association with biomass productivity and yield in the wheat mapping population trialled over several years. To facilitate the identification of candidate genes for trait QTL, markers on the genetic map together with a number of genes associated with photosynthesis, pigment production and biomass productivity were assigned to the Chinese Spring chromosome deletion bins.

## Materials and methods

### Plant material

The mapping population (CSDH) consisted of 94 doubled haploid (DH) lines generated from the cross between hexaploid wheat (*Triticum aestivum* L.) genotypes Chinese Spring (CS) and SQ1 (a high abscisic acid breeding line) according to Quarrie et al. (2005) and available from the John Innes Centre, Norwich, UK (mike.ambrose@jic.ac.uk). One of the original 95 lines was rejected because of the possibility of seed contamination.

### Experimental design and plant growth

Ninety-four DH lines of the CSDH mapping population and their parental lines CS and SQ1 were studied for 4 years (2007, 2008, 2010 and 2011; experiments E_I_, E_II_, E_III_ and E_IV_, respectively) using spring sowings with vernalization. After vernalization for 7 weeks at 4 °C with an 8/16-h light/dark photoperiod (short day), seedlings were singly planted into soil in 3 L volume pots in three replicates (288 plants each year). Pots were filled with an equal mass of soil and water content and watered regularly to ensure that soil water content was kept optimal. A few spare pots with plants were also grown to determine field water capacity of the soil. Plants were transferred to an open-sided greenhouse (17 May 2007; 19 May 2008; 17 May 2010; 25 May 2011) and grown until harvest in August.

Physiological traits associated with photosynthesis were studied in flag leaves, sampled during early July (2 July 2007—early grain filling; 4 July 2008—around anthesis; 13 July 2010 and 12 July 2011—early grain filling). Chlorophyll contents were assessed *in planta* using a SPAD meter (see below), chlorophyll *a* fluorescence parameters (CF) were measured on detached leaves and chlorophyll and carotenoid contents were assayed in lyophilized leaf tissue. CF parameters were measured in experiments E_I_, E_II_, E_III_ and E_IV_, chlorophyll and carotenoid contents in experiments E_I_, E_II_ and E_III_ and chlorophyll content in SPAD units measured only in experiments E_III_ and E_IV_. Environmental conditions (temperature and humidity) were controlled during all experiments using an AR205 (APAR) apparatus. All four experiments were conducted at the Institute of Plant Physiology of the Polish Academy of Sciences, Kraków, Poland.

### Phenotypic measurements

#### Chlorophyll content measured in SPAD units

SPAD readings were collected on flag leaves of each plant, just before their sampling for other physiological measurements, using a hand-held meter (SPAD 502, Konica-Minolta, Japan). SPAD meter readings have been shown to be linearly related to chlorophyll concentration measured spectrophotometrically over the range of concentrations encountered here (Yadava 1986; Fisher et al. 1998).

#### Chlorophyll *a* fluorescence parameters

Chlorophyll fluorescence was measured on the fully expanded flag leaf using a portable fluorometer (Handy PEA; Hansatech Instruments, King’s Lynn, UK) at ambient temperature after 20 min adaptation of leaves to dark conditions on the day of sampling. Fluorescence intensity was measured with a PIN-photodiode after being passed through a long-pass filter. Changes in fluorescence were registered during irradiation of 10 μs to 1 s. During the initial 2 ms, data were collected every 10 μs with 12-bit resolution. After this period, the frequency of measurements was reduced automatically. The measurements were done on detached leaves (approximately 20 min after detachment) for each line with three plant replicates. The following parameters were calculated per excited leaf cross-section (CS_m_): *F*_v_/*F*_m_ (the maximum photochemical efficiency), PI (overall performance index of PSII photochemistry), ABS/CS_m_ (light energy absorption), TR_o_/CS_m_ (amount of excitation energy trapped in PSII reaction centers), DI_o_/CS_m_ (energy amount dissipated from PSII, equal to [ABS/CS_m_ – TR_o_/CS_m_]), RC/CS_m_ (number of active reaction centres) and ET_o_/CS_m_ (amount of energy used for electron transport). Data were analyzed with a JIP test (Force et al. 2003; Lazár and Pospíšil 1999; Strasser and Tsimilli-Michael 1998; Strasser et al. 2000). After fluorescence measurements, leaves were sampled to determine pigment contents.

#### Chlorophyll and carotenoid contents

After lyophilization in high vacuum at 100 μbar, coil temperature −40 °C (lyophiliser: Freezone 4.5; Labconco, USA), leaf tissue was homogenized using a ball mill (MM200, Retsch, Germany) for 2 min at maximum frequency (30 Hz). Five mg dry weight were then extracted in 1.5 ml of 95 % ethanol for 15 min and centrifuged at 2,000*g* (Universal 32R, Hettich, Germany) for 10 min. An aliquot of the ethanolic extract (100 μl) was added to a 96-well microplate, and absorbances at 470, 648 and 664 nm measured spectrophotometrically with a microplate reader (Synergy II; Bio-Tek, USA). The concentrations of total chlorophyll *a* + *b* (Chl_a+b_) and carotenoids (Car) were calculated according to extinction coefficients given in the following equations (Lichtenthaler and Buschmann 2001), taking account of the path length of microwells filled to a quarter of their depth:

$$ {\text{Chl}}_{\text{a}} \left( {\mu {\text{g}}/{\text{ml}}} \right) = 1 3. 3 6 {\text{A664}} - 5. 1 9 {\text{A648}} $$

$$ {\text{Chl}}_{\text{b}} \left( {\mu {\text{g}}/{\text{ml}}} \right) = 2 7. 4 3 {\text{A648}} - 8. 1 2 {\text{A664}} $$

$$ {\text{Chl}}_{{{\text{a}} + {\text{b}}}} \left( {\mu {\text{g}}/{\text{ml}}} \right) = 5. 2 4 {\text{A664}} + 2 2. 2 4 {\text{A648}} $$

$$ {\text{Car}}\left( {\mu {\text{g}}/{\text{ml}}} \right) = \left( { 1000{\text{A470}} - 2. 1 3 {\text{Chl}}_{\text{a}} - 9 7. 6 4 {\text{Chl}}_{\text{b}} } \right)/ 20 9 $$

where Chl_a_ = chlorophyll *a,* Chl_b_ = chlorophyll *b,* A470 = absorbance at 470 nm, A664 = absorbance at 664 nm, A648 = absorbance at 648 nm.

Concentrations reported are averages of three biological replications, each consisting of two analytical replications, and are expressed as pigment contents per gram dry weight (mg/g DW).

#### Agronomic traits

At final maturity, plants were cut at the soil surface, weighed after drying to obtain above-ground biomass and separated into the main shoot and the rest. For each plant, grain yield of the main stem (GWE), grain yield per plant (YP) and dry weight per plant at harvest (DWP) were measured.

### Genotypic measurements

#### Saturation of CSDH genetic map with DArT markers

Ninety DH lines of the CSDH mapping population and parents CS and SQ1 were analysed. The set did not contain lines 6, 12, 16, 44 and 96 in comparison with the map published earlier (Quarrie et al. 2005; Habash et al. 2007) for 95 DH lines. DNA was isolated according to Milligan’s method (Milligan 1992) and its concentration was determined on a 1 % agarose gel. Samples were prepared according to the instructions and sent for DArT analyses to Triticarte (Australia, http://www.triticarte.com.au/).

The genetic map was constructed with the software JoinMap 4 (van Ooijen 2006). Marker linkage groups were selected at LOD >3.0. Markers within groups were then ordered using the maximum likehood algorithm of the RECORD program (van Os et al. 2005). The proposed sequence of markers was checked graphically and subjected to correction which involved the replacement of single data of double *crossing over* with missing data. The sequence of markers was recalculated and the distances between loci were determined with the Kosambi function (Kosambi 1944).

#### Chromosome bin assignments and mapping of genes for photosynthetic light reactions, pigment metabolism and biomass accumulation

Chromosome bin assignments of markers on the genetic map were made according to published information (Supplementary Table S1). Markers were assigned either according to previous mapping to a chromosome bin, or according to known bin assignments for flanking markers in other genetic maps or because they were located on other wheat genetic maps at the same location as another marker of known bin location. Other markers coincident with a bin-mapped marker were assumed to be located to the same bin.

Chromosome bin locations for several nuclear genes encoding proteins involved in photosynthetic light reactions, chlorophyll and carotenoid metabolism, as well as several other genes involved in biomass (carbohydrate) accumulation, were identified mainly according to chromosome deletion bin assignments for wheat ESTs available from GrainGenes (http://wheat.pw.usda.gov/cgi-bin/westsql/map_locus.cgi). Other genes were located by comparative mapping using flanking markers on other genetic or physical maps. Some assignments were based on copies known or assumed to be present on homoeologous wheat chromosomes. A few genes were mapped as restriction fragment length polymorphism (RFLP) probes directly in the mapping population. Genes are located on the genetic map where an approximate location could be identified; otherwise they are located to the middle of the deletion bin. Methods used to assign chromosomal locations to these genes and their abbreviations are summarized in Supplementary Table S2.

### Statistical analysis and QTL mapping

Standard statistical analyses for standard deviation, skewness and kurtosis were performed using Microsoft Excel 2010. Distributions of traits and equality of variances were analyzed respectively with Shapiro–Wilk and Levene tests using Statistica 9.1 software (StatSoft Inc. 2010). The set of data was subjected to analysis of variance (ANOVA) to determine the specific effects of genotype (i.e. the DH line) and year as factors. Broad-sense heritability (*h* ^2^) estimates were calculated from variance components according to Cherif et al. (2010) and International Rice Research Institute (2006).

QTL for mean data each year were identified using Windows QTLCartographer version 2.5 (Wang et al. 2011). In the first stage of analysis, a linear regression SMA (single marker analysis) method was used to identify a connection between a single marker and phenotype. Composite interval mapping (CIM) was then carried out. Testing the presence of a QTL in a given mapping interval was done by calculating the LOD (logarithm of odds). A QTL locus was identified in a region designated by its maximum LOD score, and declared significant if it exceeded a critical value, determined by 1,000 permutations (typically LOD >3.3). However, as this criterion generated relatively few significant QTL, QTL with maximum LOD scores from 2.0 to 3.3 are also presented. For comparative analyses, a few QTL with LOD maxima of 1.8 and 1.9 were included, and a coincidence of QTL was assumed when CIM LOD score maxima were within a 10-cM interval, representing a minimum precision typical for QTL detection (Mangin and Goffinet 1997).

## Results

### Environments and phenotypes

#### Environmental conditions

Daily mean temperatures and air humidities (Supplementary Fig. S1) were similar in 2007 (E_I_), 2010 (E_III_) and 2011 (E_IV_), though most of May–July, 2008 (E_II_) was 5–10 °C warmer, with much lower humidities. The highest average temperature in 2007, 2010 and 2011 occurred in the second decade of July: 29, 25 and 22 °C, respectively. In contrast, the second decade of July had the lowest temperature in 2008.

#### Phenotypic variation

Analysis of variance showed highly significant differences between DH lines and between years and interactions for all traits (Table 1). The dominant source of variation for each trait was year-to-year. Supplementary Table S3 shows that DH lines with minimum and maximum trait means varied between years, from ratios of 1.04 (*F*_v_/*F*_m_) to 5.59 (YP). This source of variation accounted for over 90 % of total variation for every trait except GWE. Nevertheless, differences amongst DH lines were also highly significant for nearly every trait (ABS/CS_m_, TR_o_/CS_m_ and DI_o_/CS_m_ only *P* < 0.02). In addition, highly significant interactions between DH line and year were present for every trait. *F*-ratios for variation amongst DH lines were significant and around 2 for all traits except SPAD readings, and the productivity traits GWE and DWP, which showed more variability amongst DH lines. Despite the considerable year-to-year variation, broad-sense heritability (*h* ^2^) estimates for mean values across environments were reasonable, varying between 34.1 % (YP) and 68.2 % (SPAD). Trait heritability estimates each year (data not presented) varied from 36.3 % (DI_o_/CS_m_ in 2011) to 96.0 % (PI in 2007), and only seven of 45 trait × year combinations had heritabilities below 60 %.

**Table 1:** *F*-ratio, percentage variance and significance levels for line, year, and line × year interaction, and heritability (*h* ^2^ in %) for 13 traits measured in doubled haploid lines of *T. aestivum* cultivated in optimal conditions for 4 years: *F*_v_/*F*_m_ (the maximum photochemical efficiency), PI (overall performance index of PSII photochemistry), ABS/CS_m_ (light energy absorption), TR_o_/CS_m_ (amount of excitation energy trapped in PSII reaction centres), ET_o_/CS_m_ (amount of energy used for electron transport), DI_o_/CS_m_ (energy amount dissipated from PSII), RC/CS_m_ (number of active reaction centres), Chl_a+b_ (total chlorophyll *a* + *b*), Car (carotenoids), SPAD (chlorophyll meter readings), GWE (grain weight per ear), YP (grain yield per plant), and DWP (dry weight per plant)

	*F*_v_/*F*_m_	PI	ABS/CS_m_	TR_o_/CS_m_	ET_o_/CS_m_	DI_o_/CS_m_	RC/CS_m_	Chl_a+b_	SPAD^§^	Car	GWE	YP	DWP
*Line*													
*F*	1.8**	2.0**	1.6*	1.7*	1.9**	1.7*	2.0**	2.4**	3.0**	2.2**	3.48**	2.27**	3.07**
%	4.8	0.37	0.06	0.08	0.22	0.03	0.21	0.30	0.99	0.39	16.59	1.78	2.19
*Year*													
*F*	33.9**	542.2**	2953**	2222**	852.2**	6065**	928.0**	791.6**	300.6**	559.8**	15.3**	121.1**	132.6**
%	90.1	99.0	99.8	99.8	99.3	99.9	99.2	99.0	97.9	98.9	72.8	95.3	94.6
*Line* *×* *year*													
*F*	1.9**	3.6**	3.3**	3.2**	4.5**	2.4**	5.1**	5.2**	3.6**	4.0**	2.2**	3.7**	4.5**
%	5.1	0.67	0.11	0.14	0.52	0.04	0.55	0.65	1.16	0.71	10.59	2.89	3.2
Error (%)	1.39	0.05	0.01	0.01	0.03	0.01	0.02	0.02	0.09	0.04	2.22	0.22	0.16
*h* ^2^	45.4	50.5	43.5	44.0	48.3	43.9	49.7	61.6	68.2	58.5	54.2	34.1	44.0

*Significance levels* * *P* ≤ 0.02; ** *P* ≤ 0.001, *F* Snedecor–Fisher coefficient

^§^Measured only in 2010 (E_III_) and 2011 (E_IV_)

As shown in the ANOVA, significant trait variation (summarized for parents and DH lines in Table 2) was determined by both genotype and year. In general, the photosynthetic apparatus for CS was more active and functioned better than that for SQ1, resulting in significantly higher ABS/CS_m_ (characterizing light energy absorption), TR_o_/CS_m_ (amount of excitation energy trapped in PSII reaction centres) and Et_o_/CS_m_ (amount of energy used for electron transport). PI (overall performance index of PSII photochemistry) and RC/CS_m_ (number of active reaction centres) also had trends for higher means in CS, though similar amounts of energy were dissipated as heat from PSII (DI_o_/CS_m_) for both parents. Although concentrations of Chl_a+b_ and carotenoids did not differ significantly between the parents overall, SPAD meter readings averaged over 2010 and 2011 were similar for both parents, though CS accumulated significantly greater biomass (DWP) and grain yield (YP) overall than SQ1.

**Table 2:** Phenotypic performance for traits related to chlorophyll *a* fluorescence parameters, SPAD reading, chlorophyll and carotenoid contents, grain weight per ear, grain yield per plant and dry weight per plant of doubled haploid lines and their parents

Traits	Parents (mean ± SD)		DH lines				
	CS	SQ1	Mean	Min	Max	Skewness	Kurtosis
*F*_v_/*F*_m_	0.82 ± 0.01	0.81 ± 0.01	0.82 ± 0.01	0.80	0.84	−0.612	0.646
PI	3.12 ± 1.34	2.65 ± 1.23	3.05 ± 1.25	0.65	4.13	−0.110	0.472
ABS/CS_m_	3,061 ± 776	2,958 ± 866*^a^	3,068 ± 687	2,633	3,349	−0.213	0.673
TR_o_/CS_m_	2,512 ± 639	2,408 ± 734*	2,524 ± 573	2,112	2,800	−0.276	0.399
ET_o_/CS_m_	1,483 ± 423	1,376 ± 483*	1,483 ± 358	1,066	1,748	−0.491	0.836
DI_o_/CS_m_	549.0 ± 139.0	549.9 ± 135.0	543.3 ± 119.8	500.5	580.5	0.301	0.549
RC/CS_m_	1,304 ± 322	1,213 ± 387	1,274 ± 301	950	1,513	−0.438	0.759
Chl_a+b_	7.87 ± 4.8	9.60 ± 5.51	8.27 ± 4.19	4.10	12.60	−0.049	0.274
SPAD	40.1 ± 11.7	45.0 ± 1.6	41.5 ± 6.2	28.7	54.2	−0.339	−1.601
Car	1.13 ± 0.64	1.53 ± 0.91	1.13 ± 0.65	0.57	1.87	−0.004	−0.296
DWP	7.09 ± 1.56	4.81 ± 0.29***	6.03 ± 1.85	3.04	8.85	−0.175	−0.260
GWE	1.00 ± 0.02	0.91 ± 0.25	0.97 ± 0.32	0.39	1.64	0.229	−0.116
YP	3.38 ± 0.94	2.64 ± 0.14*	2.93 ± 1.01	1.18	4.64	0.015	−0.227

*Significance levels* * *P* ≤ 0.05; ** *P* ≤ 0.01; *** *P* ≤ 0.001

^a^Significance of differences between parents

For many traits, transgressive segregation amongst DH lines was apparent, as ranges amongst DH lines were much greater than differences between the parent means (Table 2). Means and ranges for each experimental year (Table S3) confirmed the presence from year to year of transgressive segregation for all the agronomic traits and pigment contents, as well as several of the fluorescence traits, particularly PI. Of the fluorescence parameters, *F*_v_/*F*_m_ (maximum photochemical efficiency) varied least, differing overall by only 1 % amongst years, and only 4–7 % amongst DH lines from year to year. In contrast, PI (overall performance index of PSII) varied over 2.5-fold overall amongst years and nearly threefold (2.4–3.2) amongst DH lines. Other fluorescence parameters were consistent in varying from year to year around 60–80 % overall, with genotypes varying from around 18 % (DI_o_/CS_m_—energy amount dissipated from PSII) to 64 % (Et_o_/CS_m_—amount of energy used for electron transport). ABS/CS_m_, TR_o_/CS_m_, Et_o_/CS_m_, DI_o_/CS_m_ and RC/CS_m_ were consistently higher in 2007 and 2008 than in 2010 and 2011 (Table S3).

Total chlorophyll content (of which chlorophyll *b* comprised around 27 %; data not presented) of DH lines varied around threefold both between years and between DH lines, and SPAD meter readings varied about 20 % between 2010 and 2011, with a range amongst DH lines of nearly twofold in both years (Table S3). DH line carotenoid contents varied between years by several-fold, from 3.0 in 2007 to 3.5 in 2008 and 2010, with carotenoid contents being much lower overall in 2008.

Regarding plant productivity, above-ground dry weight per plant at harvest (DWP) of CS was greater each year (by 47 % averaged over 2007–2010, E_I_–E_III_) than that of SQ1. However, CS was less efficient than SQ1 at converting biomass into yield, with overall grain weight per ear (GWE) and yield per plant (YP) for CS being, respectively, only 10 % (non-significant difference) and 28 % (difference significant at *P* < 0.05) greater than for SQ1. DH lines varied considerably in plant productivity, with a threefold range in DWP, and over fourfold ranges in GWE and YP, respectively.

As skewness for trait frequency distributions averaged across years (Table 2) and for individual years (Table S3) was less than 1.0 in absolute terms and trait kurtosis was typically less than 1.0 in absolute terms, trait data each year approximated normal distributions and were therefore suitable for QTL mapping without transformation.

#### Trait associations

Correlation coefficients between traits are given in Table 3. All fluorescence parameters were significantly positively correlated with each other each year, except for DI_o_/CS_m_ which was highly significantly correlated with ABS/CS_m_, TR_o_/CS_m_, ET_o_/CS_m_ and RC/CS_m_ in some years but not others. In 2010 (E_III_), the correlation between DI_o_/CS_m_ and *F*_v_/*F*_m_ was significantly negative. Similar highly significant positive correlations between fluorescence parameters were present in 2011, except for DI_o_/CS_m_, which was significantly negatively correlated with *F*_v_/*F*_m_ and PI (data not presented). The overall PSII performance index (PI) was most strongly correlated each year with ET_o_/CS_m_ and, as expected from the simple mathematical relationship between ABS/CS_m_, TR_o_/CS_m_ and DI_o_/CS_m_, ABS/CS_m_ was most strongly correlated with TR_o_/CS_m_ each year.

**Table 3:**
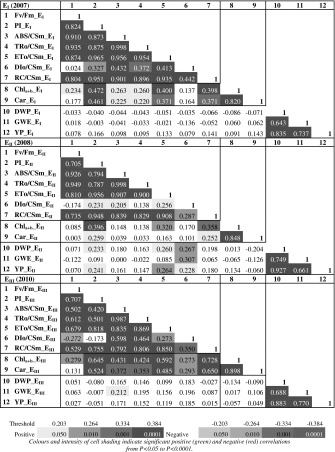
Correlation matrix using phenotypic mean values for the 12 traits studied in three experiments

Value in italics denotes significant negative correlation coefficient

Chl_a+b_ content was highly significantly positively correlated with carotenoid contents each year, and contents of both Chl_a+b_ and carotenoids were highly significantly positively correlated with PI, ET_o_/CS_m_ and RC/CS_m_ each year, except carotenoids in 2008 (E_II_). The three measures of plant productivity were highly significantly positively correlated with each other each year, but productivity traits were not consistently correlated with any fluorescence or pigment traits. The most significant correlations were in 2008 (E_II_), the high temperature year, between both DWP and GWE and DI_o_/CS_m_, and between YP and ET_o_/CS_m_, both significantly positive at *P* < 0.01. Other weakly significant correlations between productivity traits and CF parameters (PI, ET_o_/CS_m_ and DI_o_/CS_m_) were also specific to 2008 (Table 3).

### Genetic map and QTL analyses

#### DArT-extended genetic map

DArT analysis gave 1,581 additional DArT polymorphic markers, of which 853 had previously established locations on chromosomes. Of these DArT markers, 1,445 were used to complement the existing map based on 567 RFLP, simple sequence repeat (SSR) and amplified fragment length polymorphism (AFLP) markers (Habash et al. 2007). DArT markers, of which 446 were derived from wheat (wPt), 22 from triticale (tPt) and four from rye (rPt), enriched the map with 472 unique extra loci (Supplementary Fig. S2(a-c). The remaining 973 DArT markers gave allele scores with the DH lines identical to existing markers.

Combining these 472 DArT markers with the 567 existing markers extended the map length from the previous 3,522 cM (Quarrie et al. 2005; Habash et al. 2007) to 4,026 cM. Thus, doubling the number of markers resulted in a 504-cM increase in the map length, corresponding to 14 %. The longer map resulted from adding new loci either distally or internally to the existing genetic map. New loci mapping distally were responsible for an 182.6-cM (36 %) increase in length of the new map. Thus, the A, B and D genome linkage groups increased, respectively, by 90.3, 204.6 and 210.1 cM. Extending coverage of the CS × SQ1 map with unique locus DArT markers decreased the average marker interval from 7.9 to 4.4 cM.

The A and B genomes had the greatest coverage (Table 4), with 322 and 379 unique marker loci, accounting for 1,238 and 1,409 cM, respectively, of the total map (3.8 and 3.7 cM per marker). The D genome had the poorest coverage. In particular, chromosome 4D was covered by only nine markers, one more than 4D on the Habash et al. (2007) map, and most chromosomes of the D genome, with the exception of 1D, were still characterized by large gaps (exceeding 30 cM) between adjacent markers in linkage groups.

**Table 4:** Characteristics of linkage groups for CS × SQ1 genetic map after the integration of DArT marker loci

Chromosome	Markers per chromosome			Length (cM)
	All	Unique	DArT	
1A	134	46	26	137.2
2A	63	43	18	196.5
3A	61	39	23	170.7
4A	84	49	21	161.3
5A	70	53	22	220.0
6A	146	44	28	151.0
7A	110	48	20	201.6
Genome A	668	322	158	1,238.3
1B	92	50	29	191.8
2B	108	55	27	195.9
3B	157	87	53	232.7
4B	37	25	8	141.6
5B	95	59	29	230.2
6B	91	50	27	163.6
7B	69	53	34	253.6
Genome B	649	379	207	1,409.4
1D	101	39	26	188.9
2D	81	45	25	207.0
3D	132	30	12	212.5
4D	9	7	2	141.4
5D	43	32	8	262.2
6D	38	21	11	169.4
7D	172	45	23	197.6
Genome D	576	219	107	1,379.0

Distorted marker segregations occurred on all chromosomes except 6A and 5D. Thirty markers displaying segregation distortion in favour of CS alleles mapped on chromosomes 1A, 1D, 2A, 2B, 2D, 4D, 6B, 6D and 7A. Fifty-four markers segregated in favour of SQ1 alleles on chromosomes 1B, 2D, 3A, 3B, 4A, 4B, 5B and 7D. Additionally, chromosomes 3D, 5A and 7B showed skewed marker segregations towards both CS and SQ1 alleles.

The addition of DArT markers and use of JoinMap 4 software instead of MapMaker, used to construct the original maps of Quarrie et al. (2005) and Habash et al. (2007) (where single data of double *crossing over* were not always replaced with missing data), resulted in reordering of some marker loci in comparison with the Habash et al. (2007) map on 16 chromosomes, with only chromosomes 1A, 3B, 4B, 6B and 6D being unaffected. Most differences resulted from rearrangement of pairs of markers previously adjacent in the 2007 map. More extensive marker rearrangements compared with the 2007 map were on chromosomes 1D, 2A and 7A, representing distances of 27, 36 and 19 cM, respectively.

For QTL analysis, the 472 DArT markers were used with existing markers on the CS × SQ1 genetic map of Habash et al. (2007), where redundant loci and other loci of low information content had been excluded, to give in total a genetic map with 920 markers, varying from 87 loci on chromosome 3B to only seven loci on chromosome 4D.

#### Marker and gene deletion bin assignments

A total of 434 markers, averaging 21 markers per chromosome (ranging from six on 4D to 49 on 3B), were assigned to chromosome deletion bins on the basis of previously-published work either on their direct location to deletion bins or because they occurred between flanking markers that had previously been assigned to deletion bins. These allowed the deletion bin assignments to be inferred for a further 516 markers. Markers with known deletion bin assignments are identified in Supplementary Figure S3. Only 46 markers could not be reliably assigned to a deletion bin. Note that some redundant markers in the genetic map were included if they had been previously assigned to a deletion bin.

In total, 165 genes for photosynthetic light reactions, pigment metabolism and biomass (carbohydrate) accumulation were assigned to deletion bins on the A, B and D genomes (61, 53 and 51, respectively). Nearly half of them (61) could be given actual or estimated locations on the genetic map within 10 cM. The remainder were assigned in Figure S3 to the middle of deletion bins. Genes were divided amongst 57 associated with photosynthetic light reactions (15 gene types replicated on chromosomes and across genomes), 66 (21 gene types) associated with aspects of chlorophyll and carotenoid synthesis and metabolism, and 42 (eight gene types) involved with plant productivity traits, mainly carbohydrate synthesis and transport. The choice of genes was based largely on the availability of wheat expressed sequence tags (ESTs) that had previously been assigned to deletion bins. Genes and their method of assignment to deletion bins are given in Supplementary Table S2. An additional 38 known-function genes and proteins were already located as either protein-, RFLP- or PCR-based markers directly on the genetic map.

**Fig. 1:**
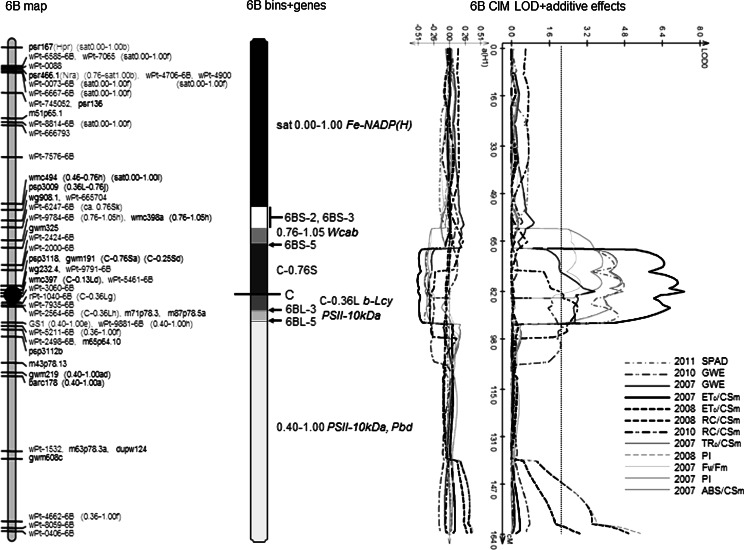
See Supplementary Figure S3 for a color version of chromosome 6B and the other 20 chromosomes. Maps for chromosome 6B, divided into the genetic map (*left*), the deletion bin map, break points and gene locations (*center*) and CIM LOD and additive traces for traits (*right*). Explanations for the genetic map are as described for Figure S2. Markers that were assigned to specific deletion bins are identified with the fraction length for the bin, followed by a lower-case letter indicating how the bin was identified (explained in Supplementary Table S1). *C*, *S* and *L* on bin fraction lengths indicate the chromosome centromere, short arm and long arm, respectively. On the *center map*, chromosome break points and bin fraction lengths are selected according to Sourdille et al. (2004). The *white area* indicates uncertainty in the location of the break point. The centromere is identified as a *black line* crossing the chromosome. Genes (explained in Supplementary Table S2) are located to the middle of the relevant bin. Genes are colored according to type of function: photosynthetic light reactions—*blue*, chlorophyll and carotenoid synthesis and metabolism—*red*, and biomass (carbohydrate) productivity—*green*; gene abbreviations are explained in Supplementary Table S2. The *right-hand map* shows the CIM LOD output only for those traits giving a LOD maximum approaching 1.8 or more. The *dotted black line* indicates a LOD score of 2.0. Underneath the LOD traces, the additive effects are shown as fractions of ±1 standard deviation. Additive effects show the direction of the QTL: positive where the Chinese Spring allele, and negative where the SQ1 allele increased the trait. (Color figure online)

An example of markers and genes assigned to deletion bins is shown for chromosome 6B in Fig. 1, for comparison with 6B of the earlier CS × SQ1 genetic map presented in Habash et al. (2007) (Fig. 1 and ESM3). The complete set of 21 chromosomes in color is illustrated in Supplementary Figure S3.

#### QTL analyses

The locations of QTL were identified using two methods: SMA and CIM (Supplementary Table S4). The criterion of declaring a QTL significant according to 1000 permutations identified only 82 QTL across the 13 traits and four experimental years, which equated to only 1.8 QTL/trait/year, varying from only 11 QTL in 2011 to 29 in 2007, and with 24, 36 and 22 significant QTL on the A, B and D genomes, respectively (QTL identified with “*” in Supplementary Table S4). Because of this relatively low number of QTL, the threshold LOD score for describing a QTL in Table S4 was reduced to 2.0. This increased the total number of QTL to 194 (4.3 QTL/trait/year), varying from 24 QTL in 2011 to 65 in 2007 (Table S4). Most QTL were located on the B genome, having 81, compared with 53 and 60 on the A and D genomes, respectively.

The distribution of QTL among the three types of trait was 120, 40 and 34 for CF, pigment and productivity traits, respectively. QTL for CF traits were most numerous on chromosome groups 5 (26), 6 (23) and 2 (22), while chromosome groups 3, 4 and 7 had least with only 11, 12 and 12 QTL, respectively, for CF traits. Pigment QTL were most numerous on chromosomes 3 and 4 (seven), while the group 1 chromosomes had only four QTL. The chromosome group with the most QTL for productivity traits was group 7, with 11 QTL across the three traits. Chromosome groups 3 and 5 each had only two productivity trait QTL. For individual chromosomes, 5B (18) and 6B (23) had the most QTL and chromosomes 3B and 7B had only two that reached the 2.0 LOD threshold (Supplementary Table S4 and Fig S3). Chromosomes 2B, 2D, 3A, 4B, 7A and 7D had over 10 QTL per chromosome.

Across the 4 years, the number of QTL for each of the seven CF parameters varied from only 13 for *F*_v_/*F*_m_, distributed amongst nine chromosomes, to 23 QTL on 14 chromosomes for PI. For the three pigment traits, 17 QTL were identified for carotenoids, with the majority distributed on six of the D-genome chromosomes. This compared with 13 QTL for SPAD readings and 10 for Chl_a+b_. The three productivity traits gave similar numbers of QTL, with 13, 11 and 10 for DWP, GWE and YP, respectively, with only one D-genome QTL for each trait.

The maximum LOD score achieved was 7.4 (ET_o_/CS_m_ in 2007 on chromosome 6B), and 15 QTL (12 for CF traits and three for pigment traits) gave QTL LOD scores over 5.0. These QTL also contributed the most to trait phenotypic variation (*R* ^2^), viz. 14.4–24.6 % (Table S4). Ten other QTL also had *R* ^2^ of at least 15 %, with the highest reaching 32.0 % (carotenoids in 2010 on chromosome 3D). Although maximum LOD scores for productivity traits were generally low (2.1–4.6), QTL for both DWP (on 4B in 2007) and YP (on 1B in 2007 and 7A in 2010) contributed over 14.6 % to total phenotypic variation. The software failed to provide *R* ^2^ and additive information for a few QTL, frequently at the ends of linkage groups.

The direction of QTL effects contributed by CS and SQ1 alleles was overall the same: 97 and 97 with increases due to CS and SQ1 alleles, respectively, indicating essentially uniform dispersion between the two parents of genes with increasing effects. This was also true for each of the three types of trait, with CS and SQ1 alleles increasing, respectively, 62 and 58 CF traits, 19 and 21 pigment traits and 16 and 18 productivity traits. There were, however, some differences between CS and SQ1 in the chromosomal distribution of QTL effects amongst the chromosome groups. Increases due to CS alleles predominated on the group 1 and 2 chromosomes, while SQ1 alleles predominated on the group 3 chromosomes.

CS alleles increased additive effects for every trait QTL on 1A, all but one QTL on 1B and four of seven QTL on 1D. Similarly, QTL additive effects were increased by CS alleles for every trait on 2A, ten of 12 QTL on 2B and eight of 15 QTL on 2D. The predominance of SQ1 alleles for QTL effects on the group 3 chromosomes was due entirely to 3D QTL, where increasing alleles for each of the seven QTL came from SQ1. On chromosome 4A, all increasing alleles for the five QTL were contributed by CS, while the opposite was true for 4B, where SQ1 alleles increased additive effects for ten of the 14 QTL. On 4D, the distribution between additive effects was four QTL for CS and two QTL for SQ1. SQ1 alleles increased additive effects more frequently than CS alleles for QTL on 5A and 5D (five of seven QTL on 5A and five of eight on 5D), while CS and SQ1 contributed equally to additive effects on 5B. Chromosome 6A gave only five QTL, two derived from CS, and three from SQ1 alleles. In contrast, almost all increasing allele effects came from SQ1 on chromosome 6B (20 of 23), the chromosome with the largest number of QTL overall, and all were from CS on 6D. Increasing additive effects were mainly from SQ1 on 7A (four from CS and 11 from SQ1 alleles) and were similarly distributed on 7D (six and five QTL from CS and SQ1 alleles, respectively). Chromosome 7B showed only two QTL, both with increasing alleles from CS. Chromosome 7A was notable in accounting for nine of the 31 QTL for productivity traits, compared with a maximum of only three productivity trait QTL on other chromosomes.

Using the 2.0 LOD threshold criterion, the large majority of QTL identified using CIM (167 of 194) were also identified as significant by SMA (shown in bold type in Table S4).

#### Coincidence of QTL

The number of regions per chromosome with coincident QTL varied from zero (3B and 7B) to six (5B, 5D and 7A), averaging nearly three per chromosome, and totalling 59. Chromosome 7A also had a further region where a QTL was coincident with one or more mapped genes. Of 82 regions of coincidence between trait QTL or between trait QTL and genes (see Tables 5 and S5 and Fig. S3) identified across the 21 chromosomes (summarized in Table 5, and in detail in Supplementary Table S5), 23 were coincidences between a single QTL and one or more genes.

**Table 5:** Summary of QTL coincidences with other QTL and/or with genes associated with photosynthetic light reactions (CF), chlorophyll and carotenoid synthesis and metabolism (Pig), and aspects of biomass (carbohydrate) productivity (Prod)

Chr	Region I				Region II				Region III				Region IV				Region V				Region VI				Region VII			
	cM	CF	Pig	Prod	cM	CF	Pig	Prod	cM	CF	Pig	Prod	cM	CF	Pig	Prod	cM	CF	Pig	Prod	cM	CF	Pig	Prod	cM	CF	Pig	Prod
1A	20.2	2			42.6	4			56.3	2																		
1B	32.5			2	90.3	1	2																					
1D	77.4	2			143.0		***1***		188.9	***1***																		
2A	77.2	***1***			83.2	***1***			106.1	***5***			180.8	2														
2B	4.5	***2***			108.4		1		133.5	***2***			155.5	4			169.2	4		3								
2D	129.5	***2***			159.2	1	***4***		174.0	2	***1***		200.4	6	***1***													
3A	4.5	1	***1***		21.1		1		64.5	3		1	102.5	2	2													
3B	–																											
3D	11.4	1	***2***	1	45.1		2		72.2	2		1																
4A	18.0		1		34.9	2			103.9	2			125.7	***5***														
4B	65.2	3	***1***	2	74.0		1	***2***	98.4	***2***			105.6	2	***1***		127.4		***2***									
4D	6.0			***1***	35.0	3	***1***		55.9			***1***	134.7		1													
5A	65.5			***1***	112.8	***1***		1	119.5		***2***		130.6	***2***	***1***		186.4	1										
5B	30.6	6			42.8	2			69.8	2			78.7	2			99.0	4	1		113.5	2						
5D	15.8		1		40.2		1		157.0	1	1		171.1	2			198.9	***1***			242.9	***2***						
6A	7.4			2	48.5	***1***			97.2	1																		
6B	71.7	5	1	1	81.9	***6***	1	1	87.5	***4***	1	2	163.6	***3***														
6D	154.6	***5***	2		166.8	***5***	2																					
7A	2.0	2			16.9	2			77.2	1	***1***		128.3	1		3	151.9			2	161.2			4	190.2	1		
7B	146.8			1	221.5			1	251.1			1																
7D	14.6		1		71.2	1	1		83.0	1	2		168.1	1			179.4	1	3									

QTL coincident with candidate genes are *underlined in italics.* QTL regions containing a single QTL *not underlined/in italics* were coincident with other non-candidate genes. cM indicates the distance from the top of the chromosome linkage group to the LOD max for the QTL with the greatest LOD max

Coincidences amongst QTL reflected the significant correlations between traits in the phenotypic correlation matrices for each year (Table 3). Thus, coincidence of QTL occurred mainly between CF traits. Of the 59 regions of coincidence involving multiple QTL, 24 involved QTL for only CF traits, and a further eight coincidences involved only QTL for pigment and/or productivity traits: 1B-I (productivity), 3D-II (pigments), 4B-II (carotenoids + productivity), 4B-V (pigments), 5A-III (pigments), 6A-I (productivity), 7A-V (productivity), and 7A-VI (productivity).

Comparison of QTL coincidences amongst the seven CF parameters gave useful insights into the relationships between them. Thus, overall performance index (PI) QTL were most frequently associated with those for ET_o_/CS_m_, and vice versa (17 coincident QTL regions). QTL for ABS/CS_m_ were most frequently associated with QTL for TR_o_/CS_m_, and vice versa, with 16 coincident QTL, essentially twice as many QTL coincidences as those with other CF parameters. QTL for RC/CS_m_ were most frequently associated with QTL for PI (11 coincident QTL regions). *F*_v_/*F*_m_ QTL were most frequently coincident with those for both ET_o_/CS_m_ and RC/CS_m_, with seven coincident regions each. Only DI_o_/CS_m_ gave QTL that were not frequently coincident with QTL for any of the other CF parameters: a maximum of only two coincident QTL regions with ABS/CS_m_, ET_o_/CS_m_, PI and RC/CS_m_. These findings support the frequently weak phenotypic correlations between DI_o_/CS_m_ and other CF parameters in Table 3. Nineteen QTL for CF parameters were also coincident with QTL for chlorophyll or SPAD readings. Of the CF parameters, PI QTL were most frequently coincident with those for Chl_a+b_ (ten QTL regions).

The large year-to-year trait variability and highly significant line × year interactions evident from the ANOVA (Table 1) led to relatively few QTL for a particular trait being coincident from year to year. Amongst the 13 traits, only fifteen QTL coincidences (LOD max ≥1.8) occurred for the same trait across years (Table S5). QTL for ABS/CS_m_ and TR_o_/CS_m_ were both coincident in 2007 (E_I_) and 2008 (E_II_) at QTL regions 1A-II and 2D-V. Only four other CF traits had coincident QTL across years: QTL regions 2A-III (ET_o_/CS_m_ in 2007 and 2011), 4B-IV (PI in 2007 and 2010), 5B-I (DI_o_/CS_m_ in 2007 and 2008) and 5B-II (DI_o_/CS_m_ in 2007 and 2008). QTL for pigment traits were only coincident for more than 1 year at 1B-I (SPAD in 2010 and 2011), 2D-II (SPAD in 2010 and 2011), 5A-III (Chl_a+b_ in 2008 and 2010), 6D-I and 6D-II (carotenoids in 2007 and 2010), and three QTL regions were coincident across years for only the productivity traits DWP (4B-I in 2008 and 2010, 4B-II in 2007 and 2010) and GWE (2B-V in 2008 and 2010, 6B-III in 2007 and 2010, and 7A-VI in 2007 and 2010).

The greatest number of coincident QTL was on chromosome 6B (6B-II, with QTL for eight traits: six CF, one SPAD and one GWE). Six other QTL regions were coincident for seven QTL (2B-V for four CF and three productivity, 2D-IV for six CF and Chl_a+b_, 6B-I including five CF and 6B-III four CF QTL, and 6D-I and 6D-II both for five CF and two carotenoid QTL).

About half the QTL for productivity traits (18 of 34) were coincident with one or more other trait QTL, varying from no coincident Chl_a+b_ QTL to six coincident QTL for ET_o_/CS_m_. It was clear that no CF parameter or pigment trait gave frequent QTL associations with any productivity trait. The cluster of QTL on 6B (6B-II), shown in Fig. 1, gave the largest number of trait QTL (seven) coincident with a productivity QTL (GWE). The major productivity trait yield per plant (YP) had four QTL coincident with those for biomass production (DWP), and three coincident with GWE, mainly on chromosome 7A.

Of the 82 regions of QTL coincidence (Tables 5 and S5), 62 were also coincident with one or more genes identified in Table S2: 123 genes in total (around two genes per QTL region). However, in most cases, genes were assigned only to a chromosome deletion bin, so it is unlikely that all would be located between the flanking markers delineating the QTL regions. The greatest number of genes identified to be coincident with one or more QTL regions was eight on chromosome 2A (2A-III), and 4A had seven (4A-IV). Forty-eight candidate genes, based on their known functions (underlined in Table S5), were identified to be potentially coincident with 56 % of the coincident QTL-gene regions (35 of 62), QTL region 5A-IV having candidate genes for both CF traits and Chl_a+b_.

Six candidate gene-QTL coincidences were present at homoeologous positions on two of the three genomes: for CF parameter QTL, 2A-III and 2D-I with photosystem I reaction centre subunit psaK (*PSI*-*K*), 2A-III and 2B-III with chlorophyll *a*/*b* binding protein CP24 10A (LHCP) (*Cab*-*10A*), 4A-IV and 4B-II with oxygen-evolving complex 25.6-kDa protein (*O*-*ec 25.6* *kDa*), 5A-IV and 5D-V/VI with photosystem-I F subunit precursor (*PSF*-*I*), 6B-I/II and 6D-I/II with photosystem II 10-kDa polypeptide (*PSII*-*10* *kDa*), and for the productivity trait DWP with sucrose transporter 1 (*Sut1*) on 4B-II and 4D-III.

For the example chromosome in Fig. 1 (6B), several QTL for CF parameters at QTL region 6B-II/III were coincident with a photosystem II 10-kDa polypeptide (*PSII*-*10* *kDa*) gene, a determinant of photosystem II JIP-test CF parameters (Strasser et al. 2000).

In total, 19 QTL regions had at least one coincident candidate gene involved in photosynthetic light reactions (CF parameters), 13 were candidate genes for aspects of chlorophyll and carotenoid synthesis and metabolism, and four were coincident with candidate genes for biomass accumulation (productivity).

## Discussion

Here we have presented, to our knowledge, the first detailed genetic analysis in bread wheat of CF parameters, including JIP-test parameters, which are easily measured indicators of the activity of the photosynthetic apparatus. Our objectives were to identify the location of QTL for these CF traits and to relate them to both pigment contents (chlorophylls and carotenoids) and measures of biomass productivity at both the phenotypic and genetic levels, to assess the importance of variation in CF traits to contributing to overall plant productivity. To improve the precision of the genetic analysis, we extended the existing genetic map from CS × SQ1 (Habash et al. 2007) by adding DArT markers, and located markers on the extended map to chromosome deletion bins. This allowed us to test for coincidence of QTL with several genes known to be involved with CF, pigment contents and biomass (carbohydrate) productivity.

### Phenotypic characteristics

Plant growth depends on photosynthesis. Contents of assimilation pigments and chlorophyll fluorescence parameters are important traits determining the health or integrity of the internal apparatus during photosynthesis within a leaf (Krause and Weiss 1991; Maxwell and Johnson 2000). Thus chlorophyll fluorescence characteristics are diagnostic for various aspects of the photosynthetic efficiency of plants, which will determine plant productivity and, for crop plants, ultimately yield. The chlorophyll fluorescence parameters typically used for determining photosynthetic activity in plants are minimum fluorescence (*F*_o_), maximum fluorescence (*F*_m_), variable fluorescence (*F*_v_ = *F*_m_ − *F*_o_) and maximum photochemical efficiency of PSII (*F*_v_/*F*_m_) (e.g. Zhang et al. 2010; Vijayalakshmi et al. 2010; Debabrata and Kumar 2011; Kumar et al. 2012). In addition to the widely used *F*_v_/*F*_m_ parameter, we studied the PSII performance index (PI) that is essentially an indicator of sample vitality, as well as the JIP-test parameters ABS/CS_m_, DI_o_/CS_m_, TR_o_/CS_m_, RC/CS_m_ and ET_o_/CS_m_ which can be used to explain the stepwise flow of energy through PSII at the cross-section for maximum fluorescence (CS_m_) level. ABS/CS_m_ describes the number of photons absorbed by the antenna molecules of active and inactive PSII reaction centres (RCs) over the excited CS_m_ of the sample, which are related to the chlorophyll concentration in the sample, as demonstrated by the highly significant positive correlations between RC/CS_m_, the density of reaction centres, and total chlorophyll (Chl_a+b_) each year (Table 3). TR_o_/CS_m_ is connected with the maximum trapping rate of an exciton measured over a CS_m_ of active and inactive RCs. DI_o_/CS_m_ describes the total energy dissipation measured over the sample CS_m_, which represents the difference between number of photons absorbed and those trapped. ET_o_/CS_m_ is the re-oxidation of reduced electron acceptors via electron transport over a CS_m_ of active and inactive RCs (Force et al. 2003). Photosynthetic PSII fluorescence is a valuable biosensing device for stress detection in plants that allows many samples to be screened in a short time (Strasser et al. 2000).

Our results showed extensive highly significant correlations between the seven CF parameters in our study, with the exception of DI_o_/CS_m_, which was only occasionally correlated with other CF parameters (Table 3). Although the experimental conditions for growing plants under control (well-watered) conditions were designed to be the same each year, it is clear from the ANOVA (Table 1) and mean data for each year (Table S3) that CF parameters differed considerably between the 4 years, and this was reflected in large differences in pigment concentrations between years. In particular, PI was much lower in 2008 and 2010 compared with other years, and this coincided with lower pigment contents in those years. It is difficult to explain this by either temperature or growth stage at sampling time, as 2008 and 2010 differed markedly in both temperature (12 °C higher in 2008 than in 2010 for 10 days prior to sampling) and growth stage (around anthesis in 2008 compared with a week into grain filling in 2010) on the sampling dates for CF. Plant nutrition may have influenced leaf chlorophyll contents and thereby PI and other CF parameters, though neither soil nitrogen nor phosphorus was monitored during these trials. Both low soil nitrogen and low phosphorus nutrition have been shown to reduce PI (Malceva et al. 2011; Ripley et al. 2004, respectively). Lower PI and pigment contents in 2008 and 2010 were also reflected in lower biomass (DWP) of DH lines than in 2007 (Table S3).

In our studies, wheat productivity was represented by above-ground biomass (DWP), grain weight per ear (GWE) and yield per plant (YP). Although the DH population showed significant variation in these parameters, our results showed that CF parameters were not usually correlated with measures of plant productivity. Similar findings were presented for another wheat mapping population by Liang et al. (2010) studying dry mass accumulation (DMA) and yield traits in relation to photosynthesis. They also found a poor correlation of DMA with *F*_v_/*F*_m_.

A trait that can be used as a reliable indicator for photosystem PSII efficiency is total chlorophyll content (Vijayalakshmi et al. 2010). Flag leaf chlorophyll contents around flowering time were positively associated with yield in near-isogenic lines (NILs) of wheat selected by Quarrie et al. (2006) for a yield QTL on chromosome 7AL. Flag leaf chlorophyll contents in the CS × SQ1 DH lines were also significantly positively correlated with both grain yield and plant biomass in field trials (Quarrie et al. 1995 and unpublished), but only in a low nitrogen treatment. Thus, maintaining a higher content of chlorophyll in crops may be an effective strategy to increase biomass production and grain yield (Wang et al. 2008).

The efficiency of PSII also depends on the concentration of carotenoids (Sarijeva et al. 2007; Tanya et al. 1994), and PI in our experiments was significantly correlated with carotenoid contents each year (Table 3). The content of carotenoids is important not only for their essential role in the photosynthetic process, but also for their significant role in photoprotection of photosynthetic membranes against the large amounts of solar energy absorbed by photosynthetic pigments (Havaux and Niyogi 1999; Asada 1999).

### The extended genetic map of Chinese Spring × SQ1 and bin assignments

The addition of 472 DArT marker loci to the CS × SQ1 genetic map substantially improved the marker density of the map. In total, 1,445 DArT markers were added to the CS × SQ1 genetic map, of which 472 identified unique recombination events. The number of DArT markers mapped onto the CS × SQ1 population, reflecting the level of polymorphism between CS and SQ1, was relatively high when compared with other genetic maps. For example, 336 DArT markers were mapped on Cappelle-Desprez × Palmiet (Agenbag et al. 2012), 632 in a Rio Blanco 9 × IDO444 winter wheat population (Chen et al. 2012), and 296 DArT markers were mapped on a Kariega 9 × Avocet S doubled haploid (DH) mapping population (Prins et al. 2011). The high efficiency in identifying DArT polymorphisms would be due to the choice of parents for the cross. Chinese Spring is an old landrace, reference wheat with a pedigree very distant from that of the other parent SQ1, which was extracted from a cross between a UK spring wheat variety and an advanced breeding line (Quarrie et al. 2005).

DArT clusters were found on all wheat chromosomes except 4B, where markers were evenly distributed, and 4D, where only two DArT markers were mapped. Grouping of markers around centromeres was found on chromosomes from homoeologous groups 1 and 3, and chromosomes 5A, 2B, 5B, 6B, 2D and 7D. This is a frequent feature of wheat maps, as discussed in Quarrie et al. (2005).

Low coverage of the D genome compared with the A and B genomes has often been observed (e.g. Prins et al. 2011, Chen et al. 2012 for recent genetic maps). Chromosome 4D is particularly recalcitrant to genetic mapping and no linkage group corresponding to this chromosome was found in the mapping study of Agenbag et al. (2012) using Cappelle-Desprez × Palmiet. Similarly, only two DArT markers were found on 4D of the population Rio Blanco 9 × IDO444 (Chen et al. 2012), which included wPt-5809, also present in the CS × SQ1 population.

The CS × SQ1 mapping population has only 95 DH lines, of which 94 were used in these studies. Although this is not large in genetical terms, with recent wheat mapping using DH line populations of typically at least 150 genotypes (e.g. McCartney et al. 2005; Yang et al. 2005, 2007b; Laperche et al. 2006), it is large enough as a first screen for locating QTL regulating traits. It is also similar in size, though not in meiotic events, to a recent study by Zhang et al. (2010) on locating QTL for chlorophyll fluorescence traits in wheat, where 114 recombinant inbred lines were used (see discussion below).

Locating markers to the Chinese Spring deletion bins exemplified the frequent finding of greater recombination frequencies towards the telomeres of chromosome arms, and several proximal deletion bins assigned high numbers of markers in Sourdille et al. (2004) by mapping directly to deletion bin stocks had very few markers and small map distances in the CS × SQ1 genetic map. For example, 2B deletion bin centromere-2BL-2 (C-0.36L, Figure S3) had 39 % of all mapped 2B markers in Sourdille et al. (2004), but represented at most only 2.8 % of the CS × SQ1 2B genetic map length. Other deletion bins were devoid of markers on the CS × SQ1 genetic map. This exercise illustrated the weakness of using random DNA AFLPs as markers, because of the difficulty in reproducing AFLP bands across mapping populations. Thus, isolated AFLP markers could not be assigned to deletion bins.

Markers assigned to deletion bins revealed occasional inconsistencies between the map order of markers and deletion bin assignments. Sometimes a marker, such as cfd73 on chromosome 2D, had been independently assigned to different deletion bins. Chromosomes 5A and 5D had particularly problematic regions. On 5A short arm, the order of bin assignments of DArT markers implied an inversion compared with the genetic map, and on 5D long arm the orders of several mapped markers and deletion bins were inconsistent.

Despite the high density of markers on chromosome 3B, it was still not possible to resolve all the deletion bins used by Sourdille et al. (2004), and SSR marker locus psp3112a did not locate to the expected deletion bin.

### Locations of QTL and their coincidences

In the present study, numerous QTL for the CF parameters *F*_v_/*F*_m_, PI, ABS/CS_m_, TR_o_/CS_m_, ET_o_/CS_m_, DI_o_/CS_m_ and RC/CS_m_ were detected in four environments, with QTL widely distributed across the three genomes. QTL for pigments, Chl_a+b_, Car and SPAD and for agronomic traits DWP, GWE and YP were also detected in three environments. The assignment to chromosome deletion bins of markers and several genes for photosynthetic light reactions, chlorophyll and carotenoid metabolism, as well as a smaller number of other genes involved in biomass (carbohydrate) accumulation, facilitated the comparison of genes with QTL effects to identify candidate genes for trait QTL (Table S5).

Although QTL were present on every chromosome, they were not uniformly distributed amongst chromosome groups and QTL numbers differed markedly from chromosome to chromosome. For CF parameters, individual chromosomes had from 23 QTL (6B) to only one on 3B and none on 7B. CF parameter QTL (including those with LOD scores of 1.8 or 1.9) varied from 26 on the group 5 and 23 on the group 6 chromosomes to only 11 on the group 3 chromosomes. For chromosome groups, the frequencies of CF parameter QTL reflected the relative frequency of mapped genes associated with CF parameters. Using the bin-mapped wheat ESTs (http://wheat.pw.usda.gov/cgi-bin/westsql/map_locus.cgi), the numbers of ESTs for proteins with expected roles in photosynthetic light reactions were highly significantly correlated with the number of CF QTL per chromosome group listed in Table 5 (*r*_5*df*_ = 0.93, *P* = 0.002), varying from only two ESTs on the group 3 chromosomes to 21 and 16 ESTs on the group 5 s and group 6 s, respectively. As half the CF QTL regions were associated with at least one candidate gene, it is possible that several of these would be the functional gene regulating those QTL.

Several of the fluorescence and chlorophyll traits have previously been analysed in wheat. Thus, we located QTL for *F*_v_/*F*_m_ on chromosomes 2A, 3A, 6A, 7A, 2B, 5B, 6B, 1D and 2D. On chromosome 2A the QTL *QF*_*v*_ */F*_*m*_ *.igdb*-*2A* was detected during dark-induced senescence by Li et al. (2012) in a wheat DH population. Comparative mapping shows this QTL to be close to our QTL for *F*_v_/*F*_m_ *QF*_*v*_ */F*_*m*_ *.csdh*-*2A* identified near marker *Xgwm339* by both CIM and SMA. In the same study, Li et al. (2012) also located QTL on 6B and 7A (*QF*_*v*_ */F*_*m*_ *.igdb*-*7A*) which appear to be at the same chromosomal regions as our QTL *QF*_*v*_ */F*_*m*_ *.csdh*-*6B.1* and -*6B.2* and *QF*_*v*_ */F*_*m*_ *.csdh*-*7A.2*. A QTL associated with *F*_v_/*F*_m_ at anthesis stage was detected by Liang et al. (2010) on chromosome 6A close (about 10 cM difference) to the position of the QTL for *F*_v_/*F*_m_ detected in our studies. Although Vijayalakshmi et al. (2010) detected a QTL for *F*_v_/*F*_m_ on chromosome 7A in a recombinant inbred line population of winter wheat under high temperature conditions, the position of this QTL appears to be different from our two QTL for *F*_v_/*F*_m_ on 7A.

QTL for Chl_a+b_ were detected on chromosomes 3A, 5A, 3B, 4B, 2D and 7D. On chromosome 5A a QTL associated with chlorophyll content was reported in Ribaut (2006) and Zhang et al. (2010) found a QTL significantly affecting chlorophyll content on chromosome 4B. QTL for chlorophyll *b* content at different developmental stages were also identified *inter alia* on chromosomes 5A and 2D by Zhang et al. (2009).

Ten QTL were identified for several traits on three regions of chromosome 2D. In two regions (2D-II and 2D-III), QTL were present for Chl_a+b_, as well as SPAD at 2D-II. We also identified significant QTL for both chlorophyll and carotenoid pigments at *Xgwm539* (2D-I) by SMA in several years. Interestingly, Verma et al. (2004) mapped several QTL for grain yield and the chlorophyll-related trait flag leaf senescence near the marker loci *Xgwm30* and *Xgwm539* on chromosome 2D in winter wheat, and the region is within 20 cM of major QTL for these traits identified using CIM (Table S5). QTL for chlorophyll content were also identified on chromosome 2D by Zhang et al. (2009, 2010), though comparative mapping suggested that neither of these chlorophyll QTL coincided with our 2D pigment trait QTL.

Three of the five regions where QTL for CF parameters and pigment traits were also coincident with QTL for measures of plant productivity were on 6B. Our studies identified a major region around the centromere on chromosome 6B for co-localization of six CF parameters amongst themselves and also with chlorophyll content (SPAD reading) and a measure of plant productivity (GWE). For all QTL, the increasing allele was contributed by SQ1. The multiple closely-spaced QTL LOD peaks around the centromere on chromosome 6B have been a feature of previous QTL analyses on the CS × SQ1 mapping population (Quarrie et al. 2005; Habash et al. 2007 and unpublished). We have preliminary evidence using sets of NILs selected independently for several markers in this region of 6B (Dodig, Barnes and Quarrie, unpublished) that these clusters of peaks may be resolved into at least two independent QTL, at least for several yield and productivity traits.

Li et al. (2012) located QTL on chromosome 6B for both *F*_v_/*F*_m_ and total chlorophyll that mapped near a QTL for grain protein content identified by Prasad et al. (2003). Although the genetic maps are not easy to compare, the QTL of Li et al. (2012) appear to span the same region where our highly significant QTL for CF parameters, pigments and GWE were located. In contrast to these results, Zhang et al. (2010) found no QTL for CF parameters on chromosome 6B. In Habash et al. (2007), using the same CS × SQ1 DH population, this region on chromosome 6B was shown to be highly significant for variation in several traits associated with plant productivity: flag leaf area, number of tillers, grain weight/ear, ear number/plant and grain size. In this case, SQ1 also contributed the increasing allele.

We also found 16 QTL on chromosome 4B for fluorescence traits, pigments and DWP using CIM. The QTL *QDWP.csdh*-*4B.1*, *QSPAD.csdh*-*4B.1*, *QRC.csdh*-*4B* and *QPI.csdh*-*4B.1* were located in the region of the major dwarfing gene locus, *Rht*-*B1*-*4B* (around 65 cM). This gene is one of the most important from a commercial perspective for reducing plant height in bread wheat (Araus et al. 2008). Also on chromosome 4B, Kumar et al. (2012) identified QTL near marker *Xcfa2149* for *F*_v_/*F*_m_ and for chlorophyll under stress. The majority of QTL we located around *Rht*-*B1*-*4B* were for plant productivity. QTL for measures of productivity in wheat at *Rht*-*B1*-*4B* have also been identified by others, for example Börner et al. (2002), Quarrie et al. (2005), McCartney et al. (2005) and Habash et al. (2007).

### Future developments

The environmental sensitivity of our phenotypic and QTL findings needs further analysis under a range of defined environments to establish more precisely the environmental parameters under which a specific QTL is expressed. As a first step in characterizing the influence of specific aspects of the environment on the genetic control of these photosynthesis-related traits, we are currently studying the influence of water availability.

Despite the inevitable link between efficiency of the photosynthetic apparatus and biomass accumulation, we failed to identify any strong association, either phenotypic or genotypic, between any CF parameter and productivity trait. Thus, future work will need to focus on establishing the most appropriate strategy for relating efficiency of the photosynthetic apparatus to plant productivity in this population of DH lines.

Information on the number and location of QTL for chlorophyll fluorescence kinetics, pigment contents and productivity traits is the first step on the road towards identifying genes regulating those traits. Ultimately, our QTL findings and the assignment of chromosome deletion bins to the CS × SQ1 mapping population will help geneticists to identify functions for genes as the structure and functional features of gene-rich regions of the wheat genome is gradually unravelled (Rustenholz et al. 2011).

## Conclusions

The major conclusions from this research may be summarized as follows:

- a large number of trait QTL, especially for CF parameters, were identified across the four study years. Elucidating the genetic control of physiological traits provides physiologists with a powerful tool to improve our understanding of the inter-relationships between traits. Here we have presented for the first time in wheat a QTL analysis of traits related to phenomenological energy fluxes per excited cross section (CS_m_), namely ABS/CS_m_, DI_o_/CS_m_, TR_o_/CS_m_, RC/CS_m_ and ET_o_/CS_m_, together with overall performance index of photosystem II (PI), key parameters determining efficiency of the photosynthetic apparatus.
- QTL for many traits were year-specific, probably reflecting differences between years in the seasonal weather and edaphic growth conditions. Thus, the significant interaction between genotype and year in the ANOVA showed the environment to be a major factor in determining the expression of genes for those traits.
- QTL for many fluorescence traits were coincident, implying the same genetic control, consistent with the derivation of JIP-test parameters.
- very few QTL for fluorescence or pigment traits were coincident with QTL for measures of plant productivity, implying that these traits, at the time they were measured, were not major determinants of biomass production or yield. Thus, our measurements of fluorescence parameters on a single occasion during plant development failed to demonstrate an obvious association between fluorescence and pigment traits and plant productivity on the basis of either phenotypic correlations or co-location of QTL.
- candidate genes were identified for several trait QTL, providing targets for future research on functional genetics.
- the allocation of markers on the CS × SQ1 genetic map to chromosome deletion bins will facilitate future QTL analysis of these and other traits to identify candidate genes.

## Electronic supplementary material

Below is the link to the electronic supplementary material.

Supplementary material 1 (DOC 104 kb)

Supplementary material 2 (DOC 294 kb)

Supplementary material 3 (DOC 115 kb)

Supplementary material 4 (DOCX 63 kb)

Supplementary material 5 (DOC 309 kb)

Supplementary material 6 (DOC 101 kb)

Figure S1 Mean decade temperature (a) and humidity (b) during the four growing seasons (2007 - E_I_, 2008 – E_II_, 2010 – E_III_ and 2011 - E_IV_). The position of dashed and solid arrows is based on mean dates across the four years (EPS 1074 kb) (DOC 46 kb)

Figure S2 Genetic map of Chinese Spring (CS) x SQ1 derived from 96 or 90 DH lines for the A (a), B (b) and D (c) genomes. Loci shown in blue are RFLP markers, loci in red are PCR-based markers, loci in green are DArT markers, loci in orange are morphological, physiological and isozyme markers, loci in black are AFLP markers. Black circles show the most likely positions of centromeres. DArT markers with previously-known chromosome assignments are identified with the chromosome assignment. To aid clarity, the prefix “X”, denoting markers of unknown function, is deleted from marker names (EPS 915 kb) (EPS 1038 kb)

Supplementary material 8 (EPS 1075 kb)

Supplementary material 9 (EPS 916 kb)

Figure S3 Maps for chromosomes 1A to 7D, divided into the genetic map (left), the deletion bin map, break points and gene locations (middle) and CIM LOD and additive traces for traits (right). On the genetic map marker types are identified by colour: AFLPs - black, RFLPs - blue, SSRs - red, known-function markers - orange, DArTs - green. Best estimates of the location of chromosome centromeres are indicated with a black circle. DArT markers with previously-known chromosome assignments are identified with the chromosome assignment. To aid clarity, the prefix “X”, denoting markers of unknown function, is deleted from marker names. Markers that were assigned to specific deletion bins are identified with the fraction length for the bin, followed by a lower case letter indicating how the bin was identified (explained in Supplementary Table S1). C, S and L on bin fraction lengths indicate the chromosome centromere, short arm and long arm, respectively. On the middle map, chromosome break points and bin fraction lengths are selected and coloured according to Sourdille et al. (2004). White areas indicate uncertainty in the location of the break point. Centromeres are identified as a black line crossing the chromosome. Genes (explained in Supplementary Table S2) are located either in the predicted part of the bin or in the centre of the bin if a more precise location was not identified. Genes are coloured according to type of function: photosynthetic light reactions - blue, chlorophyll and carotenoid synthesis and metabolism - red, and biomass (carbohydrate) productivity - green, gene abbreviations explained in Supplementary Table S2. The right-hand map shows the CIM LOD output only for those traits giving a LOD maximum approaching a maximum of 1.8 or more. The dotted black line indicates a LOD score of 2.0. Underneath the LOD traces, the additive effects are shown as fractions of ±1 S.D. Additive effects show the direction of the QTL: positive where the Chinese Spring allele, and negative where the SQ1 allele increased the trait (PDF 2859 kb) (PDF 5266 kb)
