# Supplementary material for: Quantitative trait loci for leaf chlorophyll fluorescence parameters, chlorophyll and carotenoid contents in relation to biomass and yield in bread wheat and their chromosome deletion bin assignments
Source: Mol Breed. 2013 Apr 10;32(1):189–210. doi: 10.1007/s11032-013-9862-8 (PMC3684715; doi:10.1007/s11032-013-9862-8)
Supplement: Supplementary file 1 — Supplementary material 1 (DOC 104 kb) [file 11032_2013_9862_MOESM1_ESM.doc]

**Quantitative trait loci for leaf chlorophyll fluorescence parameters, chlorophyll and carotenoid contents in relation to biomass and yield in bread wheat and their chromosome deletion bin assignments**

Czyczyło-Mysza I.^1^, Tyrka M.^2^, Marcińska I.^1^, Skrzypek E.^1^, Karbarz M.^3^, Dziurka M.^1^, Hura T.^1^, Dziurka K.^1^, Quarrie S.A.^4^

^1^ The *F. Górski* Institute of Plant Physiology, Polish Academy of Sciences, Kraków, Poland

^2^ Rzeszów University of Technology, Department of Biochemistry and Biotechnology, Poland.

^3^ Institute of Applied Biotechnology and Basic Sciences, University of Rzeszow

^4^ Faculty of Biology, Belgrade University, Serbia, and Visiting Professor, Newcastle University, UK.

Corresponding author: [czyczylo-mysza@wp.pl](javascript:oknoAdresat('napisz.html?to=czyczylo-mysza@wp.pl',10,10,650,540,1);)

**Table S1.** Sources of information for locating markers to chromosome bins.

| Chromosomes 1A to 4A | |
| --- | --- |
| Code letter | Source of information for locating markers to chromosome bins |
| a | Sourdille et al. (2004) |
| b | Erayman et al. (2004) and ESM chromosome groups 1-7. |
| c | ITMI chromosome group maps (groupe1v2.xls - groupe7v2.xls): http://wheat.pw.usda.gov/ggpages/SSRclub/GeneticPhysical/ |
| d | Goyal et al. (2005) s10142_2005_146_ESM_supp.pdf |
| e | Bin-mapped wheat ESTs (http://wheat.pw.usda.gov/cgi-bin/westsql/map_locus.cgi) |
| f | CerealsDB mapped DArT markers (http://www.cerealsdb.uk.net/CerealsDB/Documents/DOC_DArT_index.php) |
| g | Francki et al. (2009) 438_2008_403_MOESM1_ESM.ppt |
| h | Marone et al. (2012) |
| i | wmc278: maps to gwm164 (C-0.17L ref a: Sourdille et al., 2004) in Genc et al. (2010). |
| j | Glu-A1: proximal to gwm164 (C-0.17L ref a) (http://www.wgin.org.uk/wgin_2003-2008/Resources/271004AssociatedDocuments.pdf) |
| k | wPt-664666: maps proximal to wmc93 (C-0.17L ref c: ITMI group 1 maps) in the WY-Ch map of Cui Fa PhD thesis (2011): http://xwb.sdpec.edu.cn/sdadgei/paper/B2008037崔法.pdf. |
| l | wPt-664972=wPt-664968: located in SMT Ghaffary PhD thesis (2011) (http://edepot.wur.nl/169465) 2.7 cM from gpw2180 (0.17-0.61 ref c: groupe1v2.xls). |
| m | wPt-733091: maps distal to wPt-7030 (0.61-1.00 ref f: CerealsDB mapped DArTs) in composite DArT map (Killian, unpublished). |
| n | wPt-8016: flanked by bcd265b and ksuG34c (Crossa et al. 2007 ESM Fig 1) located on fraction length 0.61-1.00L in ref b: Erayman et al. (2004). |
| o | wPt-8682: Neumann et al. (2011) 11032_2010_9411_MOESM1_ESM.doc 3.5cM proximal to wPt-5526 (ref h: Marone *et al*., 2012) |
| p | wPt-5678: mapped to gwm413 (C-0.50S ref c: ITMI group 1 maps) in Crossa et al. (2007) ESM Fig 1. |
| q | wPt-4532: Le Gouis *et al*. (2012) Maps distal to wPt1313 (0.85-1.00 ref f: CerealsDB) |
| r | wPt-5320: maps proximal to gwm337 (C-0.48S ref c: ITMI group 1 maps) in SMT Ghaffary PhD thesis (2011) (http://edepot.wur.nl/169465). |
| s | wPt-6316: maps 4cM from gwm458 (C-0.17L ref a: Sourdille et al., 2004) in NM Powell PhD thesis (2010) https://ueaeprints.uea.ac.uk/10601/1/Thesis_powell_n_2010.pdf. |
| t | gwm210, wmc243a, wmc257, wmc25b, gwm429, gwm148, barc7, wmc477, gwm55, barc128a: Gupta et al. (2008) |
| u | barc128a: Song et al. (2005) 122_2004_1871_ESM_supp3.pdf |
| v | wmc360, wPt-3632: Borràs-Gelonch et al. (2012) distal to wPt-8460 (0.89-1.00 ref f: CerealsDB) |
| w | rPt-9057: G Charmet Eucarpia presentation (2012) https://cropscience.uni-hohenheim.de/fileadmin/einrichtungen/cropscience/pdf_Dateien/Vortraege/T10.pdf Proximal to wPt-3816 (C-0.42L ref h: Marone et al. 2012). |
| x | 3B markers: Paux et al. (2008) ESM: http://www.sciencemag.org/content/suppl/2008/10/02/322.5898.101.DC1/Paux.SOM.pdf Table S6, and Killian presentation (2009) (http://ec.europa.eu/agriculture/analysis/external/basmati/diversity_arrays_technology_en.pdf) |
| y | wmc258, wmc468: both flanked in Genc et al. (2010) by wPt-4660 and wmc161, both on 0.59-0.66 in Marone et al. (2012) http://wheat.pw.usda.gov/cgi-bin/graingenes/quickquery.cgi?query=nearbyloci&arg1=Xpsp3163&arg2=10 |
| Chromosomes 4B to 6A | |
| Code letter | Source of information for locating markers to chromosome bins |
| a | Sourdille et al. (2004) |
| b | Erayman et al. (2004) and ESM chromosome groups 1-7. |
| c | ITMI chromosome group maps (groupe1v2.xls - groupe7v2.xls): http://wheat.pw.usda.gov/ggpages/SSRclub/GeneticPhysical/ |
| d | Goyal et al. (2005) s10142_2005_146_ESM_supp.pdf |
| e | Bin-mapped wheat ESTs (http://wheat.pw.usda.gov/cgi-bin/westsql/map_locus.cgi) |
| f | CerealsDB mapped DArT markers (http://www.cerealsdb.uk.net/CerealsDB/Documents/DOC_DArT_index.php) |
| g | Francki et al. (2009) 438_2008_403_MOESM1_ESM.ppt |
| h | Marone et al. (2012) |
| i | psp3163 locates on the wheat composite2004 map to cdo795 (http://wheat.pw.usda.gov/cgi-bin/graingenes/quickquery.cgi?query=nearbyloci&arg1=Xpsp3163&arg2=10) on 0.37-0.57S (ref b: Erayman et al. 2004 and ESM). |
| j | psp3030b: GrainGenes (Marker Report http://wheat.pw.usda.gov/cgi-bin/graingenes/report.cgi?class=marker&name=psp3030) |
| k | psp3103: maps close to the centromere on 4DS (Gale: http://wheat.pw.usda.gov/cgi-bin/graingenes/quickquery.cgi?query=nearbyloci&arg1=Xpsp3103-4D&arg2=10). |
| l | wPt-0431: shown mapping to the centromere in Le Gouis et al. (2012). |
| m | Several 5A DArT markers grouped around the centromere were assigned to deletion bins as follows:  -wPt-2697 mapped in Vazquez et al. poster (2009) (http://cropandsoil.oregonstate.edu/wheat/posters/Vazquez_PAG_BGRI_09.pdf) close to wPt-7769, located in RC Gaynor MSc thesis (2010) (http://ir.library.oregonstate.edu/xmlui/handle/1957/16255), flanked by gwm186 and barc56 on C-0.35L: ITMI group 5 maps (groupe5v2.xls): http://wheat.pw.usda.gov/ggpages/SSRclub/GeneticPhysical/  -wPt-797381, wPt-797382, wPt-797380, wPt-797301 and wPt-798459 also mapped in a tight cluster in SMT Ghaffary PhD thesis (2011) (http://edepot.wur.nl/169465) proximal to gwm129 (C-0.40S: ITMI group 5 maps, groupe5v2.xls). |
| n | Several group 5 RFLP markers: Linkiewicz et al. (2004) ESM available at http://wheat.pw.usda.gov/pubs/2004/Genetics/5ABD/ |
| o | barc180: Macintosh et al. (2005) Catalogue of gene symbols for wheat: 2005 supplement: barc180 shown assigned to 5AS |
| p | psb85: Khan and Shah (2011) Part of the 5AL homologous region of Ph1 on 5BL |
| q | gwm6b: Linkiewicz et al. (2004) Break point 5A-23 indicates the 4A/5A translocation segment |
| r | barc109, barc74, barc140, barc144: Song et al. (2005) 122_2004_1871_ESM_supp3.pdf |
| s | wPt-0103: Genc et al. (2010) Flanked by gwm213 and gwm371 (0.29-0.75 ref a: Sourdille et al. 2004). |
| t | gwm408: Faris et al. (2000) |
| u | barc140: RD Cuthbert PhD thesis (2011) http://mspace.lib.umanitoba.ca/bitstream/1993/4760/1/cuthbert_richard.pdf. Maps to the same position as wPt-8094 (0.79-1.00). |
| v | wPt-2856: NA Crowley PhD thesis (2010) University of Nebraska - Lincoln (http://digitalcommons.unl.edu/). Flanked by gwm190 and gdm99, both on C-0.63 (ref a: Sourdille et al. 2004) |
| w | gwm292: mapped by Snape et al. (2001) to be tightly linked with gwm212, with both distal to Vrn-D1, identified to be on 0.76-1.00L by Sarma et al. (2000). |
| y | wPt-7063: G Rosewarne presentation (2012) Maps proximal on 6AL: http://www.slideshare.net/CIMMYT/rosewarne-icrpm. |
| Chromosomes 6B to 7D | |
| Code letter | Source of information for locating markers to chromosome bins |
| a | Sourdille et al. (2004) |
| b | Erayman et al. (2004) and ESM chromosome groups 1-7. |
| c | ITMI chromosome group maps (groupe1v2.xls - groupe7v2.xls): http://wheat.pw.usda.gov/ggpages/SSRclub/GeneticPhysical/ |
| d | Goyal et al. (2005) s10142_2005_146_ESM_supp.pdf |
| e | Bin-mapped wheat ESTs (http://wheat.pw.usda.gov/cgi-bin/westsql/map_locus.cgi) |
| f | CerealsDB mapped DArT markers (http://www.cerealsdb.uk.net/CerealsDB/Documents/DOC_DArT_index.php) |
| g | Francki et al. (2009) 438_2008_403_MOESM1_ESM.ppt |
| h | Marone et al. (2012) |
| i | wmc494: distal to gwm518 (sat 0.00-1.00, ref a - Sourdille et al., 2004) in the map of RC Gaynor MSc thesis (2010): http://ir.library.oregonstate.edu/xmlui/handle/1957/16255. |
| j | psp3009: flanked in wheat composite2004 map (http://wheat.pw.usda.gov/cgi-bin/graingenes/quickquery.cgi?query=nearbyloci&arg1=Xpsp3009&arg2=10) by cdo270 (C-0.36L) and cdo507 (C-0.76S) in ref b (Erayman et al. (2004) and ESM. |
| k | wPt-6247: Maccaferri et al. (2012) Flanked by gwm518 (sat 0.00-1.00 ref a) and wPt-0554 (0.76-1.05 ref h). |
| l | wPt-665166: SMT Ghaffary PhD thesis (2011) (http://edepot.wur.nl/169465) Maps 12cM distal of gpw4440 (0.79-0.99) on the ITMI 6D map (groupe6v2.xls) - ref c. |
| m | barc175: flanked by several markers on the Synthetic x Opata map with consensus bin assignments of 0.47-0.80) http://wheat.pw.usda.gov/cgi-bin/graingenes/report.cgi?class=marker&name=BARC175. |
| n | wPt-665675, wPt-667006: SMT Ghaffary PhD thesis (2011) (http://edepot.wur.nl/169465) Flanked by cfd45 and gpw5205, both on 0.80-1.00 (ref c - ITMI 6D map). |
| o | wmc283: Rebetzke et al. (2008) Maps distal to wPt-7785 (0.59-0.89) in ref h: Marone et al. (2012). |
| p | wPt-4771: RD Cuthbert PhD thesis (2011) http://mspace.lib.umanitoba.ca/bitstream/1993/4760/1/cuthbert_richard.pdf. Flanked by wPt-7653 and wPt-7318 (0.27-1.00 ref h: Marone et al., 2012). |
| q | wPt-4230: Le Gouis et al. (2012) flanked proximally by gwm644 (C-0.33L ref a: Sourdille et al. 2004) and Akbari et al. (2006) flanked distally by wPt-5463 (C-0.33L ref g: Francki et al., 2009). |
| r | cfd31: flanked by gwm635 and gwm44 (both 0.61-1.00 ref c: ITMI 7D map) in the map of RC Gaynor MSc thesis (2010): http://ir.library.oregonstate.edu/xmlui/handle/1957/16255. |
| s | Song et al. (2005) 122_2004_1871_ESM_supp3.pdf. |
| t | psp3035: flanked by wmc405 (0.36-1.00S ref c: ITMI 7D) and cfa2174 (C-0.30L ref c: ITMI 7D) in Nanda x Wangshuibai map at http://wheat.pw.usda.gov/cgi-bin/graingenes/quickquery.cgi?query=nearbyloci&arg1=Xpsp3035.1&arg2=10. |
| u | wPt-0789: flanked by cfd21 (C-0.36S ref a) and gwm437 (C-0.30L ref a) in Excalibur x Kukri map at http://www.wheatgenome.info/cgi-bin/cmap/map_details?ref_map_set_acc=ExcKu&ref_map_accs=ExcKu_7D. |
| v | wPt-7508: 1cM from gwm473 in the map of RC Gaynor MSc thesis (2010): http://ir.library.oregonstate.edu/xmlui/handle/1957/16255. gwm473 on C-0.15S in ref d: Goyal et al. (2005) s10142_2005_146_ESM_supp.pdf. |
| w | psp3045: flanked by psr117 and psr129 (both on 0.76-0.82: ref b) by CJ Burt PhD thesis (2010) https://ueaeprints.uea.ac.uk/25796/1/2010BurtCJPhd.pdf. |
| x | psp3045: flanked in wheat composite2004 map (http://wheat.pw.usda.gov/cgi-bin/graingenes/quickquery.cgi?query=nearbyloci&arg1=Xpsp3045&arg2=10) by fbb79 and fbb325 on 0.31-0.82 (7L0.8) in ref b (Erayman et al., 2004). |
| y | wPt-744354: located distal to wPt-4441 (0.82-1.00 ref g: Francki *et al*., 2009 ESM) in composite DArT map (Killian, unpublished). |
| z | Distal to wPt-2258 (0.82-1.00 ref a) in Genc et al. (2010). |

# References

Akbari M, Wenzl P, Caig V, Carling J, Xia L, Yang S, Uszynski G, Mohler V, Lehmensiek A, Kuchel H, Hayden MJ, Howes N, Sharp P, Vaughan P, Rathmell B, Huttner E, Kilian A (2006) Diversity arrays technology (DArT) for high-throughput profiling of the hexaploid wheat genome. Theor Appl Genet 113:1409-1420

Borràs-Gelonch G, Rebetzke GJ, Richards R, and Romagosa I (2012) Genetic control of duration of pre-anthesis phases in wheat (*Triticum aestivum* L.) and relationships with leaf appearance, tillering and dry matter accumulation. J Exp Bot 63:69-89

Crossa J, Burgueño J, Dreisigacker S, Vargas M, Herrera-Foessel SA, Lillemo M, Singh RP, Trethowan R, Warburton M, Franco J, Reynolds M, Crouch JH, Ortiz R (2007) Association analysis of historical bread wheat germplasm using additive genetic covariance of relatives and population structure. Genetics 177:1889-1913

Erayman M, Sandhu D, Sidhu D, Dilbirligi M, Baenziger PS, Gill KS (2004) Demarcating the gene-rich regions of the wheat genome. Nucl Acids Res 32:3546-3565

Faris JD, Haen KM, Gill BS (2000) Saturation mapping of a gene-rich recombination hot spot region in wheat. Genetics 154:823-835

Francki MG, Walker E, Crawford AC, Broughton S, Ohm HW, Barclay I, Wilson RE, McLean R (2009) Comparison of genetic and cytogenetic maps of hexaploid wheat (*Triticum aestivum* L.) using SSR and DArT markers. Mol Genet Genomics 281:181-191

Genc Y, Oldach K, Verbyla AP, Lott G, Hassan M, Tester M, Wallwork H, McDonald GK (2010) Sodium exclusion QTL associated with improved seedling growth in bread wheat under salinity stress. Theor Appl Genet 121:877-894

Goyal A, Bandopadhyay R, Sourdille P, Endo TR, Balyan HS, Gupta PK (2005) Physical molecular maps of wheat chromosomes. Func Integr Genomics 5:260-263

Gupta PK, Balyan HS, Goyal A, Mohan A, and Kumar S (2008) An integrated physical map of 2072 SSRs Loci (gSSR and EST-SSRs) in bread wheat. Eds R Appels, R Eastwood, E Lagudah, P Langridge, M Mackay, L McIntyre, P Sharp: Proceedings 11^th^ International Wheat Genetics Symposium 2008. Sydney University Press. Pp 333-335

Le Gouis J, Bordes J, Ravel C, Heumez E, Faure S, Praud S, Galic N, Remoué C, Balfourier F, Allard V, Rousset M (2012) Genome-wide association analysis to identify chromosomal regions determining components of earliness in wheat. Theor Appl Genet 124:597-611

Linkiewicz AM, Qi LL, Gill BS, Ratnasiri A, Echalier B, Chao S, G. Lazo R, Hummel DD, Anderson OD, Akhunov ED, Dvorák J, Pathan MS, H. T. Nguyen HT, Peng JH, Lapitan NLV, Miftahudin, Gustafson JP, La Rota CM, Sorrells ME, Hossain KG, Kalavacharla V, Kianian SF, Sandhu D, Bondareva SN, Gill KS, Conley EJ, Anderson JA, Fenton RD, Close TJ, McGuire PE, Qualset CO, Dubcovsky J (2004) A 2500-locus bin map of wheat homoeologous group 5 provides insights on gene distribution and colinearity with rice. Genetics 168:665-676

Maccaferri M, Francia R, Ratti C, Rubies-Autonett C, Colalongo C, Ferrazzano G, Tuberosa R, Sanguineti MC (2012) Genetic analysis of *Soil-Borne Cereal Mosaic Virus* response in durum wheat: evidence for the role of the major quantitative trait locus *QSbm.ubo-2BS* and of minor quantitative trait loci. Mol Breeding 29:973-988

Macintosh RA, Devos KM, Dubcovsky J, Rogers WJ, Morris CF, Appels R, Anderson OD (2005) Catalogue of gene symbols for wheat: 2005 supplement. http://wheat.pw.usda.gov/ggpages/wgc/2005upd.html

Marone D, Laido` G, Gadaleta A, Colasuonno P, Ficco DBM, Giancaspro A, Giove S, Panio G, Russo MA, De Vita P, Cattivelli L, Papa R, Blanco A, Mastrangelo AM (2012) A high-density consensus map of A and B wheat genomes. Theor Appl Genet (2012) 125:1619-1638

Neumann K, Kobiljski B, Denčić S, Varshney RK, Börner A (2011) Genome-wide association mapping: a case study in bread wheat (*Triticum aestivum* L.). Mol Breeding (2011) 27:37-58

Nicholson P, Bayles R, Jennings P (2008) Understanding the basis of resistance to Fusarium head blight in UK winter wheat (REFAM) http://www.hgca.com/publications/documents/cropresearch/PR432_Final_Project_Report.pdf

Paux E, Sourdille P, Salse J, Saintenac C, Choulet F, Leroy P, Korol A, Michalak M, Kianian S, Spielmeyer W, Lagudah E, Somers D, Kilian A, Alaux M, Vautrin S, Bergès H, Eversole K, Appels R, Safar J, Simkova H, Dolezel J, Bernard M, Feuillet C (2008) A physical map of the 1-gigabase bread wheat chromosome 3B. Science 322:101-104

Rebetzke GJ, Condon AG, Farquhar GD, Appels R, Richards RA (2008) Quantitative trait loci for carbon isotope discrimination are repeatable across environments and wheat mapping populations. Theor Appl Genet 118:123-137

Sarma RN, Fish L, Gill BS, Snape JW (2000) Physical characterization of the homoeologous Group 5 chromosomes of wheat in terms of rice linkage blocks, and physical mapping of some important genes. Genome 43:191–198

Snape JW, Sarma R, Quarrie SA, Fish L, Galiba G, Sutka J (2001) Mapping genes for flowering time and frost tolerance in cereals using precise genetic stocks. Euphytica 120:309-315

Song QJ, Shi JR, Singh S, Fickus EW, Costa JM, Lewis J, Gill BS, Ward R, Cregan PB (2005) Development and mapping of microsatellite (SSR) markers in wheat. Theor Appl Genet 110:550-560

Sourdille P, Singh S, Cadalen T, Brown-Guedira GL, Gay G, Qi L, Gill BS, Dufour P, Murigneux A, Bernard M (2004) Microsatellite-based deletion bin system for the establishment of genetic-physical map relationships in wheat (*Triticum aestivum* L.). Func Int Gen 4:12-25
