# Supplementary material for: Quantitative trait loci for leaf chlorophyll fluorescence parameters, chlorophyll and carotenoid contents in relation to biomass and yield in bread wheat and their chromosome deletion bin assignments
Source: Mol Breed. 2013 Apr 10;32(1):189–210. doi: 10.1007/s11032-013-9862-8 (PMC3684715; doi:10.1007/s11032-013-9862-8)
Supplement: Supplementary file 2 — Supplementary material 2 (DOC 294 kb) [file 11032_2013_9862_MOESM2_ESM.doc]

**Quantitative trait loci for leaf chlorophyll fluorescence parameters, chlorophyll and carotenoid contents in relation to biomass and yield in bread wheat and their chromosome deletion bin assignments**

Czyczyło-Mysza I.^1^, Tyrka M.^2^, Marcińska I.^1^, Skrzypek E.^1^, Karbarz M.^3^, Dziurka M.^1^, Hura T.^1^, Dziurka K.^1^, Quarrie S.A.^4^

^1^ The *F. Górski* Institute of Plant Physiology, Polish Academy of Sciences, Kraków, Poland

^2^ Rzeszów University of Technology, Department of Biochemistry and Biotechnology, Poland.

^3^ Institute of Applied Biotechnology and Basic Sciences, University of Rzeszow

^4^ Faculty of Biology, Belgrade University, Serbia, and Visiting Professor, Newcastle University, UK.

Corresponding author: [czyczylo-mysza@wp.pl](javascript:oknoAdresat('napisz.html?to=czyczylo-mysza@wp.pl',10,10,650,540,1);)

**Supplementary Table S2.** Selected genes encoding proteins involved in photosynthetic light reactions, chlorophyll and carotenoid synthesis and metabolism, and aspects of biomass (carbohydrate) productivity and their chromosome deletion bin assignments on the Chinese Spring x SQ1 genetic map, and summary information on how they were located. Gene type indicates the role of the gene in photosynthetic light reactions (CF), chlorophyll and/or carotenoid synthesis and metabolism (Pig), and aspects of biomass (carbohydrate) productivity (Prod).

| **Chromosome** | Gene description | Gene symbol | Gene type | Clone information | **Bin location** | **Details on method of gene location** |
| --- | --- | --- | --- | --- | --- | --- |
| 1AL | Probable tocopherol cyclase (Sucrose export defective 1) | *Sxd1* | Prod | BE489692 | C-0.17 | Identified on 1A, bin assumed to be homoeologous with 1B 0.47-69L from wEST (http://wheat.pw.usda.gov/cgi-bin/westsql/map_locus.cgi) |
| 1AL | Chlorophyll a/b binding protein gene upstream region | *Cab-1* | CF | BF485346 | 0.17-0.61 | wEST (http://wheat.pw.usda.gov/cgi-bin/westsql/map_locus.cgi) |
| 1AL | Cytochrome P450 | *CytP* | Pig | OSJNBa0001O14, BF202555 | 0.17-0.61 | Comparative mapping of rice 10 to wheat 1 Chaudhary et al. (2010) and wEST (http://wheat.pw.usda.gov/cgi-bin/westsql/map_locus.cgi) |
| 1AL | Glutamyl-tRNA reductase | *GluTR* | Pig | BG312712 | 0.17-0.61 | wEST homoeologue known to be on 1A (http://wheat.pw.usda.gov/cgi-bin/westsql/map_locus.cgi) |
| 1AL | Chlorophyll a/b binding protein 2 | *Cab2* | CF | pKG1490 BE490584 | 0.61-1.00 | Maps to Xmwg706 in Dubkovsky et al. (1996) which maps slightly proximal to bcd304 on barley 1H (Langridge et al., 1995) and wEST (http://wheat.pw.usda.gov/cgi-bin/westsql/map_locus.cgi) |
| 1AL | Sucrose phosphate synthase | *Sps* | Prod | BF473773 | 0.61-1.00 | wEST (http://wheat.pw.usda.gov/cgi-bin/westsql/map_locus.cgi) |
| 1BL | Chlorophyll a/b binding protein gene upstream region | *Cab-1* | CF | BF485346 | C-0.69 | Located on 1B but bin not assigned - wEST (http://wheat.pw.usda.gov/cgi-bin/westsql/map_locus.cgi) |
| 1BL | Probable tocopherol cyclase (Sucrose export defective 1) | *Sxd1* | Prod | BE489692 | 0.47-0.69 | wEST (http://wheat.pw.usda.gov/cgi-bin/westsql/map_locus.cgi) |
| 1BL | Acetyl-coenzyme A carboxylase P-type (plastid protein) | *ACCase* | CF | BF473856 | 0.85-1.00 | wEST (http://wheat.pw.usda.gov/cgi-bin/westsql/map_locus.cgi) |
| 1DS | Probable tocopherol cyclase (Sucrose export defective 1) | *Sxd1* | Prod | BE489692 | C-0.48 | wEST (http://wheat.pw.usda.gov/cgi-bin/westsql/map_locus.cgi) |
| 1DL | Chlorophyll a/b binding protein gene upstream region | *Cab-1* | CF | BF485346 | 0.18-1.00 | wEST (http://wheat.pw.usda.gov/cgi-bin/westsql/map_locus.cgi) |
| 1DL | Cytochrome P450 | *CytP* | Pig | OSJNBa0001014, BF202555 | 0.41-1.00 | Comparative mapping of rice 10 to wheat 1 Chaudhary et al. (2010) and wEST (http://wheat.pw.usda.gov/cgi-bin/westsql/map_locus.cgi) |
| 1DL | Glutamyl-tRNA reductase | *GluTR* | Pig | BG312712 | 0.41-1.00 | wEST (http://wheat.pw.usda.gov/cgi-bin/westsql/map_locus.cgi) |
| 1DL | Chlorophyll a/b binding protein 2 | *Cab2* | CF | pKG1490 BE490584 | 0.41-1.00 | Maps to Xmwg706 in Dubkovsky et al. (1996) which maps slightly proximal to bcd304 on barley 1H (Langridge et al., 1995) and wEST (http://wheat.pw.usda.gov/cgi-bin/westsql/map_locus.cgi) |
| 2AS | Photosystem I reaction centre subunit XI (PSI-L) | *PSI-L* | CF | BE471323 | 0.78-1.00 | wEST (http://wheat.pw.usda.gov/cgi-bin/westsql/map_locus.cgi) |
| 2AS | Ribulose-1,5-bisphosphate carboxylase/oxygenase subunit binding-protein alpha subunit (CPN-60 alpha) | *CPN-60a* | Prod | BF201235 | 0.78-1.00 | wEST (http://wheat.pw.usda.gov/cgi-bin/westsql/map_locus.cgi) |
| 2AS | Acetyl-coenzyme A carboxylase | *ACCase* | CF | - | 0.78-1.00 | Location assumed through homoeology with 2BS |
| 2AS | Ribulose-1,5-bisphosphate carboxylase/oxygenase small subunit | *Rubisco-ssu* | Prod | psr109 BF292052 BE494450 | 0.78-1.00 | Mapped as codominant RFLP polymorphism between Chinese Spring and SQ1 and wEST (http://wheat.pw.usda.gov/cgi-bin/westsql/map_locus.cgi) |
| 2AS | Ribulose-1,5-bisphosphate carboxylase/oxygenase small subunit | *Rubisco-ssu* | Prod | BE496951 | C-0.78 | wEST (http://wheat.pw.usda.gov/cgi-bin/westsql/map_locus.cgi) |
| 2AS | Ferredoxin-NADP(H) oxidoreductase | *Fe-NADP(H)* | CF | BE499648 | C-0.78 | wEST (http://wheat.pw.usda.gov/cgi-bin/westsql/map_locus.cgi) |
| 2AS | Sucrose synthase type 1 | *Sus2* | Prod | BE496863 | C-0.78 | wEST (http://wheat.pw.usda.gov/cgi-bin/westsql/map_locus.cgi) |
| 2AC | Zeta-carotene desaturase | Zds | Pig | - | Cent | Rodríguez-Suárez and Atienza (2012) and comparative mapping with OsZDS located on chromosome 2 at locus R3113 (Chaudhary et al., 2010) |
| 2AL | Photosystem I reaction centre subunit psaK (PSI-K) | *PSI-K* | CF | BE495991 | C-0.85 | wEST (http://wheat.pw.usda.gov/cgi-bin/westsql/map_locus.cgi) |
| 2AL | Chlorophyll a/b binding protein CP24 10A, (CAB-10A) (LHCP) | *Cab-10A* | CF | BE638034 | C-0.85 | wEST (http://wheat.pw.usda.gov/cgi-bin/westsql/map_locus.cgi) |
| 2AL | L-ascorbate peroxidase (thylakoid lumenal 29 kDa protein) | *Asc* | CF | BE591328 | C-0.85 | wEST (http://wheat.pw.usda.gov/cgi-bin/westsql/map_locus.cgi) |
| 2AL | Beta-carotene hydroxylase 3 | *Hyd3* | Pig | - | C-0.85 | Rodríguez-Suárez and Atienza (2012) and Chaudhary et al. (2010) on rice chromosome 3 at psr151, on 2DL (Erayman et al., 2004), 2A homoeolog |
| 2AL | Polyphenol oxidase 1 | *Ppo1* | Pig | - | C-0.85 | Rodríguez-Suárez and Atienza (2012), about 35% down 2H^ch^L and comparative mapping of wheat and barley |
| 2AL | Coproporphyrinogen III oxidase | *CopIII-ox* | Pig | BE490735 | C-0.85 | wEST (http://wheat.pw.usda.gov/cgi-bin/westsql/map_locus.cgi) |
| 2BS | Ribulose-1,5-bisphosphate carboxylase/oxygenase subunit binding-protein alpha subunit (CPN-60 alpha) | *CPN-60a* | Prod | BF201235 | 0.84-1.00 | wEST (http://wheat.pw.usda.gov/cgi-bin/westsql/map_locus.cgi) |
| 2BS | Acetyl-coenzyme A carboxylase | *ACCase* | CF | BF474397 | 0.84-1.00 | wEST (http://wheat.pw.usda.gov/cgi-bin/westsql/map_locus.cgi) |
| 2BS | Ribulose-1,5-bisphosphate carboxylase/oxygenase small subunit | *Rubisco-ssu* | Prod | BF292052 BE496951 | 0.84-1.00 | Assumed homoeologue of psr109 mapped on 2AS |
| 2BS | Mg-chelatase subunit XANTHA-F (Xantha-f) | *Xantha-F* | Pig | BE422913 | 0.53-0.75 | wEST (http://wheat.pw.usda.gov/cgi-bin/westsql/map_locus.cgi) |
| 2BS | Ferredoxin-NADP(H) oxidoreductase | *Fe-NADP(H)* | CF | BE499648 | C-0.53 | wEST (http://wheat.pw.usda.gov/cgi-bin/westsql/map_locus.cgi) |
| 2BS | Sucrose synthase type 1 | *Sus2* | Pig | BE496863 | C-0.53 | Mapped very close to wmc477 by Jiang et al. (2011) and ESM, and wmc477 mapped on 2BS close to the centromere in Chinese Spring x SQ1. |
| 2BC | Zeta-carotene desaturase | *Zds* | Pig | - | Cent | Rodríguez-Suárez and Atienza (2012) and comparative mapping with OsZDS located on chromosome 2 at locus R3113 (Chaudhary et al., 2010) |
| 2BL | Geranylgeranyl pyrophosphate synthase | *Ggpps1* | Pig | BE591762 | C-0.36 | Rodríguez-Suárez and Atienza (2012) and wEST (http://wheat.pw.usda.gov/cgi-bin/westsql/map_locus.cgi) |
| 2BL | Photosystem I reaction centre subunit psaK (PSI-K) | *PSI-K* | CF | BE495991 | C-0.36 | wEST (http://wheat.pw.usda.gov/cgi-bin/westsql/map_locus.cgi) |
| 2BL | L-ascorbate peroxidase (thylakoid lumenal 29 kDa protein) | *Asc* | CF | BE591328 | C-0.36 | wEST (http://wheat.pw.usda.gov/cgi-bin/westsql/map_locus.cgi) |
| 2BL | Beta-carotene hydroxylase 3 | *Hyd3* | Pig | - | 0.35-0.36 | Rodríguez-Suárez and Atienza (2012) and Chaudhary et al. (2010) on rice chromosome 3 at psr151, on 2DL (Erayman et al., 2004), 2B homoeolog |
| 2BL | Polyphenol oxidase 1 | *Ppo1* | Pig | - | C-0.89 | Rodríguez-Suárez and Atienza (2012), about 35% down 2H^ch^L and comparative mapping of wheat and barley |
| 2BL | Chlorophyll a/b binding protein CP24 10A, (CAB-10A) (LHCP) | *Cab-10A* | CF | BE638034 | 0.36-0.50 | wEST (http://wheat.pw.usda.gov/cgi-bin/westsql/map_locus.cgi) |
| 2BL | Coproporphyrinogen III oxidase | *CopIII-ox* | Pig | BE490735 | 0.50-0.89 | wEST (http://wheat.pw.usda.gov/cgi-bin/westsql/map_locus.cgi) |
| 2DS | Ribulose-1,5-bisphosphate carboxylase/oxygenase subunit binding-protein alpha subunit (CPN-60 alpha) | *CPN-60a* | Prod | BF201235 | 0.47-1.00 | wEST (http://wheat.pw.usda.gov/cgi-bin/westsql/map_locus.cgi) |
| 2DS | Ribulose-1,5-bisphosphate carboxylase/oxygenase small subunit | *Rubisco-ssu* | Prod | BE496951 | 0.47-1.00 | wEST (http://wheat.pw.usda.gov/cgi-bin/westsql/map_locus.cgi) |
| 2DS | Ferredoxin-NADP(H) oxidoreductase | *Fe-NADP(H)* | CF | BE499648 | 0.47-1.00 | wEST (http://wheat.pw.usda.gov/cgi-bin/westsql/map_locus.cgi) |
| 2DS | Acetyl-coenzyme A carboxylase | *ACCase* | CF | - | 0.47-1.00 | Location assumed through homoeology with 2BS |
| 2DS | Sucrose synthase type 1 | *Sus2* | Prod | BE496863 | 0.33-0.47 | wEST (http://wheat.pw.usda.gov/cgi-bin/westsql/map_locus.cgi) |
| 2DS | Geranylgeranyl pyrophosphate synthase | *Ggpps1* | Pig | BE591762 | 0.33-0.47 | Rodríguez-Suárez and Atienza (2012) and wEST (http://wheat.pw.usda.gov/cgi-bin/westsql/map_locus.cgi) |
| 2DC | Zeta-carotene desaturase | *Zds* | Pig | - | Cent | Rodríguez-Suárez and Atienza (2012) and comparative mapping with OsZDS located on chromosome 2 at locus R3113 (Chaudhary et al., 2010) |
| 2DL | Photosystem I reaction centre subunit psaK (PSI-K) | *PSI-K* | CF | BE495991 | C-0.49 | wEST (http://wheat.pw.usda.gov/cgi-bin/westsql/map_locus.cgi) |
| 2DL | Photosystem II 10 kDa polypeptide | *PSII-10kDa* | CF | BG313573 | C-0.49 | wEST (http://wheat.pw.usda.gov/cgi-bin/westsql/map_locus.cgi) |
| 2DL | L-ascorbate peroxidase (thylakoid lumenal 29 kDa protein) | *Asc* | CF | BE591328 | C-0.49 | wEST (http://wheat.pw.usda.gov/cgi-bin/westsql/map_locus.cgi) |
| 2DL | Polyphenol oxidase 1 | *Ppo1* | Pig | - | C-0.49 | Rodríguez-Suárez and Atienza (2012), about 35% down 2H^ch^L and comparative mapping of wheat and barley |
| 2DL | Beta-carotene hydroxylase 3 | *Hyd3* | Pig | - | 0.47-0.49 | Rodríguez-Suárez and Atienza (2012) and Chaudhary et al. (2010) on rice chromosome 3 at psr151, on 2DL (Erayman et al., 2004) |
| 2DL | Coproporphyrinogen III oxidase | *CopIII-ox* | Pig | BE490735 | 0.76-1.00 | wEST (http://wheat.pw.usda.gov/cgi-bin/westsql/map_locus.cgi) |
| 3AS | 1-deoxy-D-xylulose 5-phosphate reductoisomerase | Dxr | Pig | BE591845 | 0.45-1.00 | Distal on 3H^ch^S in Rodríguez-Suárez and Atienza (2012) and wEST (http://wheat.pw.usda.gov/cgi-bin/westsql/map_locus.cgi) |
| 3AS | Sucrose-phosphate synthase | *Sps* | Prod | BG274132 | 0.45-1.00 | wEST (http://wheat.pw.usda.gov/cgi-bin/westsql/map_locus.cgi) |
| 3AL | Lycopene epsilon cyclase | *e-Lcy* | Pig | - | C-0.42 | Near 3H^ch^L centromere (Rodríguez-Suárez and Atienza, 2012) and comparative mapping of barley with wheat |
| 3AL | Ribulose-1,5-bisphosphate carboxylase/oxygenase small subunit | *Rubisco-ssu* | Prod | BE494450 | 0.78-1.00 | wEST (http://wheat.pw.usda.gov/cgi-bin/westsql/map_locus.cgi) |
| 3AL | Sucrose-phosphate synthase | *Sps* | Prod | BG263365 | 0.78-1.00 | wEST (http://wheat.pw.usda.gov/cgi-bin/westsql/map_locus.cgi) |
| 3BS | 1-deoxy-D-xylulose 5-phosphate reductoisomerase | Dxr | Pig | BE591845 | 0.78-1.00 | Distal on 3H^ch^S in Rodríguez-Suárez and Atienza (2012) and wEST (http://wheat.pw.usda.gov/cgi-bin/westsql/map_locus.cgi) |
| 3BS | Sucrose-phosphate synthase | *Sps* | Prod | BG274134 | 0.57-0.78 | wEST (http://wheat.pw.usda.gov/cgi-bin/westsql/map_locus.cgi) |
| 3BL | Lycopene epsilon cyclase | *e-Lcy* | Pig | - | C-0.22 | Near 3H^ch^L centromere (Rodríguez-Suárez and Atienza, 2012) and comparative mapping of barley with wheat |
| 3BL | Photosystem II protein W-like protein | *PSII-W* | CF | BG313553 | 0.50-0.63 | wEST (http://wheat.pw.usda.gov/cgi-bin/westsql/map_locus.cgi) |
| 3BL | Ribulose-1,5-bisphosphate carboxylase/oxygenase small subunit | *Rubisco-ssu* | Prod | BE494450 | 0.63-1.00 | wEST (http://wheat.pw.usda.gov/cgi-bin/westsql/map_locus.cgi) |
| 3BL | Sucrose-phosphate synthase | *Sps* | Prod | BG263365 | 0.63-1.00 | wEST (http://wheat.pw.usda.gov/cgi-bin/westsql/map_locus.cgi) |
| 3DS | 1-deoxy-D-xylulose 5-phosphate reductoisomerase | Dxr | Pig | BE591845 | 0.55-1.00 | Distal on 3H^ch^S in Rodríguez-Suárez and Atienza (2012) and wEST (http://wheat.pw.usda.gov/cgi-bin/westsql/map_locus.cgi) |
| 3DL | Lycopene epsilon cyclase | *e-Lcy* | Pig | - | C-0.27 | Near 3H^ch^L centromere (Rodríguez-Suárez and Atienza, 2012) and comparative mapping of barley with wheat |
| 3DL | Ribulose-1,5-bisphosphate carboxylase/oxygenase small subunit | *Rubisco-ssu* | Prod | BE494450 | 0.27-0.81 | wEST (http://wheat.pw.usda.gov/cgi-bin/westsql/map_locus.cgi) |
| 3DL | Sucrose-phosphate synthase | *Sps* | Prod | BG263365 | 0.27-0.81 | wEST (http://wheat.pw.usda.gov/cgi-bin/westsql/map_locus.cgi) |
| 4AL | Plastocyanin chloroplast precursor | Plc | CF | psr160 | 0.80-1.00 | Mapped as codominant RFLP (psr160.1) polymorphism between CS and SQ1 |
| 4AL | Sucrose synthase 2 | *Sus* | Prod | psr490 BE498428 | 0.80-1.00 | Mapped as codominant RFLP (psr490.2) polymorphism between CS and SQ1, and wEST (http://wheat.pw.usda.gov/cgi-bin/westsql/map_locus.cgi) |
| 4AL | Phosphoribulosekinase | *Prk* | Prod | psr115 | 0.66-0.80 | Mapped as codominant RFLP (psr115) polymorphism between CS and SQ1 |
| 4AL | Acetyl-coenzyme A carboxylase P-type (plastid protein) | *ACCase* | CF | BF473856 | 0.66-0.80 | wEST (http://wheat.pw.usda.gov/cgi-bin/westsql/map_locus.cgi) |
| 4AL | Rubisco subunit binding-protein alpha subunit | *Rubisco-sbp-a* | Prod | BF483796 | 0.59-0.66 | wEST (http://wheat.pw.usda.gov/cgi-bin/westsql/map_locus.cgi) |
| 4AL | Chlorophyll a/b binding protein CP29 precursor | *Cab(CP29)* | CF | BE497446 | 0.43-0.59 | wEST (http://wheat.pw.usda.gov/cgi-bin/westsql/map_locus.cgi) |
| 4AL | Ribulose 1,5-bisphosphate carboxylase activase (RcaA and RcaB) | *RcaA/B* | Prod | BE497324 | C-0.43 | wEST (http://wheat.pw.usda.gov/cgi-bin/westsql/map_locus.cgi) |
| 4AS | Porphobilinogen deaminase | *Pbd* | Pig | BE490825 | C-0.63 | wEST (http://wheat.pw.usda.gov/cgi-bin/westsql/map_locus.cgi) |
| 4AS | Sucrose synthase | *Sus* | Prod | BG263213 | 0.20-0.63 | wEST (http://wheat.pw.usda.gov/cgi-bin/westsql/map_locus.cgi) |
| 4AS | Sucrose transporter 1 | *Sut1* | Prod | - | 0.63-0.71 | Comparative mapping with psr59, psr104 in rye (Börner and Korzun, 1998; Korzun et al., 2001), Erayman et al. (2004) and GrainGenes (http://wheat.pw.usda.gov/GG2/index.shtml) |
| 4AS | Oxygen-evolving complex 25.6 kD protein | *O-ec 25.6kDa* | CF | BE426317 | 0.63-0.76 | wEST (http://wheat.pw.usda.gov/cgi-bin/westsql/map_locus.cgi) |
| 4AS | Phytoene desaturase (dehydrogenase) | *Pds* | Pig | BE591172 | 0.63-0.76 | Rodríguez-Suárez and Atienza (2012) and wEST (http://wheat.pw.usda.gov/cgi-bin/westsql/map_locus.cgi) |
| 4AS | Uroporphyrinogen III synthase | *UpoIIIs* | Pig | BF485078 | 0.76-1.00 | wEST (http://wheat.pw.usda.gov/cgi-bin/westsql/map_locus.cgi) |
| 4BS | Chlorophyll a/b-binding protein CP29 precursor | *Cab(CP29)* | CF | BE497446 | 0.57-0.81 | wEST (http://wheat.pw.usda.gov/cgi-bin/westsql/map_locus.cgi) |
| 4BS | Ribulose 1,5-bisphosphate carboxylase activase (RcaA and RcaB) | *RcaA/B* | Prod | BE497324 | 0.37-0.57 | wEST (http://wheat.pw.usda.gov/cgi-bin/westsql/map_locus.cgi) |
| 4BS | 4-hydroxy-3-methylbut-2-enyl diphosphate reductase [ispH] | *Hdr* | Pig | - | C-0.57 | Rodríguez-Suárez and Atienza (2012), about 25% up 4H^ch^S and comparative mapping of wheat and barley |
| 4BL | Sucrose synthase | *Sus* | Prod | BG263213 | C-0.71 | wEST (http://wheat.pw.usda.gov/cgi-bin/westsql/map_locus.cgi) |
| 4BL | Oxygen-evolving complex 25.6 kD protein | *O-ec 25.6kDa* | CF | BE426317 | C-0.71 | wEST (http://wheat.pw.usda.gov/cgi-bin/westsql/map_locus.cgi) |
| 4BL | Sucrose transporter 1 | *Sut1* | Prod | - | C-0.71 | Comparative mapping with psr59, psr104 in rye (Börner and Korzun, 1998; Korzun et al., 2001), Erayman et al. (2004) and GrainGenes (http://wheat.pw.usda.gov/GG2/index.shtml) |
| 4BL | Phytoene desaturase (dehydrogenase) | *Pds* | Pig | BE591172 | 0.86-1.00 | Rodríguez-Suárez and Atienza (2012) and wEST (http://wheat.pw.usda.gov/cgi-bin/westsql/map_locus.cgi) |
| 4BL | Uroporphyrinogen III synthase | *UpoIIIs* | Pig | BF485078 | 0.86-1.00 | wEST (http://wheat.pw.usda.gov/cgi-bin/westsql/map_locus.cgi) |
| 4BL | Cytochrome P450 | *CytP* | Pig | - | 0.86-1.00 | Comparative mapping of rice 3 to wheat 4 (Chaudhary et al., 2010) and mapped as codominant SSR (dupw43) polymorphism between CS and SQ1 |
| 4DS | Ribulose 1,5-bisphosphate carboxylase activase (RcaA and RcaB) | *RcaA/B* | Prod | BE497324 BE489218 | C-0.53 | wEST (http://wheat.pw.usda.gov/cgi-bin/westsql/map_locus.cgi) |
| 4DS | 4-hydroxy-3-methylbut-2-enyl diphosphate reductase [ispH] | *Hdr* | Pig | - | C-0.53 | Rodríguez-Suárez and Atienza (2012), about 25% up 4H^ch^S and comparative mapping of wheat and barley |
| 4DL | Porphobilinogen deaminase | *Pbd* | Pig | BE490825 | C-0.31 | wEST (http://wheat.pw.usda.gov/cgi-bin/westsql/map_locus.cgi) |
| 4DL | Oxygen-evolving complex 25.6 kD protein | *O-ec 25.6kDa* | CF | BE426317 | 0.31-0.56 | wEST (http://wheat.pw.usda.gov/cgi-bin/westsql/map_locus.cgi) |
| 4DL | Photosystem II 10K protein | *PSII-10kDa* | CF | BF201352 | C-0.31 | wEST (http://wheat.pw.usda.gov/cgi-bin/westsql/map_locus.cgi) |
| 4DL | Sucrose transporter 1 | *Sut1* | Prod | - | 0.31-0.56 | Comparative mapping with psr59, psr104 in rye (Börner and Korzun, 1998; Korzun et al., 2001), Erayman et al. (2004) and GrainGenes (http://wheat.pw.usda.gov/GG2/index.shtml) |
| 4DL | Phytoene desaturase (dehydrogenase) | *Pds* | Pig | BE591172 | 0.56-1.00 | Rodríguez-Suárez and Atienza (2012) and wEST (http://wheat.pw.usda.gov/cgi-bin/westsql/map_locus.cgi) |
| 4DL | Cytochrome P450 | *CytP* | Pig | - | 0.86-1.00 | Comparative mapping of rice 3 to wheat 4 Chaudhary et al. (2010) with dupw43 and Erayman ey al. (2004) |
| 4DL | Uroporphyrinogen III synthase | *UpoIIIs* | Pig | BF485078 | 0.71-1.00 | wEST (http://wheat.pw.usda.gov/cgi-bin/westsql/map_locus.cgi) |
| 5AL | Ribulose-1,5-bisphosphate carboxylase/oxygenase small subunit | *Rubisco-ssu* | Prod | BE425296 | C-0.57 | wEST (http://wheat.pw.usda.gov/cgi-bin/westsql/map_locus.cgi) |
| 5AL | Porphobilinogen deaminase | *Pbd* | Pig | BE490825 | C-0.57 | wEST (http://wheat.pw.usda.gov/cgi-bin/westsql/map_locus.cgi) |
| 5AL | Chlorophyll a/b-binding protein precursor (Lhca4) | *Lhca4* | CF | BE590997 | 0.35-0.57 | wEST (http://wheat.pw.usda.gov/cgi-bin/westsql/map_locus.cgi) |
| 5AL | Ferrochelatase II (Protoheme ferro-lyase) (Heme synthetase) | *Phe-Fel* | Pig | BF484133 | 0.35-0.57 | wEST (http://wheat.pw.usda.gov/cgi-bin/westsql/map_locus.cgi) |
| 5AL | Photosystem I F subunit precursor | *PSI-F* | CF | BE496891 | 0.57-0.78 | wEST (http://wheat.pw.usda.gov/cgi-bin/westsql/map_locus.cgi) |
| 5AL | Staygreen (rice sgr homolog) | *Sgr* | Pig | - | 0.57-0.78 | Assumed homoeolog of Sgr on wheat 5B (Erayman et al., 2004) |
| 5AL | Phytoene synthase 3 | *Psy3-A* | Pig | OSJNBa0043I04 | 0.67 | Rodríguez-Suárez and Atienza (2012) on 5H^ch^L, comparative mapping to rice 9 (Chaudhary et al., 2010) next to marker wg1026 on 5AL 0.67 (Gill et al., 1996), and bin-mapped by Dibari et al. (2012) |
| 5AL | Chlorophyll a/b binding protein | *Cab1.2* | CF | *pKG1490* | 0.67-0.68 | Maps to 5A^m^ in Dubkovsky et al. (1996) slightly proximal of psr120.1, and Erayman et al. (2004) and ESM. |
| 5AL | Chlorophyll a/b binding protein | *Cab1.1* | CF | *pKG1490* | 0.67-0.82 | Maps to 5A^m^ in Dubkovsky et al. (1996) slightly proximal of psr2021.1(dhn2), and Erayman et al. (2004) and ESM. |
| 5AL | Magnesium-chelatase subunit chlD (Mg-protoporphyrin IX chelatase) | *Mg-PpoIXc* | Pig | BE500894 | 0.76-1.00 | wEST 5D homoeolog on 5A (http://wheat.pw.usda.gov/cgi-bin/westsql/map_locus.cgi) |
| 5AL | Sucrose-phosphate synthase | *Sps* | Prod | BE499840 | 0.87-1.00 | wEST (http://wheat.pw.usda.gov/cgi-bin/westsql/map_locus.cgi) |
| 5BS | Carotenoid cleavage dioxygenase 1 | Ccd1 | Pig | - | 0.81-1.00 | The most distal marker on 5H^ch^S in Rodríguez-Suárez and Atienza (2012) |
| 5BL | Ferrochelatase II (Protoheme ferro-lyase) (Heme synthetase) | *Phe-Fel* | Pig | BF484133 | C-0.29 | wEST (http://wheat.pw.usda.gov/cgi-bin/westsql/map_locus.cgi) |
| 5BL | Ribulose-1,5-bisphosphate carboxylase/oxygenase small subunit | *Rubisco-ssu* | Prod | BE425296 | C-0.75 | wEST (http://wheat.pw.usda.gov/cgi-bin/westsql/map_locus.cgi) |
| 5BL | Chlorophyll a/b-binding protein precursor (Lhca4) | *Lhca4* | CF | BE590997 | 0.35-0.57 | wEST (http://wheat.pw.usda.gov/cgi-bin/westsql/map_locus.cgi) |
| 5BL | Phytoene synthase 3 | *Psy3-B* | Pig | OSJNBa0043I04 | 0.75 | Rodríguez-Suárez and Atienza (2012) on 5H^ch^L, comparative mapping to rice 9 (Chaudhary et al., 2010) next to marker wg1026 on 5BL 0.75 (Gill et al., 1996), and bin-mapped by Dibari et al. (2012) |
| 5BL | Staygreen (rice sgr homolog) | *Sgr* | Pig | - | 0.76-0.79 | Mapped on rice 9 to abc155 (Park et al., 2007), also on wheat 5B (Erayman et al., 2004) |
| 5BL | Rubisco subunit binding-protein alpha subunit | *Rubisco-sbp-a* | Prod | BF483796 | 0.76-0.79 | wEST (http://wheat.pw.usda.gov/cgi-bin/westsql/map_locus.cgi) |
| 5BL | Sucrose transporter 2 | *Sut2* | Prod | BE403785 | 0.76-0.79 | wEST (http://wheat.pw.usda.gov/cgi-bin/westsql/map_locus.cgi) |
| 5BL | Chlorophyll a/b-binding protein Wcab precursor | *Wcab* | CF | BE490584 | 0.79-1.00 | wEST (http://wheat.pw.usda.gov/cgi-bin/westsql/map_locus.cgi) |
| 5BL | Photosystem II protein W-like protein | *PSII-W* | CF | BG313553 | 0.79-1,00 | wEST (http://wheat.pw.usda.gov/cgi-bin/westsql/map_locus.cgi) |
| 5BL | Photosystem I F subunit precursor | *PSI-F* | CF | BE496891 | 0.79-1.00 | wEST (http://wheat.pw.usda.gov/cgi-bin/westsql/map_locus.cgi) |
| 5BL | Rieske iron-sulfur protein | *RISP* |  | BF483151 | 0.79-1.00 | wEST (http://wheat.pw.usda.gov/cgi-bin/westsql/map_locus.cgi) |
| 5DS | Carotenoid cleavage dioxygenase 1 | Ccd1 | Pig | - | 0.81-1.00 | The most distal marker on 5H^ch^S in Rodríguez-Suárez and Atienza (2012) |
| 5DS | Sucrose transporter 2 | *Sut2* | Prod | BE403785 | 0.81-1.00 | wEST (http://wheat.pw.usda.gov/cgi-bin/westsql/map_locus.cgi) |
| 5DS | Ribulose-1,5-bisphosphate carboxylase/oxygenase small subunit | *Rubisco-ssu* | Prod | BE496951 | C-0.63 | wEST (http://wheat.pw.usda.gov/cgi-bin/westsql/map_locus.cgi) |
| 5DL | Ribulose-1,5-bisphosphate carboxylase/oxygenase small subunit | *Rubisco-ssu* | Prod | BE494450 BE425296 | C-0.60 | wEST (http://wheat.pw.usda.gov/cgi-bin/westsql/map_locus.cgi) |
| 5DL | Chlorophyll a/b-binding protein precursor (Lhca4) | *Lhca4* | CF | BE590997 | C-0.60 | wEST (http://wheat.pw.usda.gov/cgi-bin/westsql/map_locus.cgi) |
| 5DL | Ferrochelatase II (Protoheme ferro-lyase) (Heme synthetase) | *Phe-Fel* | Pig | BF484133 | C-0.60 | wEST (http://wheat.pw.usda.gov/cgi-bin/westsql/map_locus.cgi) |
| 5DL | Phytoene synthase 3 | *Psy3-D* | Pig | OSJNBa0043I04 | 0.75 | Rodríguez-Suárez and Atienza (2012) on 5H^ch^L, comparative mapping to rice 9 (Chaudhary et al., 2010) next to marker wg1026 on 5DL 0.75 (Gill et al., 1996), and bin-mapped by Dibari et al. (2012) |
| 5DL | Staygreen (rice sgr homolog) | *Sgr* | Pig | - | 0.76-0.79 | Assumed homoeolog of Sgr on wheat 5B (Erayman et al., 2004) |
| 5DL | Rubisco subunit binding-protein alpha subunit | *Rubisco-sbp-a* | Prod | BF483796 | 0.76-1.00 | wEST (http://wheat.pw.usda.gov/cgi-bin/westsql/map_locus.cgi) |
| 5DL | Magnesium-chelatase subunit chlD (Mg-protoporphyrin IX chelatase) | *Mg-PpoIXc* | Pig | BE500894 | 0.76-1.00 | wEST (http://wheat.pw.usda.gov/cgi-bin/westsql/map_locus.cgi) |
| 5DL | Photosystem II protein W-like protein | *PSII-W* | CF | BE490222 | 0.76-1.00 | wEST (http://wheat.pw.usda.gov/cgi-bin/westsql/map_locus.cgi) |
| 5DL | Photosystem I F subunit precursor | *PSI-F* | CF | BE496891 | 0.76-1.00 | wEST (http://wheat.pw.usda.gov/cgi-bin/westsql/map_locus.cgi) |
| 6AS | Chlorophyll a/b-binding protein Wcab precursor | *Wcab* | CF | BE490584 | 0.65-1.00 | wEST (http://wheat.pw.usda.gov/cgi-bin/westsql/map_locus.cgi) |
| 6AS | Ferredoxin-NADP(H) oxidoreductase | *Fe-NADP(H)* | CF | BE496826 | 0.65-1.00 | wEST (http://wheat.pw.usda.gov/cgi-bin/westsql/map_locus.cgi) |
| 6AS | Rieske iron-sulfur protein | *RISP* | CF | T09557 | C-0.35 | Gao et al. (2004) adjacent to fba85 on ITMI chromosome 6A map (groupe6v2.xls): http://wheat.pw.usda.gov/ggpages/SSRclub/GeneticPhysical/ |
| 6AL | Lycopene beta cyclase | *b-Lcy* | Pig | - | C-0.55 | Rodríguez-Suárez and Atienza (2012) on 6H^ch^L, comparative mapping to rice 9 (Chaudhary et al., 2010) close to marker psr113 on 6BL 0.36-0.44 (Erayman et al., 2004) |
| 6AL | Protoporphyrinogen oxidase (Ppo I) (protoporphyrinogen IX oxidase isozyme I) (Ppx I) | *Ppo1(Ppx1)* | Pig | BF473368 | 0.55-0.90 | wEST (http://wheat.pw.usda.gov/cgi-bin/westsql/map_locus.cgi) |
| 6BS | Ferredoxin-NADP(H) oxidoreductase | *Fe-NADP(H)* | CF | BE496826 | sat0.00-1.00 | wEST (http://wheat.pw.usda.gov/cgi-bin/westsql/map_locus.cgi) |
| 6BS | Chlorophyll a/b-binding protein Wcab precursor | *Wcab* | CF | BE490584 | 0.76-1.05 | wEST (http://wheat.pw.usda.gov/cgi-bin/westsql/map_locus.cgi) |
| 6BL | Lycopene beta cyclase | *b-Lcy* | Pig | - | 0.36-0.44 | Rodríguez-Suárez and Atienza (2012) on 6H^ch^L, comparative mapping to rice 9 (Chaudhary et al., 2010) close to marker psr113 on 6BL 0.36-0.44 (Erayman et al., 2004) |
| 6BL | Photosystem II 10 kDa polypeptide | *PSII-10kDa* | CF | BE497576 | 0.36-0.40 | wEST (http://wheat.pw.usda.gov/cgi-bin/westsql/map_locus.cgi) |
| 6BL | Photosystem II 10 kDa polypeptide | *PSII-10kDa* | CF | BG313573 | 0.40-1.00 | wEST (http://wheat.pw.usda.gov/cgi-bin/westsql/map_locus.cgi) |
| 6BL | Porphobilinogen deaminase | *Pbd* | Pig | BF201435 | 0.40-1.00 | wEST (http://wheat.pw.usda.gov/cgi-bin/westsql/map_locus.cgi) |
| 6DS | Ferredoxin-NADP(H) oxidoreductase | *Fe-NADP(H)* | CF | BE496826 | 0.99-1.00 | wEST (http://wheat.pw.usda.gov/cgi-bin/westsql/map_locus.cgi) |
| 6DS | Chlorophyll a/b-binding protein Wcab precursor | *Wcab* | CF | BE490584 | 0.45-0.79 | wEST (http://wheat.pw.usda.gov/cgi-bin/westsql/map_locus.cgi) |
| 6DL | Lycopene beta cyclase | *b-Lcy* | Pig | - | 0.29-0.47 | Rodríguez-Suárez and Atienza (2012) on 6H^ch^L, comparative mapping to rice 9 (Chaudhary et al., 2010) close to marker psr113 on 6BL 0.36-0.44 (Erayman et al., 2004) |
| 6DL | Photosystem II 10 kDa polypeptide | *PSII-10kDa* | CF | BE497576 | C-0.29 | wEST (http://wheat.pw.usda.gov/cgi-bin/westsql/map_locus.cgi) |
| 6DL | Photosystem II 10 kDa polypeptide | *PSII-10kDa* | CF | BG313573 | 0.80-1.00 | wEST (http://wheat.pw.usda.gov/cgi-bin/westsql/map_locus.cgi) |
| 7AS | Plastocyanin chloroplast precursor | Plc | CF | - | 0.89-1.00 | psr160 located on 0.89-1.00 according to Erayman et al. (2004) and ESM. |
| 7AS | Sucrose synthase 2 | *Sus* | Prod | psr490 BE498428 | 0.89-1.00 | Mapped as codominant RFLP (psr490.3) polymorphism between CS and SQ1, and wEST (http://wheat.pw.usda.gov/cgi-bin/westsql/map_locus.cgi) |
| 7AS | Glutamyl-tRNA reductase (1st isoform) | *GluTR* | Pig | BE515566 | 0.59-0.89 | wEST (http://wheat.pw.usda.gov/cgi-bin/westsql/map_locus.cgi) |
| 7AS | Sucrose synthase type 1 | *Sus* | Prod | BF474552 | 0.59-0.89 | wEST (http://wheat.pw.usda.gov/cgi-bin/westsql/map_locus.cgi) |
| 7AL | Protoporphyrin IX magnesium chelatase subunit (Xantha-h) | *Xantha-h* | Pig | BE488496 | 0.39-0.71 | wEST (http://wheat.pw.usda.gov/cgi-bin/westsql/map_locus.cgi) |
| 7AL | Delta-aminolevulinic acid dehydratase (porphobilinogen synthase) (ALADH) | *Aladh* | Pig | BE497239 | 0.39-0.71 | wEST (http://wheat.pw.usda.gov/cgi-bin/westsql/map_locus.cgi) |
| 7AL | Phytoene synthase (Psy1-A1) | *Psy1-A1* | Pig | FJ234424 AK070716 | 0.90-1.00 | Singh et al. (2009) mapped distal to wmc273 on 7DL and on the same BAC (OSJNBa0069C14) located to 7DL 0.82-1.00 (Leonard et al., 2008) as rice PSY1 on chromosome 6 (Chaudhary et al., 2010) |
| 7BS | Sucrose synthase type 1 | *Sus* | Prod | BF474552 | 0.27-1.00 | wEST (http://wheat.pw.usda.gov/cgi-bin/westsql/map_locus.cgi) |
| 7BL | Protoporphyrin IX magnesium chelatase subunit (Xantha-h) | *Xantha-h* | Pig | BE488496 | 0.78-1.00 | wEST (http://wheat.pw.usda.gov/cgi-bin/westsql/map_locus.cgi) |
| 7BL | Phytoene synthase (Psy1-A1) | *Psy1-B1* | Pig | FJ234424 AK070716 | 0.78-1.00 | Singh et al. (2009) mapped distal to wmc273 on 7DL and on the same BAC (OSJNBa0069C14) located to 7DL 0.82-1.00 (Leonard et al., 2008) as rice PSY1 on chromosome 6 (Chaudhary et al., 2010) |
| 7DS | Plastocyanin chloroplast precursor | Plc | CF | psr160 | 0.61-1.00 | Mapped as codominant RFLP (psr160.2) polymorphism between CS and SQ1 |
| 7DS | Sucrose synthase 2 | *Sus* | Prod | psr490 BE498428 | 0.61-1.00 | Mapped as codominant RFLP (psr490.3) polymorphism between CS and SQ1, and wEST (http://wheat.pw.usda.gov/cgi-bin/westsql/map_locus.cgi) |
| 7DL | Protoporphyrin IX magnesium chelatase subunit (Xantha-h) | *Xantha-h* | Pig | BE488496 | 0.61-0.82 | wEST (http://wheat.pw.usda.gov/cgi-bin/westsql/map_locus.cgi) |
| 7DL | Phytoene synthase (Psy1-A1) | *Psy1-D1* | Pig | FJ234424 AK070716 | 0.82-1.00 | Singh et al. (2009) mapped distal to wmc273 on 7DL and on the same BAC (OSJNBa0069C14) located to 7DL 0.82-1.00 (Leonard et al., 2008) as rice PSY1 on chromosome 6 (Chaudhary et al., 2010) |

References

Börner A, Korzun V (1998) A consensus linkage map of rye (*Secale cereale* L.) including 374 RFLPs, 24 isozymes and 15 gene loci. Theor Appl Genet 97: 1279-1288.

Chaudhary N Nijhawan A, Khurana JP, Khurana P (2010) Carotenoid biosynthesis genes in rice: structural analysis, genome-wide expression profiling and phylogenetic analysis. Mol Genet Genom 283:13-33

Dibari B, Murat F, Chosson A, Gautier V, Poncet C, Lecomte P, Mercier I, Bergès H, Pont C, Blanco A, Salse J (2012) Deciphering the genomic structure, function and evolution of carotenogenesis related phytoene synthases in grasses. BMC Genomics 13:221-235

Dubcovsky J, Luo M-C, Zhon G-Y, Bransteitter R, Desai A, Killian A, Kleinhofs A, Dvorak J (1996) Genetic map of diploid wheat, *Triticum monococcum* L., and its comparison with maps of *Hordeum vulgare* L. Genetics 143:983-999

Erayman M, Sandhu D, Sidhu D, Dilbirligi M, Baenziger PS, Gill KS (2004) Demarcating the gene-rich regions of the wheat genome. Nucl Acids Res 32:3546-3565

Gao LF, Jing RL, Huo NX, Li Y, Li XP, Zhou RH, Chang XP, Tang JF, Ma ZY, Jia JZ (2004) One hundred and one new microsatellite loci derived from ESTs (EST-SSRs) in bread wheat. Theor Appl Genet 108:1392-1400

Gill KS, Gill BS, Endo TR, Boyko EV (1996) Identification and high-density mapping of gene-rich regions in chromosome group 5 of wheat. Genetics 143:1001-1012

Jiang Q, Hou J, Hao C, Wang L, Ge H, Dong Y, Zhang X (2011) The wheat (*T. aestivum*) sucrose synthase 2 gene (*TaSus2*) active in endosperm development is associated with yield traits. Func Integr Genomics 11:49-61

Korzun V, Malyshev S, Voylokov AV, Börner A (2001) A genetic map of rye (*Secale cereale* L.) combining RFLP, isozyme, protein, microsatellite and gene loci. Theor Appl Genet 102:709–717

Langridge P, Karakousis A, Collins N, Kretchmer J, Manning S (1995) A consensus linkage map of barley. Mol Breeding 1:389-395

Leonard JVM, Watson CJW, Carter AH, Hansen JL, Zemetra RS, Santra DK, Campbell KG, Riera-Lizarazu O (2008) Identification of a candidate gene for the wheat endopeptidase *Ep-D1* locus and two other STS markers linked to the eyespot resistance gene *Pch1*. Theor Appl Genet (2008) 116:261-270

Park S-Y, Yu J-W, Park J-S, Li J, Yoo S-C, Lee N-Y, Lee S-K, Jeong S-W, Seo HS, Koh H-J, Jeon J-S, Park Y-I, Paek N-C (2007) The senescence-induced staygreen protein regulates chlorophyll degradation. Plant Cell 19:1649-1664

Rodríguez-Suárez C, Atienza SG (2012) *Hordeum chilense* genome, a useful tool to investigate the endosperm yellow pigment content in the Triticeae. BMC Plant Biol 12:200 (http://www.biomedcentral.com/1471-2229/12/200)

Singh A, Reimer S, Pozniak CJ, Clarke FR, Clarke JM, Knox RE, Singh AK (2009) Allelic variation at *Psy1-A1* and association with yellow pigment in durum wheat grain. Theor Appl Genet 118:1539–1548
