# Supplementary material for: Quantitative trait loci for leaf chlorophyll fluorescence parameters, chlorophyll and carotenoid contents in relation to biomass and yield in bread wheat and their chromosome deletion bin assignments
Source: Mol Breed. 2013 Apr 10;32(1):189–210. doi: 10.1007/s11032-013-9862-8 (PMC3684715; doi:10.1007/s11032-013-9862-8)
Supplement: Supplementary file 3 — Supplementary material 3 (DOC 115 kb) [file 11032_2013_9862_MOESM3_ESM.doc]

**Quantitative trait loci for leaf chlorophyll fluorescence parameters, chlorophyll and carotenoid contents in relation to biomass and yield in bread wheat and their chromosome deletion bin assignments**

Czyczyło-Mysza I.^1^, Tyrka M.^2^, Marcińska I.^1^, Skrzypek E.^1^, Karbarz M.^3^, Dziurka M.^1^, Hura T.^1^, Dziurka K.^1^, Quarrie S.A.^4^

^1^ The *F. Górski* Institute of Plant Physiology, Polish Academy of Sciences, Kraków, Poland

^2^ Rzeszów University of Technology, Department of Biochemistry and Biotechnology, Poland.

^3^ Institute of Applied Biotechnology and Basic Sciences, University of Rzeszow

^4^ Faculty of Biology, Belgrade University, Serbia, and Visiting Professor, Newcastle University, UK.

Corresponding author: [czyczylo-mysza@wp.pl](javascript:oknoAdresat('napisz.html?to=czyczylo-mysza@wp.pl',10,10,650,540,1);)

**Supplementary Table S3**. Phenotypic performance for traits related to chlorophyll *a* fluorescence parameters, chlorophyll contents, SPAD readings, carotenoid contents, dry weight per plant, grain weight per ear and grain yield per plant of doubled haploid lines and their parents during four experiments. Ratios of parent means and DH maxima and minima are also given. Max and min are maximum and minimum DH line mean data for each trait.

|  | **Experiment** | **Parents** | | | **CSDH population** | | | | | | |
| --- | --- | --- | --- | --- | --- | --- | --- | --- | --- | --- | --- |
| **Traits** |  | **CS** | **SQ** | **CS/ SQ** | **Mean** | **SD** | **min** | **max** | **max/min** | **skewness** | **kurtosis** |
| **F_v_/F_m_** | 2007 | 0.822 | 0.830 | 0.99 | 0.827 | 0.007 | 0.805 | 0.839 | 1.04 | -0.743 | 0.255 |
|  | 2008 | 0.824 | 0.811 | 1.02 | 0.821 | 0.007 | 0.800 | 0.836 | 1.05 | -0.259 | 0.128 |
|  | 2010 | 0.806 | 0.809 | 1.00 | 0.817 | 0.009 | 0.787 | 0.839 | 1.07 | -0.230 | 0.226 |
|  | 2011 | 0.827 | 0.798 | 1.04 | 0.826 | 0.010 | 0.790 | 0.842 | 1.07 | -1.216 | 1.976 |
|  | **mean** | **0.820** | **0.812** | **1.01** | **0.823** | **0.008** | **0.796** | **0.839** | **1.05** | **-0.612** | **0.646** |
| **PI** | 2007 | 3.65 | 3.89 | 0.94 | 3.58 | 0.66 | 1.43 | 4.61 | 3.22 | -0.867 | 0.880 |
|  | 2008 | 2.80 | 2.04 | 1.37 | 2.38 | 0.42 | 1.14 | 3.39 | 2.97 | -0.051 | 0.613 |
|  | 2010 | 1.44 | 1.24 | 1.16 | 1.74 | 0.32 | 1.02 | 2.70 | 2.65 | 0.745 | 0.814 |
|  | 2011 | 4.59 | 3.45 | 1.33 | 4.51 | 0.74 | 2.47 | 5.82 | 2.36 | -0.266 | -0.420 |
|  | **mean** | **3.12** | **2.65** | **1.20** | **3.05** | **0.53** | **1.51** | **4.13** | **2.80** | **-0.110** | **0.472** |
| **ABS/CS_m_** | 2007 | 3581.3 | 3780.3 | 0.95 | 3684.1 | 169.6 | 3236.0 | 4010.0 | 1.24 | -0.392 | 0.172 |
|  | 2008 | 3758.7 | 3357.7 | 1.12 | 3589.9 | 135.3 | 3211.0 | 3867.0 | 1.20 | -0.080 | -0.224 |
|  | 2010 | 2843.7 | 2922.3 | 0.97 | 2930.3 | 166.7 | 2309.0 | 3193.5 | 1.38 | -0.904 | 1.299 |
|  | 2011 | 2060.0 | 1770.0 | 1.16 | 2022.6 | 93.7 | 1775.3 | 2324.7 | 1.31 | 0.525 | 1.444 |
|  | **mean** | **3060.9** | **2957.6** | **1.05** | **3056.7** | **141.3** | **2632.8** | **3348.8** | **1.28** | **-0.213** | **0.673** |
| **TR_0_/CS_m_** | 2007 | 2945.3 | 3136.0 | 0.94 | 3047.6 | 164.7 | 2606.0 | 3364.5 | 1.29 | -0.438 | 0.090 |
|  | 2008 | 3095.3 | 2724.0 | 1.14 | 2947.4 | 133.7 | 2568.0 | 3224.0 | 1.26 | -0.065 | -0.211 |
|  | 2010 | 2302.0 | 2354.7 | 0.98 | 2394.0 | 150.9 | 1858.7 | 2670.0 | 1.44 | -0.769 | 0.787 |
|  | 2011 | 1705.0 | 1416.0 | 1.20 | 1671.5 | 91.7 | 1415.7 | 1940.0 | 1.37 | 0.166 | 0.928 |
|  | **mean** | **2511.9** | **2407.7** | **1.06** | **2515.0** | **135.3** | **2112.1** | **2799.6** | **1.34** | **-0.276** | **0.399** |
| **ET_0_/CS_m_** | 2007 | 1872.7 | 1995.7 | 0.94 | 1902.1 | 179.4 | 1273.5 | 2199.0 | 1.73 | -1.008 | 1.507 |
|  | 2008 | 1824.7 | 1493.0 | 1.22 | 1658.4 | 136.7 | 1243.5 | 1952.0 | 1.57 | -0.415 | 0.669 |
|  | 2010 | 1124.0 | 1133.0 | 0.99 | 1260.1 | 120.4 | 894.7 | 1533.7 | 1.71 | -0.529 | 0.384 |
|  | 2011 | 1110.3 | 883.0 | 1.26 | 1087.3 | 81.4 | 853.0 | 1308.3 | 1.53 | -0.014 | 0.784 |
|  | **mean** | **1482.9** | **1376.2** | **1.10** | **1477.0** | **129.5** | **1066.2** | **1748.3** | **1.64** | **-0.491** | **0.836** |
| **DI_0_/CS_m_** | 2007 | 636.0 | 644.3 | 0.99 | 636.8 | 12.1 | 614.5 | 673.5 | 1.10 | 0.793 | 0.405 |
|  | 2008 | 663.3 | 633.7 | 1.05 | 642.5 | 9.3 | 617.0 | 667.7 | 1.08 | 0.338 | 0.386 |
|  | 2010 | 541.7 | 567.7 | 0.95 | 536.3 | 29.8 | 445.7 | 590.3 | 1.32 | -0.733 | 0.603 |
|  | 2011 | 355.0 | 354.0 | 1.00 | 351.1 | 13.4 | 325.0 | 390.7 | 1.20 | 0.808 | 0.800 |
|  | **mean** | **549.0** | **549.9** | **1.00** | **541.7** | **16.1** | **500.5** | **580.5** | **1.18** | **0.301** | **0.549** |
| **RC/CS_m_** | 2007 | 1606.6 | 1724.8 | 0.93 | 1635.6 | 144.7 | 1187.9 | 1953.0 | 1.64 | -0.853 | 1.277 |
|  | 2008 | 1558.0 | 1299.6 | 1.20 | 1428.2 | 120.4 | 1071.1 | 1703.5 | 1.59 | -0.172 | 0.629 |
|  | 2010 | 1008.3 | 936.5 | 1.08 | 1004.0 | 83.8 | 731.2 | 1185.0 | 1.62 | -0.564 | 0.941 |
|  | 2011 | 1044.9 | 892.1 | 1.17 | 1008.7 | 75.9 | 809.1 | 1212.6 | 1.50 | -0.165 | 0.190 |
|  | **mean** | **1304.4** | **1213.3** | **1.09** | **1269.1** | **106.2** | **949.8** | **1513.5** | **1.59** | **-0.438** | **0.759** |
| **Chl_a+b_** | 2007 | 13.4 | 15.7 | 0.85 | 13.4 | 2.1 | 6.9 | 19.9 | 2.88 | -0.500 | -0.070 |
|  | 2008 | 4.8 | 5.0 | 0.96 | 4.4 | 0.6 | 2.2 | 7.0 | 3.18 | 0.453 | 1.402 |
|  | 2010 | 5.4 | 8.1 | 0.67 | 7.0 | 1.4 | 3.2 | 10.9 | 3.41 | -0.100 | -0.510 |
|  | 2011 | - | - | - | - | - | - | - | - | - | - |
|  | **mean** | **7.87** | **9.60** | **0.83** | **8.27** | **1.4** | **4.1** | **12.6** | **3.16** | **-0.049** | **0.274** |
| **SPAD** | 2007 | - | - |  | - | - | - | - | - | - | - |
|  | 2008 | - | - |  | - | - | - | - | - | - | - |
|  | 2010 | 31.8 | 46.1 | 0.69 | 37.5 | 3.4 | 25.5 | 50.6 | 1.98 | -0.160 | 0.820 |
|  | 2011 | 48.4 | 43.9 | 1.10 | 45.5 | 2.7 | 31.8 | 57.8 | 1.82 | -0.517 | 2.383 |
|  | **mean** | **40.1** | **45.0** | **0.90** | **41.5** | **3.05** | **28.7** | **54.2** | **1.90** | **-0.339** | **1.601** |
| **Car** | 2007 | 1.6 | 2.2 | 0.73 | 1.7 | 0.3 | 0.8 | 2.8 | 3.50 | -0.295 | -0.109 |
|  | 2008 | 0.4 | 0.5 | 0.80 | 0.3 | 0.1 | 0.2 | 0.7 | 3.50 | 0.458 | 0.051 |
|  | 2010 | 1.4 | 1.9 | 0.74 | 1.4 | 0.3 | 0.7 | 2.1 | 3.00 | -0.175 | -0.830 |
|  | 2011 | - | - | - | - | - | - | - | - | - | - |
|  | **mean** | **1.13** | **1.53** | **0.75** | **1.13** | **0.23** | **0.57** | **1.87** | **3.33** | **-0.004** | **-0.296** |
| **DWP** | 2007 | 8.58 | 5.00 | 1.72 | 7.34 | 1.55 | 3.60 | 10.38 | 2.88 | -0.302 | -0.595 |
|  | 2008 | 5.53 | 4.48 | 1.23 | 4.63 | 1.07 | 1.97 | 7.35 | 3.73 | -0.124 | -0.312 |
|  | 2010 | 7.17 | 4.95 | 1.45 | 5.94 | 1.11 | 3.56 | 8.81 | 2.47 | -0.225 | 0.023 |
|  | 2011 | - | - |  | - | - | - | - | - | - | - |
|  | **mean** | **7.09** | **4.81** | **1.47** | **6.03** | **1.52** | **3.04** | **8.85** | **3.03** | **-0.175** | **-0.260** |
| **GWE** | 2007 | 1.020 | 0.897 | 1.14 | 0.96 | 0.29 | 0.40 | 1.74 | 4.35 | 0.388 | -0.121 |
|  | 2008 | 0.981 | 0.662 | 1.48 | 0.89 | 0.25 | 0.33 | 1.48 | 4.48 | 0.068 | -0.442 |
|  | 2010 | 0.996 | 1.175 | 0.85 | 1.05 | 0.24 | 0.44 | 1.71 | 3.89 | 0.232 | 0.215 |
|  | 2011 | - | - |  | - | - | - | - | - | - | - |
|  | **mean** | **0.999** | **0.911** | **1.10** | **0.97** | **0.26** | **0.39** | **1.64** | **4.25** | **0.229** | **-0.116** |
| **YP** | 2007 | 4.320 | 2.542 | 1.70 | 3.57 | 0.87 | 1.38 | 5.29 | 3.83 | -0.276 | -0.278 |
|  | 2008 | 2.435 | 2.590 | 0.94 | 2.18 | 0.63 | 0.75 | 4.19 | 5.59 | 0.253 | 0.159 |
|  | 2010 | 3.382 | 2.800 | 1.21 | 3.04 | 0.61 | 1.42 | 4.44 | 3.13 | 0.069 | -0.561 |
|  | 2011 | - | - | - | - | - | - | - | - | - | - |
|  | **mean** | **3.379** | **2.644** | **1.28** | **2.93** | **0.70** | **1.18** | **4.64** | **4.18** | **0.015** | **-0.227** |
