# Supplementary material for: Quantitative trait loci for leaf chlorophyll fluorescence parameters, chlorophyll and carotenoid contents in relation to biomass and yield in bread wheat and their chromosome deletion bin assignments
Source: Mol Breed. 2013 Apr 10;32(1):189–210. doi: 10.1007/s11032-013-9862-8 (PMC3684715; doi:10.1007/s11032-013-9862-8)
Supplement: Supplementary file 4 — Supplementary material 4 (DOCX 63 kb) [file 11032_2013_9862_MOESM4_ESM.docx]

**Quantitative trait loci for leaf chlorophyll fluorescence parameters, chlorophyll and carotenoid contents in relation to biomass and yield in bread wheat and their chromosome deletion bin assignments**

Czyczyło-Mysza I.^1^, Tyrka M.^2^, Marcińska I.^1^, Skrzypek E.^1^, Karbarz M.^3^, Dziurka M.^1^, Hura T.^1^, Dziurka K.^1^, Quarrie S.A.^4^

^1^ The *F. Górski* Institute of Plant Physiology, Polish Academy of Sciences, Kraków, Poland

^2^Rzeszów University of Technology, Department of Biochemistry and Biotechnology, Poland.

^3^ Institute of Applied Biotechnology and Basic Sciences, University of Rzeszow

^4^ Faculty of Biology, Belgrade University, Serbia, and Visiting Professor, Newcastle University, UK.

Corresponding author: [czyczylo-mysza@wp.pl](javascript:oknoAdresat('napisz.html?to=czyczylo-mysza%40wp.pl',10,10,650,540,1);)

**Table S4.** Main characteristics of QTLs controlling Fv/Fm, ABS/CSm, TRo/CSm, ETo/CSm, DIo/CSm, RC/CSm, chlorophyll and carotenoid contents, SPAD reading and productivity traits detected in 2007, 2008, 2010 and 2011 (EI, EII, EIII and EIV, respectively) in the doubled haploid lines (CSDH) identified using CIM. * QTLs significant at 1000 permutations. **QTLs also identified as signficant by SMA are shown in bold text.**Where several QTLs for the same trait were present on a chromosome, they are numbered with a suffix according to increasing distance from the top marker.§ Although a QTL peak was clearly present, the software did not recognise it to provide R^2^ and additive data.

| **Traits** | **Chrom group** | **Genome** | **QTL** | **Experiment** | **Marker** | **Position (cM)** | **LOD max.** | **R^2^ (%)** | **Additive** |
| --- | --- | --- | --- | --- | --- | --- | --- | --- | --- |
| **F_v_/F_m_** | **2** | **A** | ***QF_v_/F_m_.csdh-2A**** | **2011** | **gwm339** | **106.1** | **3.6** | **12.0** | **0.003** |
|  | **3** | **A** | ***QF_v_/F_m_.csdh-3A*** | **2011** | **wPt-9928** | **4.5** | **2.9** | **9.6** | **-0.003** |
|  | **6** | **A** | ***QF_v_/F_m_.csdh-6A**** | **2008** | **csb112(*Dhn5*)** | **97.2** | **4.1** | **13.8** | **0.003** |
|  | 7 | A | *QF_v_/F_m_.csdh-7A.1* | 2010 | wmc283a | 77.2 | 2.8 | 8.7 | -0.003 |
|  | **7** | **A** | ***QF_v_/F_m_.csdh-7A.2**** | **2010** | **barc108a** | **128.3** | **4.0** | **12.8** | **0.003** |
|  | **2** | **B** | ***QF_v_/F_m_.csdh-2B*** | **2008** | **m65p64.5** | **161.8** | **2.4** | **7.8** | **0.002** |
|  | **5** | **B** | ***QF_v_/F_m_.csdh-5B*** | **2010** | **wPt-1548** | **99.0** | **4.6** | **§** | **-ve** |
|  | **6** | **B** | ***QF_v_/F_m_.csdh-6B.1*** | **2007** | **wPt-2424** | **71.0** | **2.8** | **9.0** | **-0.002** |
|  | **6** | **B** | ***QF_v_/F_m_.csdh-6B.2*** | **2007** | **gwm191** | **79.2** | **2.9** | **9.6** | **-0.002** |
|  | **1** | **D** | ***QF_v_/F_m_.csdh-1D*** | **2011** | **m59p78.0** | **188.9** | **3.3** | **§** | **+ve** |
|  | **2** | **D** | ***QF_v_/F_m_.csdh-2D.1*** | **2008** | **wPt-7466** | **143.2** | **2.5** | **8.1** | **-0.002** |
|  | 2 | D | *QF_v_/F_m_.csdh-2D.2* | 2007 | cfd73 | 175.0 | 3.2 | 11.1 | 0.003 |
|  | **2** | **D** | ***QF_v_/F_m_.csdh-2D.3**** | **2007** | **gwm349** | **200.4** | **5.3** | **17.7** | **-0.004** |
| **PI** | **2** | **A** | ***QPI.csdh-2A**** | **2007** | **psr575.1** | **110.7** | **3.4** | **9.4** | **0.206** |
|  | **3** | **A** | ***QPI.csdh-3A.1**** | **2008** | **cfa2234** | **65.5** | **4.6** | **12.8** | **-0.143** |
|  | **3** | **A** | ***QPI.csdh-3A.2*** | **2008** | **wPt-734079** | **109.5** | **2.1** | **5.3** | **0.103** |
|  | **4** | **A** | ***QPI.csdh-4A**** | **2008** | **gwm30b** | **125.7** | **3.9** | **11.2** | **0.135** |
|  | **5** | **A** | ***QPI.csdh-5A**** | **2010** | **psp3003b** | **112.8** | **3.8** | **13.3** | **-0.124** |
|  | **7** | **A** | ***QPI.csdh-7A**** | **2007** | **wPt-9641** | **2.0** | **3.9** | **11.0** | **0.238** |
|  | 2 | B | *QPI.csdh-2B.1* | 2008 | psr331.2 | 134.0 | 2.8 | 7.3 | 0.110 |
|  | **2** | **B** | ***QPI.csdh-2B.2*** | **2008** | **wPt-667945** | **152.0** | **2.2** | **7.0** | **0.100** |
|  | **4** | **B** | ***QPI.csdh-4B.1**** | **2011** | ***Rht-B1*** | **65.2** | **4.9** | **18.4** | **-0.318** |
|  | 4 | B | *QPI.csdh-4B.2* | 2007 | barc60 | 98.4 | 2.5 | 6.7 | -0.183 |
|  | **4** | **B** | ***QPI.csdh-4B.3*** | **2010** | **wmc47** | **110.9** | **2.5** | **8.9** | **-0.099** |
|  | **5** | **B** | ***QPI.csdh-5B**** | **2008** | **wPt-5346** | **30.6** | **3.8** | **10.3** | **0.128** |
|  | **6** | **B** | ***QPI.csdh-6B.1**** | **2007** | **wPt-2424** | **71.7** | **3.9** | **11.4** | **-0.231** |
|  | **6** | **B** | ***QPI.csdh-6B.2**** | **2007** | **gwm191** | **79.2** | **3.9** | **§** | **-ve** |
|  | **6** | **B** | ***QPI.csdh-6B.3*** | **2008** | **wPt-0406** | **163.6** | **5.5** | **§** | **+ve** |
|  | **2** | **D** | ***QPI.csdh-2D.1*** | **2010** | **gwm539** | **129.5** | **2.5** | **8.2** | **0.099** |
|  | **2** | **D** | ***QPI.csdh-2D.2*** | **2007** | **wPt-6574** | **159.2** | **3.0** | **8.2** | **0.196** |
|  | **4** | **D** | ***QPI.csdh-4D**** | **2007** | **wPt-5809** | **34.0** | **5.6** | **24.6** | **0.383** |
|  | **5** | **D** | ***QPI.csdh-5D.1*** | **2007** | **m77p64.8** | **157.0** | **2.1** | **§** | **+ve** |
|  | **5** | **D** | ***QPI.csdh-5D.2*** | **2010** | **gwm212** | **171.1** | **2.1** | **7.5** | **-0.092** |
|  | **5** | **D** | ***QPI.csdh-5D.3*** | **2008** | **m92p78.9** | **242.9** | **2.0** | **6.8** | **-0.104** |
|  | **6** | **D** | ***QPI.csdh-6D*** | **2007** | **wPt-665675** | **144.6** | **2.6** | **11.0** | **0.230** |
|  | 7 | D | *QPI.csdh-7D** | 2007 | psp3094b | 168.1 | 3.4 | 10.5 | -0.223 |
| **ABS/CS_m_** | **1** | **A** | ***QABS.csdh-1A.1*** | **2011** | **m51p65.5** | **20.2** | **2.8** | **10.7** | **31.150** |
|  | **1** | **A** | ***QABS.csdh-1A.2**** | **2008** | **wPt-731617** | **42.6** | **3.4** | **10.9** | **45.486** |
|  | **1** | **A** | ***QABS.csdh-1A.3*** | **2007** | **m77p64.13** | **43.7** | **3.1** | **9.0** | **53.897** |
|  | **2** | **A** | ***QABS.csdh-2A*** | **2010** | **wPt-729945** | **180.8** | **2.4** | **7.8** | **48.017** |
|  | **2** | **B** | ***QABS.csdh-2B**** | **2008** | **wPt-8776** | **155.5** | **3.4** | **12.2** | **47.847** |
|  | 3 | B | *QABS.csdh-3B* | 2010 | gwm285 | 97.8 | 2.2 | 7.2 | -47.455 |
|  | **5** | **B** | ***QABS.csdh-5B.1*** | **2008** | **wPt-3085** | **29.5** | **2.9** | **9.3** | **41.712** |
|  | **5** | **B** | ***QABS.csdh-5B.2**** | **2011** | **psp3037** | **78.7** | **3.7** | **13.0** | **-34.188** |
|  | **5** | **B** | ***QABS.csdh-5B.3*** | **2010** | **wPt-1548** | **99.0** | **3.3** | **10.9** | **-58.606** |
|  | **5** | **B** | ***QABS.csdh-5B.4*** | **2010** | **m65p64.8a** | **113.5** | **2.1** | **9.6** | **76.247** |
|  | **6** | **B** | ***QABS.csdh-6B.1**** | **2007** | **wPt-2424** | **71.7** | **4.5** | **14.3** | **-66.706** |
|  | **6** | **B** | ***QABS.csdh-6B.2**** | **2007** | **wg232.4** | **81.9** | **4.6** | **§** | **-ve** |
|  | **6** | **B** | ***QABS.csdh-6B.3**** | **2007** | **wPt-2564** | **87.5** | **4.3** | **13.1** | **-63.898** |
|  | 1 | D | *QABS.csdh-1D* | 2007 | rPt-4471 | 70.0 | 2.2 | 6.2 | 44.279 |
|  | **2** | **D** | ***QABS.csdh-2D*** | **2007** | **wPt-9848** | **200.4** | **3.1** | **9.2** | **-54.558** |
|  | **3** | **D** | ***QABS.csdh-3D*** | **2010** | **wPt-732092** | **71.1** | **3.1** | **10.5** | **-56.458** |
|  | 5 | D | *QABS.csdh-5D* | 2011 | wPt-4295 | 28.7 | 2.6 | 8.2 | -43.350 |
|  | **6** | **D** | ***QABS.csdh-6D*** | **2007** | **wPt-665675** | **160.4** | **2.1** | **5.8** | **43.300** |
| **TR_o_/CS_m_** | **1** | **A** | ***QTR_o_.csdh-1A.1**** | **2008** | **wPt-731617** | **42.6** | **3.6** | **11.6** | **46.671** |
|  | **1** | **A** | ***QTR_o_.csdh-1A.2*** | **2007** | **m77p64.13** | **43.7** | **3.1** | **9.0** | **52.134** |
|  | **2** | **A** | ***QTR_o_.csdh-2A*** | **2011** | **gwm339** | **106.1** | **2.2** | **7.6** | **26.041** |
|  | **4** | **A** | ***QTR_o_.csdh-4A*** | **2008** | **gwm30b** | **120.7** | **2.2** | **8.3** | **40.334** |
|  | **1** | **B** | ***QTR_o_.csdh-1B*** | **2010** | **wPt-3451** | **90.3** | **2.4** | **8.0** | **44.266** |
|  | **2** | **B** | ***QTR_o_.csdh-2B**** | **2008** | **m65p64.5** | **161.8** | **3.6** | **12.1** | **48.367** |
|  | **5** | **B** | ***QTR_o_.csdh-5B.1*** | **2008** | **wPt-3085** | **29.5** | **2.5** | **8.0** | **38.160** |
|  | **5** | **B** | ***QTR_o_.csdh-5B.2*** | **2011** | **psp3037** | **76.6** | **3.2** | **12.2** | **-32.770** |
|  | **5** | **B** | ***QTR_o_.csdh-5B.3**** | **2010** | **psr806.2** | **86.9** | **4.0** | **16.0** | **-63.389** |
|  | **5** | **B** | ***QTR_o_.csdh-5B.4**** | **2010** | **wPt-1548** | **99.0** | **4.3** | **15.0** | **-59.813** |
|  | **6** | **B** | ***QTR_o_.csdh-6B.1*** | **2007** | **wPt-2424** | **71.7** | **4.5** | **§** | **-ve** |
|  | **6** | **B** | ***QTR_o_.csdh-6B.2*** | **2007** | **wg232.4** | **81.9** | **4.7** | **§** | **-ve** |
|  | **6** | **B** | ***QTR_o_.csdh-6B.3**** | **2007** | **wPt-2564** | **87.5** | **4.3** | **13.1** | **-62.010** |
|  | 1 | D | *QTR_o_.csdh-1D* | 2007 | rPt-4471 | 70.0 | 2.1 | 5.9 | 41.758 |
|  | **2** | **D** | ***QTR_o_.csdh-2D.1*** | **2008** | **wPt-7825** | **193.4** | **2.6** | **9.9** | **-42.806** |
|  | **2** | **D** | ***QTR_o_.csdh-2D.2*** | **2007** | **gwm349** | **200.4** | **3.2** | **9.4** | **-53.693** |
| **ET_o_/CS_m_** | **2** | **A** | ***QET_o_.csdh-2A**** | **2007** | **gwm339** | **106.2** | **3.4** | **9.1** | **55.905** |
|  | **3** | **A** | ***QET_o_.csdh-3A.1*** | **2008** | **rPt-9057** | **66.8** | **3.3** | **9.5** | **-40.56** |
|  | 3 | A | *QET_o_.csdh-3A.2* | 2008 | wPt-3133 | 116.2 | 2.5 | 7.1 | 39.83 |
|  | **4** | **A** | ***QET_o_.csdh-4A**** | **2008** | **gwm30b** | **124.7** | **3.5** | **11.0** | **44.336** |
|  | 6 | A | *QET_o._csdh-6A** | 2007 | wPt-667844 | 48.5 | 4.0 | 10.9 | 63.691 |
|  | **7** | **A** | ***QET_o._csdh-7A.1*** | **2007** | **wPt-9641** | **4.0** | **2.9** | **8.3** | **56.125** |
|  | **7** | **A** | ***QET_o._csdh-7A.2*** | **2007** | **m39p78.4** | **16.9** | **2.2** | **§** | **+ve** |
|  | **1** | **B** | ***QET_o._csdh-1B*** | **2008** | **wPt-3852** | **55.0** | **2.2** | **5.7** | **31.943** |
|  | **2** | **B** | ***QET_o._csdh-2B*** | **2008** | **wPt-8776** | **164.8** | **2.9** | **8.5** | **39.507** |
|  | 4 | B | *QET_o._csdh-4B* | 2007 | psp3163 | 57.1 | 2.0 | 4.8 | -40.816 |
|  | **5** | **B** | ***QET_o._csdh-5B.1**** | **2008** | **wPt-5346** | **30.6** | **3.6** | **10.6** | **43.207** |
|  | **5** | **B** | ***QET_o._csdh-5B.2*** | **2010** | **wPt-1548** | **99.0** | **2.7** | **9.5** | **-38.069** |
|  | **6** | **B** | ***QET_o._csdh-6B.1**** | **2007** | **wPt-2424** | **71.7** | **6.5** | **20.5** | **-88.35** |
|  | **6** | **B** | ***QET_o._csdh-6B.2**** | **2007** | **wg232.4** | **81.9** | **7.4** | **21.7** | **-92.383** |
|  | **6** | **B** | ***QET_o._csdh-6B.3**** | **2007** | **wPt-2564** | **87.5** | **6.8** | **20.2** | **-88.947** |
|  | **6** | **B** | ***QET_o._csdh-6B.4**** | **2008** | **wPt-0406** | **163.6** | **2.9** | **§** | **+ve** |
|  | **4** | **D** | ***QET_o_.csdh-4D**** | **2007** | **wPt-5809** | **35.0** | **6.0** | **23.8** | **103.099** |
|  | **6** | **D** | ***QET_o_.csdh-6D*** | **2007** | **m69p78.10** | **146.6** | **2.7** | **9.8** | **58.733** |
|  | 7 | D | *QET_o_.csdh-7D.1** | 2007 | wPt-744354 | 179.4 | 4.8 | 14.3 | -72.997 |
|  | 7 | D | *QET_o_.csdh-7D.2** | 2007 | gwm37 | 190.9 | 3.5 | 10.0 | -61.157 |
| **DI_o_/CS_m_** | **5** | **A** | ***QDI_o_.csdh-5A*** | **2007** | ***Vrn-A1*** | **130.6** | **3.2** | **10.9** | **-4.095** |
|  | **7** | **A** | ***QDI_o_.csdh-7A*** | **2011** | **psr3094a** | 190.2 | 2.0 | 7.4 | -3.667 |
|  | **1** | **B** | ***QDI_o._csdh-1B*** | **2011** | **wPt-0044** | **2.3** | **2.5** | **9.7** | **4.380** |
|  | **5** | **B** | ***QDI_o._csdh-5B.1**** | **2007** | **wPt-5346** | **36.6** | **3.4** | **12.3** | **4.454** |
|  | **5** | **B** | ***QDI_o._csdh-5B.2**** | **2008** | **wmc73** | 42.8 | 4.3 | 15.0 | 4.738 |
|  | **5** | **B** | ***QDI_o._csdh-5B.3*** | **2007** | **wPt-5737** | **47.3** | **2.9** | **10.3** | **4.050** |
|  | 5 | B | *QDI_o._csdh-5B.4* | 2008 | psr725 | 69.8 | 2.7 | 9.6 | -3.673 |
|  | **1** | **D** | ***QDI_o_.csdh-1D.1*** | **2007** | **wPt-730783** | **0.0** | **2.4** | **7.9** | **-3.488** |
|  | 1 | D | *QDI_o_.csdh-1D.2* | 2010 | wPt-664824 | 51.6 | 2.8 | 8.5 | -10.979 |
|  | **1** | **D** | ***QDI_o_.csdh-1D.3**** | **2010** | **wPt-4671** | **77.4** | **5.3** | **18.5** | **16.102** |
|  | **3** | **D** | ***QDI_o_.csdh-3D**** | **2010** | **wPt-732092** | **72.2** | **3.3** | **10.7** | **-10.183** |
|  | **5** | **D** | ***QDI_o_.csdh-5D*** | **2008** | **m62p64.9a** | **198.9** | **2.1** | **7.3** | **2.561** |
|  | **7** | **D** | ***QDI_o_.csdh-7D.1**** | **2010** | **wPt-744354** | **183.4** | **4.6** | **15.1** | **-19.859** |
|  | 7 | D | *QDI_o_.csdh-7D.2* | 2010 | wPt-2054 | 195.4 | 2.5 | 7.4 | 14.338 |
| **RC/CS_m_** | **3** | **A** | ***QRC.csdh-3A**** | **2008** | **cfa2234** | **64.5** | **5.4** | **14.4** | **-43.679** |
|  | **4** | **A** | ***QRC.csdh-4A*** | **2008** | **gwm30b** | **126.4** | **3.0** | **7.8** | **32.434** |
|  | **5** | **A** | ***QRC.csdh-5A**** | **2007** | ***Vrn-A1*** | **130.6** | **3.3** | **10.1** | **-48.847** |
|  | **6** | **A** | ***QRC.csdh-6A**** | **2011** | **wPt-8124** | **141.8** | **3.3** | **10.1** | **-25.139** |
|  | 2 | B | *QRC.csdh-2B.1* | 2011 | wPt-4613 | 4.5 | 3.0 | 8.5 | -22.083 |
|  | **2** | **B** | ***QRC.csdh-2B.2*** | **2008** | **psr331.2** | **133.5** | **3.0** | **7.7** | **32.343** |
|  | **4** | **B** | ***QRC.csdh-4B**** | **2011** | ***Rht-B1*** | **65.2** | **6.6** | **20.7** | **-34.364** |
|  | **5** | **B** | ***QRC.csdh-5B**** | **2008** | **wPt-5346** | **30.6** | **5.6** | **15.2** | **44.912** |
|  | **6** | **B** | ***QRC.csdh-6B.1*** | **2010** | **wPt-2564** | **87.5** | **2.8** | **9.2** | **-26.920** |
|  | **6** | **B** | ***QRC.csdh-6B.2*** | **2008** | **wPt-0406** | **163.6** | **5.0** | **§** | **+ve** |
|  | **2** | **D** | ***QRC.csdh-2D**** | **2007** | **gwm349** | **200.4** | **3.4** | **10.4** | **-49.447** |
|  | **3** | **D** | ***QRC.csdh-3D.1*** | **2010** | **cfd35** | **2.0** | **3.1** | **10.3** | **-29.581** |
|  | 3 | D | *QRC.csdh-3D.2* | 2011 | wPt-669482 | 115.3 | 2.6 | 7.4 | -21.445 |
|  | **4** | **D** | ***QRC.csdh-4D*** | **2007** | **wPt-5809** | **40.3** | **2.3** | **6.7** | **45.221** |
|  | **5** | **D** | ***QRC.csdh-5D*** | **2007** | **gwm212** | **172.6** | **2.1** | **6.1** | **36.615** |
|  | **7** | **D** | ***QRC.csdh-7D**** | **2010** | **barc154** | **71.2** | **5.1** | **17.6** | **37.181** |
| **chl_a+b_** | **3** | **A** | ***Qchl_a+b_.csdh-3A.1*** | **2008** | **psr345.2** | **0.0** | **2.7** | **8.8** | **-0.241** |
|  | **3** | **A** | ***Qchl_a+b_.csdh-3A.2*** | **2007** | **wPt-732716** | **100.4** | **2.3** | **8.2** | **0.783** |
|  | **5** | **A** | ***Qchl_a+b_.csdh-5A.1**** | **2010** | **wPt-8794** | **119.5** | **3.2** | **9.2** | **-0.546** |
|  | **5** | **A** | ***Qchl_a+b_.csdh-5A.2*** | **2008** | **wPt-8794** | **120.5** | **3.0** | **9.9** | **-0.260** |
|  | **3** | **B** | ***Qchl_a+b_.csdh-3B**** | **2008** | **wPt-1682** | **27.4** | **3.6** | **11.5** | **0.286** |
|  | **4** | **B** | ***Qchl_a+b_.csdh-4B**** | **2010** | **wPt-3917** | **127.4** | **3.6** | **10.6** | **-0.590** |
|  | **2** | **D** | ***Qchl_a+b_.csdh-2D.1**** | **2010** | **wPt-6574** | **159.2** | **7.0** | **22.4** | **0.931** |
|  | **2** | **D** | ***Qchl_a+b_.csdh-2D.2**** | **2010** | **wPt-730613** | **174.0** | **4.3** | **16.7** | **0.878** |
|  | 2 | D | *Qchl_a+b_.csdh-2D.3** | 2010 | gwm349 | 200.4 | 4.3 | 13.0 | -0.720 |
|  | **7** | **D** | ***Qchl_a+b_.csdh-7D*** | **2008** | **wPt-743332** | **14.6** | **2.6** | **8.3** | **0.239** |
| **SPAD** | **1** | **B** | ***QSPAD.csdh-1B.1**** | **2010** | **wPt-2389** | **86.1** | **4.4** | **15.0** | **1.835** |
|  | **1** | **B** | ***QSPAD.csdh-1B.2**** | **2011** | **wPt-3451** | **90.3** | **4.4** | **11.6** | **1.374** |
|  | **4** | **B** | ***QSPAD.csdh-4B.1**** | **2011** | ***Rht-B1*** | **65.2** | **5.7** | **15.6** | **-1.571** |
|  | **4** | **B** | ***QSPAD.csdh-4B.2**** | **2010** | **gwm6a** | **105.6** | **3.5** | **11.0** | **-1.543** |
|  | **5** | **B** | ***QSPAD.csdh-5B*** | **2010** | **psr120.2** | **106.2** | **2.6** | **9.0** | **-1.419** |
|  | **6** | **B** | ***QSPAD.csdh-6B.1**** | **2011** | **wPt-2424** | **71.7** | **4.4** | **12.2** | **-1.478** |
|  | **6** | **B** | ***QSPAD.csdh-6B.2**** | **2011** | **gwm191** | **79.2** | **4.8** | **13.6** | **-1.546** |
|  | **6** | **B** | ***QSPAD.csdh-6B.3**** | **2011** | **wPt-2564** | **89.5** | **4.5** | **13.5** | **-1.566** |
|  | **1** | **D** | ***QSPAD.csdh-1D*** | **2011** | **wPt-729826** | **143.0** | **2.1** | **§** | **-ve** |
|  | **2** | **D** | ***QSPAD.csdh-2D**** | **2010** | **wPt-6574** | **159.2** | **5.4** | **17.4** | **1.952** |
|  | **2** | **D** | ***QSPAD.csdh-2D**** | **2011** | **wPt-6574** | **159.2** | **3.4** | **8.8** | **1.200** |
|  | **5** | **D** | ***QSPAD.csdh-5D*** | **2010** | **wmc233** | **15.8** | **2.6** | **7.7** | **-1.290** |
|  | **7** | **D** | ***QSPAD.csdh-7D*** | **2011** | **wPt-744354** | **185.0** | **2.9** | **7.6** | **-1.144** |
| **Car** | **3** | **A** | ***QCar.csdh-3A.1**** | **2008** | **wPt-2478** | **21.1** | **3.2** | **11.0** | **-0.031** |
|  | **3** | **A** | ***QCar.csdh-3A.2**** | **2008** | **wPt-4569** | **102.5** | **4.1** | **14.2** | **0.036** |
|  | 4 | A | *QCar.csdh-4A* | 2007 | psr160.1 | 18.0 | 3.3 | 10.8 | 0.135 |
|  | **1** | **B** | ***QCar.csdh-1B*** | **2007** | **wPt-8280** | **111.7** | **2.8** | **8.6** | **0.116** |
|  | **4** | **B** | ***QCar.csdh-4B.1*** | **2007** | **wPt-733745** | **76.4** | **3.2** | **10.1** | **-0.126** |
|  | **4** | **B** | ***QCar.csdh-4B.2*** | **2010** | **psr375.4** | **128.2** | **3.3** | **10.4** | **-0.122** |
|  | **2** | **D** | ***QCar.csdh-2D*** | **2010** | **wPt-6574** | **159.2** | **3.3** | **10.4** | **0.124** |
|  | 3 | D | *QCar.csdh-3D.1* | 2010 | dupw173 | 12.4 | 3.0 | 10.0 | -0.121 |
|  | **3** | **D** | ***QCar.csdh-3D.2*** | **2010** | **wPt-4569** | **45.1** | **3.4** | **32.0** | **-0.212** |
|  | **4** | **D** | ***QCar.csdh-4D**** | **2007** | **psr375.1** | **134.7** | **3.8** | **12.3** | **-0.142** |
|  | **5** | **D** | ***QCar.csdh-5D**** | **2008** | **GS2-like_463** | **40.2** | **3.2** | **10.6** | **-0.031** |
|  | **6** | **D** | ***QCar.csdh-6D.1**** | **2007** | **wPt-665675** | **154.6** | **3.4** | **13.4** | **0.146** |
|  | **6** | **D** | ***QCar.csdh-6D.2*** | **2010** | **wPt-734218** | **161.6** | **3.2** | **10.0** | **0.121** |
|  | **6** | **D** | ***QCar.csdh-6D.3**** | **2007** | **wPt-732626** | **166.8** | **4.2** | **13.8** | **0.151** |
|  | **7** | **D** | ***QCar.csdh-7D.1*** | **2010** | **barc154** | **71.2** | **2.0** | **6.2** | **0.095** |
|  | **7** | **D** | ***QCar.csdh-7D.2*** | **2010** | **mgl59** | **83.4** | **2.0** | **7.2** | **0.105** |
|  | 7 | D | *QCar.csdh-7D.3* | 2010 | wmc488a | 128.4 | 2.1 | 5.6 | 0.090 |
| **DWP** | **5** | **A** | ***QDWP.csdh-5A**** | **2010** | **wPt-668257** | **111.7** | **3.6** | **11.3** | **0.380** |
|  | 6 | A | *QDWP.csdh-6A* | 2008 | wPt-1664 | 14.9 | 2.4 | § | -ve |
|  | **7** | **A** | ***QDWP.csdh-7A.1*** | **2008** | **m67p77.4** | **125.6** | **2.9** | **10.9** | **-0.357** |
|  | **7** | **A** | ***QDWP.csdh-7A.2*** | **2010** | **wPt-4810** | **151.9** | **2.2** | **6.5** | **-0.286** |
|  | **7** | **A** | ***QDWP.csdh-7A.3*** | **2010** | **m51p65.7** | **169.2** | **2.1** | **7.9** | **-0.313** |
|  | **1** | **B** | ***QDWP.csdh-1B*** | **2008** | **wPt-8682** | **32.5** | **2.6** | **9.1** | **0.338** |
|  | **2** | **B** | ***QDWP.csdh-2B*** | **2007** | **gwm148** | **86.3** | **2.5** | **8.5** | **-0.466** |
|  | **4** | **B** | ***QDWP.csdh-4B.1*** | **2010** | ***Rht-B1*** | **65.2** | **2.3** | **§** | **+ve** |
|  | **4** | **B** | ***QDWP.csdh-4B.1*** | **2008** | ***Rht-B1*** | **65.2** | **2.8** | **9.7** | **0.335** |
|  | **4** | **B** | ***QDWP.csdh-4B.2**** | **2007** | **psp3030b** | **74.0** | **3.9** | **14.7** | **0.620** |
|  | **4** | **B** | ***QDWP.csdh-4B.2*** | **2010** | **psp3030b** | **74.0** | **2.1** | **§** | **+ve** |
|  | **7** | **B** | ***QDWP.csdh-7B*** | **2007** | **wPt-3402** | **251.1** | **2.1** | **7.5** | **0.443** |
|  | **3** | **D** | ***QDWP.csdh-3D**** | **2010** | **dupw173** | **11.4** | **3.5** | **11.6** | **-0.390** |
| **GWE** | **3** | **A** | ***QGwe.csdh-3A*** | **2007** | **psr598** | **57.6** | **2.5** | **8.8** | **0.091** |
|  | **5** | **A** | ***QGwe.csdh-5A*** | **2007** | **psr150** | **65.5** | **2.2** | **6.9** | **0.079** |
|  | **7** | **A** | ***QGwe.csdh-7A.1**** | **2008** | **wmc422** | **131.4** | **3.6** | **12.1** | **-0.092** |
|  | **7** | **A** | ***QGwe.csdh-7A.2*** | **2010** | **m51p65.7** | **169.2** | **2.2** | **9.4** | **-0.074** |
|  | **7** | **A** | ***QGwe.csdh-7A.3**** | **2007** | **m68p78.6** | **183.4** | **3.4** | **11.0** | **-0.100** |
|  | **2** | **B** | ***QGwe.csdh-2B.1*** | **2008** | **m65p64.5** | **161.8** | **3.7** | **§** | **-ve** |
|  | **2** | **B** | ***QGwe.csdh-2B.2**** | **2010** | **wPt-2397** | **169.2** | **3.9** | **14.1** | **0.120** |
|  | **6** | **B** | ***QGwe.csdh-6B.1**** | **2007** | **wg232.4** | **81.9** | **3.6** | **11.6** | **-0.104** |
|  | **6** | **B** | ***QGwe.csdh-6B.2*** | **2007** | ***GS1*** | **92.0** | **2.7** | **§** | **-ve** |
|  | **6** | **B** | ***QGwe.csdh-6B.3*** | **2010** | **wPt-5211** | **94.0** | **2.2** | **§** | **-ve** |
|  | **4** | **D** | ***QGwe.csdh-4D**** | **2008** | **gwm165b** | **55.9** | **4.3** | **14.3** | **0.100** |
| **YP** | 6 | A | *QYld.csdh-6A* | 2008 | wPt-9075 | 7.4 | 2.6 | § | **-ve** |
|  | **7** | **A** | ***QYld.csdh-7A.1*** | **2007** | **psp3050** | **129.4** | **2.4** | **8.9** | **-0.270** |
|  | **7** | **A** | ***QYld.csdh-7A.2**** | **2010** | **wPt-4810** | **151.9** | **3.8** | **13.0** | **-0.227** |
|  | **7** | **A** | ***QYld.csdh-7A.3**** | **2010** | **m51p65.7** | **161.2** | **4.6** | **15.6** | **-0.246** |
|  | **1** | **B** | ***QYld.csdh-1B.1**** | **2008** | **wPt-8682** | **32.5** | **3.5** | **12.7** | **0.232** |
|  | 1 | B | *QYld.csdh-1B.2* | 2007 | wPt-9787 | 48.3 | 2.1 | 7.5 | -0.511 |
|  | **1** | **B** | ***QYld.csdh-1B.3**** | **2007** | **csu109** | **65.3** | **4.0** | **16.4** | **0.751** |
|  | **2** | **B** | ***QYld.csdh-2B*** | **2010** | **wPt-2397** | **169.2** | **3.3** | **10.7** | **0.200** |
|  | **7** | **B** | ***QYld.csdh-7B*** | **2010** | **wPt-9665** | **146.8** | **2.6** | **8.3** | **0.181** |
|  | **4** | **D** | ***QYld.csdh-4D*** | **2008** | **psp3103** | **6.0** | **2.5** | **12.8** | **-0.251** |
