# Supplementary material for: Quantitative trait loci for leaf chlorophyll fluorescence parameters, chlorophyll and carotenoid contents in relation to biomass and yield in bread wheat and their chromosome deletion bin assignments
Source: Mol Breed. 2013 Apr 10;32(1):189–210. doi: 10.1007/s11032-013-9862-8 (PMC3684715; doi:10.1007/s11032-013-9862-8)
Supplement: Supplementary file 5 — Supplementary material 5 (DOC 309 kb) [file 11032_2013_9862_MOESM5_ESM.doc]

**Quantitative trait loci for leaf chlorophyll fluorescence parameters, chlorophyll and carotenoid contents in relation to biomass and yield in bread wheat and their chromosome deletion bin assignments**

Czyczyło-Mysza I.^1^, Tyrka M.^2^, Marcińska I.^1^, Skrzypek E.^1^, Karbarz M.^3^, Dziurka M.^1^, Hura T.^1^, Dziurka K.^1^, Quarrie S.A.^4^

^1^ The *F. Górski* Institute of Plant Physiology, Polish Academy of Sciences, Kraków, Poland

^2^ Rzeszów University of Technology, Department of Biochemistry and Biotechnology, Poland.

^3^ Institute of Applied Biotechnology and Basic Sciences, University of Rzeszow

^4^ Faculty of Biology, Belgrade University, Serbia, and Visiting Professor, Newcastle University, UK.

Corresponding author: [czyczylo-mysza@wp.pl](javascript:oknoAdresat('napisz.html?to=czyczylo-mysza@wp.pl',10,10,650,540,1);)

**Table S5** Coincidences of trait QTLs with other QTLs and genes. For each chromosome coincident QTL region, trait QTLs are ranked in order of decreasing LODmax, and cM distance (from the top of the linkage group) is given to the QTL with the greatest LODmax. The nearest flanking marker information is given for the QTL with the highest LODmax score, and indicates the markers closest to the QTL threshold LOD of 2.0. For coincidences with multiple genes, the gene with the best coincidence is placed at the top of the group. Candidate genes for one or more of the QTLs are underlined. * indicates QTLs significant at 1000 permutations (LODmax typically above 3.3). Other QTLs with LODmax from 1.8 to typically 3.0 did not reach significance, but are included for comparison with other QTLs and coincident genes.

| **Chromo- some QTL region** | **Traits** | Year | QTL LODmax | Marker nearest LODmax | cM to greatest LODmax | Flanking markers | **LODmax bin location** | **Coincident gene(s)** |
| --- | --- | --- | --- | --- | --- | --- | --- | --- |
| 1A-I | ABS/CSm | 2011 | 2.8 | m51.p65.5 | 20.2 | tPt-5413 - wPt-5776 | 0.86-1.00S |  |
|  | ET0/CSm | 2011 | 1.9 |  |  |  |  |  |
| 1A-II | TR0/CSm* | 2008 | 3.6 | wPt-731617 | 42.6 | wPt-3904 - wPt-7074 | 0.78-0.86S |  |
|  | ABS/CSm* | 2008 | 3.4 |  |  |  |  |  |
|  | TR0/CSm | 2007 | 3.1 |  |  |  |  |  |
|  | ABS/CSm | 2007 | 3.1 |  |  |  |  |  |
| 1A-III | ABS/CSm | 2007 | 1.9 | psp3003a | 56.3 | wPt-7074 - psp3003 | C-0.17L |  |
|  | TR0/CSm | 2007 | 1.8 |  |  |  |  |  |
| 1B-I | YP | 2008 | 3.5 | wPt-8682 | 32.5 | wPt-0974 - wPt-6078 | 0.84-1.06S |  |
|  | DWP | 2008 | 2.6 |  |  |  |  |  |
| 1B-II | SPAD* | 2011 | 4.4 | wPt-2389 | 90.3 | gwm11 - wPt-3566 | 0.32-0.47L | Chlorophyll a/b binding protein upstream region (*Cab-1*) |
|  | SPAD* | 2010 | 4.4 |  |  |  |  | Sucrose export defective 1 (*Sxd1*) |
|  | TR0/CSm | 2010 | 2.4 |  |  |  |  |  |
| 1D-I | DI0/CSm* | 2010 | 5.3 | wPt-4671 | 77.4 | rPt-4471 - wPt-6316 | C |  |
|  | TR0/CSm | 2010 | 2.1 |  |  |  |  |  |
| 1D-II | SPAD | 2011 | 2.1 | wPt-729826 | 143.0 | wPt-732556 - wPt-729826 | 0.41-1.00L | Chlorophyll a/b binding protein 2 (*Cab2*) |
|  |  |  |  |  |  |  |  | Cytochrome P450 (*CytP*) |
|  |  |  |  |  |  |  |  | Glutamyl-tRNA reductase (*GluTR*) |
| 1D-III | Fv/Fm | 2011 | 3.3 | m59p78.0 | 188.9 | wPt-1685 - m59p78.0 | 0.41-1.00L | Chlorophyll a/b binding protein 2 (*Cab2*) |
|  |  |  |  |  |  |  |  | Cytochrome P450 (*CytP*) |
|  |  |  |  |  |  |  |  | Glutamyl-tRNA reductase (*GluTR*) |
| 2A-I | ABS/CSm | 2008 | 1.9 | m85p65.8 | 77.2 | wPt-743211 - m83p65.2 | C-0.85S | Ribulose-1,5-bisphosphate carboxylase/oxygenase small subunit (Rubisco-ssu) |
|  |  |  |  |  |  |  |  | Ferredoxin-NADP(H) oxidoreductase (*Fe-NADP(H)*) |
| 2A-II | ET0/CSm | 2008 | 1.8 | m83p65.2 | 83.2 | m83p65.2 | C-0.85S | Ribulose-1,5-bisphosphate carboxylase/oxygenase small subunit (*Rubisco-ssu*) |
|  |  |  |  |  |  |  |  | Ferredoxin-NADP(H) oxidoreductase (*Fe-NADP(H)*) |
| 2A-III | Fv/Fm* | 2011 | 3.6 | gwm339 | 106.1 | wPt-9320 - wPt-3114 | C | Zeta-carotene desaturase (*Zds*) |
|  | ET0/CSm* | 2007 | 3.4 |  |  |  |  | Sucrose synthase type 1 (*Sus2*) |
|  | PI* | 2007 | 3.4 |  |  |  |  | Photosystem I reaction center subunit psaK (*PSI-K*) |
|  | TR0/CSm | 2011 | 2.2 |  |  |  |  | Chlorophyll a/b binding protein CP24 10A, (LHCP) (*Cab-10A*) |
|  | ET0/CSm | 2011 | 1.8 |  |  |  |  | L-ascorbate peroxidase (thylakoid lumenal 29 kDa protein) |
|  |  |  |  |  |  |  |  | Beta-carotene hydroxylase 3 (*Hyd3*) |
|  |  |  |  |  |  |  |  | Polyphenol oxidase 1 (*Ppo1*) |
|  |  |  |  |  |  |  |  | Coproporphyrinogen III oxidase (*CopIII-ox*) |
| 2A-IV | ABS/CSm | 2010 | 2.4 | wPt-729945 | 180.8 | cfd50b - wPt-9793 | 0.85-1.00L |  |
|  | TR0/CSm | 2010 | 2.3 |  |  |  |  |  |
| 2B-I | RC/CSm | 2011 | 3.0 | wPt-4613 | 4.5 | barc124c - wmc243a | 0.84-1.00S | Ribulose-1,5-bisphosphate carboxylase/oxygenase subunit binding-protein alpha subunit (CPN-60 alpha) |
|  | PI | 2011 | 2.2 |  |  |  |  | Acetyl-coenzyme A carboxylase (*ACCase*) |
| 2B-II | Chla+b | 2010 | 1.9 | wmc477 | 108.4 | wPt-0335 - gwm55 | C-0.53S | Sucrose synthase type 1 (*Sus2*) |
| 2B-III | RC/CSm | 2008 | 3.0 | psr331.2 | 133.5 | barc101a - wPt-3755 | 0.89L | Polyphenol oxidase 1 (*Ppo1*) |
|  | PI | 2008 | 2.8 |  |  |  |  | Chlorophyll a/b binding protein CP24 10A, (LHCP) (*Cab-10A*) |
|  |  |  |  |  |  |  |  | Coproporphyrinogen III oxidase (*CopIII-ox*) |
| 2B-IV | ABS/CSm* | 2008 | 3.4 | wPt-8776 | 155.5 | wPt-667945 - m77p64.11 | 0.89-1.00L |  |
|  | TR0/CSm | 2008 | 3.0 |  |  |  |  |  |
|  | ET0/CSm* | 2008 | 3.0 |  |  |  |  |  |
|  | Fv/Fm | 2008 | 2.4 |  |  |  |  |  |
| 2B-V | GWE* | 2010 | 3.9 | wPt-8668 | 169.2 | wPt-4210 - wPt-744181 | 0.89-1.00L |  |
|  | ABS/CSm | 2008 | 3.8 |  |  |  |  |  |
|  | GWE | 2008 | 3.7 |  |  |  |  |  |
|  | TR0/CSm* | 2008 | 3.6 |  |  |  |  |  |
|  | Yp | 2011 | 3.3 |  |  |  |  |  |
|  | ET0/CSm | 2008 | 2.9 |  |  |  |  |  |
|  | Fv/Fm | 2008 | 2.4 |  |  |  |  |  |
| 2D-I | PI | 2010 | 2.5 | gwm539 | 129.5 | gwm539 - wPt-3728 | C-0.49L | Photosystem I reaction center subunit psaK (*PSI-K*) |
|  | ET0/CSm | 2010 | 1.9 |  |  |  |  | Photosystem II 10 kDa polypeptide (*PSII-10kDa*) |
|  |  |  |  |  |  |  |  | L-ascorbate peroxidase (thylakoid lumenal 29 kDa protein) |
|  |  |  |  |  |  |  |  | Polyphenol oxidase 1 (*Ppo1*) |
| 2D-II | Chla+b* | 2010 | 7.0 | wPt-6574 | 159.2 | wPt-7466 - cfd73 | C-0.49L | Beta-carotene hydroxylase 3 (*Hyd3*) |
|  | SPAD* | 2010 | 5.4 |  |  |  |  |  |
|  | SPAD* | 2011 | 3.9 |  |  |  |  |  |
|  | Car | 2010 | 3.3 |  |  |  |  |  |
|  | PI | 2007 | 3.0 |  |  |  |  |  |
| 2D-III | Chla+b* | 2010 | 4.3 | wPt-730613 | 174.0 | cfd73 - wPt-730613 | 0.76-1.00L | Coproporphyrinogen III oxidase (*CopIII-ox*) |
|  | Fv/Fm | 2007 | 3.2 |  |  |  |  |  |
|  | PI | 2007 | 2.6 |  |  |  |  |  |
| 2D-IV | Fv/Fm* | 2007 | 5.3 | gwm349 | 200.4 | wPt-7825 - wPt-1301 | 0.76-1.00L | Coproporphyrinogen III oxidase (*CopIII-ox*) |
|  | Chla+b* | 2010 | 4.3 |  |  |  |  |  |
|  | RC/CSm* | 2007 | 3.4 |  |  |  |  |  |
|  | TR0/CSm | 2007 | 3.2 |  |  |  |  |  |
|  | ABS/CSm | 2007 | 3.1 |  |  |  |  |  |
|  | ABS/CSm | 2008 | 2.4 |  |  |  |  |  |
|  | TR0/CSm | 2008 | 2.4 |  |  |  |  |  |
| 3A-I | Fv/Fm | 2011 | 2.9 | wPt-9928 | 4.5 | m60p64.5 - wmc532 | 0.45-1.00S | 1-deoxy-D-xylulose 5-phosphate reductoisomerase (*Dxr*) |
|  | Chla+b | 2008 | 2.4 |  |  |  |  | Sucrose-phosphate synthase (*Sps*) |
| 3A-II | Car* | 2008 | 3.2 | wPt-2478 | 21.1 | tPt-6949 - *Stb6* | 0.45-1.00S | Sucrose-phosphate synthase (*Sps*) |
| 3A-III | RC/CSm* | 2008 | 5.4 | cfa2234 | 64.5 | psr598 - cfa2262 | C-0.42L | Lycopene epsilon cyclase (*e-Lcy*) |
|  | PI* | 2008 | 4.6 |  |  |  |  |  |
|  | ET0/CSm | 2008 | 3.3 |  |  |  |  |  |
|  | GWE | 2007 | 2.5 |  |  |  |  |  |
| 3A-IV | Car* | 2008 | 4.1 | wPt-734079 | 102.5 | cfa2262 - wPt-3133 | 0.42-0.78L |  |
|  | ET0/CSm | 2008 | 3.3 |  |  |  |  |  |
|  | Chla+b | 2007 | 2.3 |  |  |  |  |  |
|  | PI | 2008 | 2.1 |  |  |  |  |  |
| 3D-I | DWP* | 2010 | 3.5 | dupw173 | 11.4 | gwm161 - wPt-4569 | 0.55-1.00S | 1-deoxy-D-xylulose 5-phosphate reductoisomerase (*Dxr*) |
|  | RC/CSm | 2010 | 3.1 |  |  |  |  |  |
|  | Car | 2010 | 3.0 |  |  |  |  |  |
|  | SPAD | 2010 | 2.1 |  |  |  |  |  |
| 3D-II | Car | 2010 | 3.4 | wPt-4569 | 45.1 | wPt-4569 - wPt-732092 | 0.55-1.00S |  |
|  | Chla+b | 2007 | 1.9 |  |  |  |  |  |
| 3D-III | DI0/CSm* | 2010 | 3.3 | wPt-732092 | 72.2 | wPt-732092 - wPt-741038 | 0.55-1.00S |  |
|  | ABS/CSm | 2010 | 3.1 |  |  |  |  |  |
|  | GWE | 2010 | 2.1 |  |  |  |  |  |
| 4A-I | Car | 2007 | 3.3 | psr160.1 | 18.0 | psr392.1 - wPt-4828 | 0.80-1.00L | Plastocyanin chloroplast precursor (*Plc*) |
| 4A-II | ABS/CSm | 2007 | 1.9 | wPt-3349 | 34.9 | wPt-3349 | 0.80-1.00L |  |
|  | TR0/CSm | 2007 | 1.8 |  |  |  |  |  |
| 4A-III | TR0/CSm | 2008 | 1.9 | mwg634.2 | 103.9 | dupw004a - mwg634.2 | 0.59-0.66L | Rubisco subunit binding-protein alpha subunit (*Rubisco-spb-a*) |
|  | ABS/CSm | 2008 | 1.9 |  |  |  |  |  |
| 4A-IV | PI* | 2008 | 3.9 | gwm30b | 125.7 | psr593.2 - mwg58 | C-0.20S | Chlorophyll a/b binding protein CP29 precursor (*Cab(CP29)*) |
|  | ET0/CSm* | 2008 | 3.5 |  |  |  |  | Ribulose 1,5-bisphosphate carboxylase activase (RcaA and RcaB) (*RcaA/B*) |
|  | RC/CSm | 2008 | 3.0 |  |  |  |  | Sucrose synthase (*Sus*) |
|  | TR0/CSm | 2008 | 2.2 |  |  |  |  | Oxygen-evolving complex 25.6 kD protein (*O-ec 25.6kDa*) |
|  | ABS/CSm | 2008 | 1.8 |  |  |  |  | Sucrose transporter 1 (*Sut1*) |
|  |  |  |  |  |  |  |  | Porphobilinogen deaminase (*Pbd*) |
|  |  |  |  |  |  |  |  | Phytoene desaturase (dehydrogenase) (*Pds*) |
| 4B-I | RC/CSm* | 2011 | 6.6 | Rht-B1 | 65.2 | psp3163 - psp3030b | C-0.37S | 4-hydroxy-3-methylbut-2-enyl diphosphate reductase [ispH] (*Hdr*) |
|  | SPAD* | 2011 | 5.7 |  |  |  |  |  |
|  | PI* | 2011 | 4.9 |  |  |  |  |  |
|  | DWP | 2008 | 2.8 |  |  |  |  |  |
|  | DWP | 2010 | 2.3 |  |  |  |  |  |
|  | ET0/CSm | 2011 | 1.9 |  |  |  |  |  |
| 4B-II | DWP* | 2007 | 3.9 | psp3030b | 74.0 | *Rht-B1* - gwm165a | C-0.71L | Sucrose synthase (*Sus*) |
|  | Car | 2007 | 3.2 |  |  |  |  | Oxygen-evolving complex 25.6 kD protein (*O-ec 25.6kDa*) |
|  | DWP | 2010 | 2.1 |  |  |  |  | Sucrose transporter 1 (*Sut1*) |
| 4B-III | PI | 2007 | 2.5 | barc60 | 98.4 | wPt-733038 - gwm6a | C-0.71L | Oxygen-evolving complex 25.6 kD protein (*O-ec 25.6kDa*) |
|  | ET0/CSm | 2007 | 2.0 |  |  |  |  | Sucrose transporter 1 (*Sut1*) |
| 4B-IV | SPAD* | 2010 | 3.5 | gwm6a | 105.6 | barc60 - wPt-0391 | 0.86-1.00L | Phytoene desaturase (dehydrogenase) (*Pds*) |
|  | PI | 2010 | 2.5 |  |  |  |  | Uroporphyrinogen III synthase (*UpoIIIs*) |
|  | PI | 2007 | 1.8 |  |  |  |  |  |
| 4B-V | Chla+b* | 2010 | 3.6 | psr375.4 | 127.4 | wmc47 - dupw043 | 0.86-1.00L | Phytoene desaturase (dehydrogenase) (*Pds*) |
|  | Car | 2010 | 3.3 |  |  |  |  | Uroporphyrinogen III synthase (*UpoIIIs*) |
| 4D-I | YP | 2008 | 2.5 | psp3103 | 6.0 | psp3103 | C-0.53S | Ribulose 1,5-bisphosphate carboxylase activase (RcaA and RcaB) (*RcaA/B*) |
|  |  |  |  |  |  |  |  | 4-hydroxy-3-methylbut-2-enyl diphosphate reductase [ispH] (*Hdr*) |
| 4D-II | ET0/CSm* | 2007 | 6.0 | wPt-5809 | 35.0 | psp3103 - blt101.t7 | C-0.53S | Ribulose 1,5-bisphosphate carboxylase activase (RcaA and RcaB) (*RcaA/B*) |
|  | PI* | 2007 | 5.6 |  |  |  |  | 4-hydroxy-3-methylbut-2-enyl diphosphate reductase [ispH] (*Hdr*) |
|  | SPAD | 2010 | 2.1 |  |  |  |  | Porphobilinogen deaminase (*Pbd*) |
|  | Fv/Fm | 2007 | 1.8 |  |  |  |  |  |
| 4D-III | GWE* | 2008 | 4.3 | gwm165b | 55.9 | wPt-5089 - psr375.1 | 0.31S | Oxygen-evolving complex 25.6 kD protein (*O-ec 25.6kDa*) |
|  |  |  |  |  |  |  |  | Photosystem II 10K protein (*PSII-10kDa*) |
|  |  |  |  |  |  |  |  | Sucrose transporter 1 (*Sut1*) |
| 4D-IV | Car* | 2007 | 3.8 | psr375.1 | 134.7 | psr375.1 - gwm609 | 0.71-1.00L | Uroporphyrinogen III synthase (*UpoIIIs*) |
| 5A-I | GWE | 2007 | 2.2 | psr150 | 65.5 | barc14 - wPt-9748 | 0.35-0.57L | Ribulose-1,5-bisphosphate carboxylase/oxygenase small subunit (*Rubisco-ssu*) |
|  |  |  |  |  |  |  |  | Porphobilinogen deaminase (*Pdb*) |
|  |  |  |  |  |  |  |  | Chlorophyll a/b-binding protein precursor (*Lhca4*) |
|  |  |  |  |  |  |  |  | Ferrochelatase II (Protoheme ferro-lyase) (Heme synthetase) (*Phe-Fel*) |
| 5A-II | PI* | 2010 | 3.8 | psp3003b | 112.8 | psr637 - wPt-4262 | 0.68-0.78L | Chlorophyll a/b binding protein (*Cab1.2*) |
|  | DWP* | 2010 | 3.6 |  |  |  |  | Phytoene synthase 3 (*Psy3-A*) |
| 5A-III | Chla+b* | 2010 | 3.2 | wmc388b | 119.5 | wPt-668257 - psr575.2 | 0.68-0.78L | Photosystem-1 F subunit precursor (*PSF-I*) |
|  | Chla+b | 2008 | 3.0 |  |  |  |  | Staygreen (rice sgr homolog) (*Sgr*) |
| 5A-IV | RC/CSm* | 2007 | 3.3 | Vrn-A1 | 130.6 | psr2021.1 - psr426.1 | 0.68-0.78L | Photosystem-1 F subunit precursor (*PSF-I*) |
|  | DI0/RCm | 2007 | 3.2 |  |  |  |  | Staygreen (rice sgr homolog) (*Sgr*) |
|  | Chla+b | 2008 | 2.1 |  |  |  |  | Chlorophyll a/b binding protein (*Cab1.1*) |
| 5A-V | ET0/CSm | 2010 | 1.8 | wPt-1903 | 186.4 | wPt-1903 - wPt-5231 | 0.87-1.00L | Sucrose-phosphate synthase (*Sps*) |
| 5B-I | RC/CSm* | 2008 | 5.6 | wPt-5346 | 30.6 | wg232.2 - psr326.2 | 0.71-0.81S |  |
|  | DI0/CSm* | 2008 | 4.1 |  |  |  |  |  |
|  | PI* | 2008 | 3.8 |  |  |  |  |  |
|  | ET0/CSm* | 2008 | 3.6 |  |  |  |  |  |
|  | DI0/CSm* | 2007 | 3.4 |  |  |  |  |  |
|  | ABS/CSm | 2008 | 2.9 |  |  |  |  |  |
| 5B-II | DI0/CSm* | 2008 | 4.3 | wmc73 | 42.8 | psr326.2 - wPt-1951 | 0.29-0.71S | Chlorophyll a/b-binding protein precursor (*Lhca4*) |
|  | DI0/CSm | 2007 | 2.9 |  |  |  |  | Ferrochelatase II (Protoheme ferro-lyase) (Heme synthetase) (*Phe-Fel*) |
| 5B-III | DI0/CSm | 2008 | 2.7 | psr725 | 69.8 | wPt-7418 - gwm639b | 0.55-0.75L | Ribulose-1,5-bisphosphate carboxylase/oxygenase small subunit (*Rubisco-ssu*) |
|  | ABS/CSm | 2011 | 2.0 |  |  |  |  |  |
| 5B-IV | ABS/CSm* | 2011 | 3.7 | psp3037 | 78.7 | gwm499 - psp3073 | 0.55-0.75L |  |
|  | TR0/CSm | 2011 | 3.2 |  |  |  |  |  |
| 5B-V | Fv/Fm* | 2010 | 4.6 | wPt-1548 | 99.0 | psr806.2 - m65p64.8a | 0.75-0.76L | Phytoene synthase 3 (*Psy3-B*) |
|  | TR0/CSm* | 2010 | 4.3 |  |  |  |  |  |
|  | ABS/CSm | 2010 | 3.3 |  |  |  |  |  |
|  | ET0/CSm | 2010 | 2.7 |  |  |  |  |  |
|  | SPAD | 2010 | 2.6 |  |  |  |  |  |
| 5B-VI | ABS/CSm | 2010 | 2.1 | m65p64.8a | 113.5 | m65p64.8a - psr2021.2 | 0.76L |  |
|  | TR0/CSm | 2010 | 1.8 |  |  |  |  |  |
| 5D-I | SPAD | 2010 | 2.6 | wmc233 | 15.8 | wmc233 - gwm190 | 0.78S | Carotenoid cleavage dioxygenase 1 (*Ccd1*) |
|  |  |  |  |  |  |  |  | Sucrose transporter 2 (*Sut2*) |
| 5D-II | Car* | 2008 | 3.2 | GS2-like _463 | 40.2 | wPt-2495 - barc44 | C-0.63S | Ribulose-1,5-bisphosphate carboxylase/oxygenase small subunit (*Rubisco-ssu*) |
| 5D-III | PI | 2007 | 2.1 | m77p64.8 | 157.0 | cfd7 - gwm292 | 0.74-0.76L | Phytoene synthase 3 (*Psy3-D*) |
|  | SPAD | 2010 | 1.9 |  |  |  |  |  |
| 5D-IV | PI | 2010 | 2.1 | gwm212 | 171.1 | gwm292 - gwm212 | 0.76-1.00L | Rubisco subunit binding-protein alpha subunit (*Rubisco-spb-a*) |
|  | RC/CSm | 2007 | 2.0 |  |  |  |  | Staygreen (rice sgr homolog) (*Sgr*) |
|  |  |  |  |  |  |  |  | Magnesium-chelatase subunit chlD (Mg-protoporphyrin IX chelatase) (*Mg-PpoIXc*) |
| 5D-V | DI0/CSm | 2008 | 2.1 | m62p64.9a | 198.9 | wmc97 - m62p64.9a | 0.76-1.00L | Rubisco subunit binding-protein alpha subunit (*Rubisco-spb-a*) |
|  |  |  |  |  |  |  |  | Staygreen (rice sgr homolog) (*Sgr*) |
|  |  |  |  |  |  |  |  | Magnesium-chelatase subunit chlD (Mg-protoporphyrin IX chelatase) (*Mg-PpoIXc*) |
|  |  |  |  |  |  |  |  | Photosystem II protein W-like protein (*PSII-W*) |
|  |  |  |  |  |  |  |  | Photosystem-1 F subunit precursor (*PSI-F*) |
| 5D-VI | PI | 2008 | 2.0 | m92p78.9 | 242.9 | m92p78.9 - gwm565 | 0.76-1.00L | Photosystem II protein W-like protein (*PSII-W*) |
|  | RC/CSm | 2007 | 1.8 |  |  |  |  | Photosystem-1 F subunit precursor (*PSI-F*) |
| 6A-I | YP | 2008 | 2.6 | wPt-9075 | 7.4 | m87p78.7 - gwm334 | 0.65-1.00S | Chlorophyll a/b-binding protein Wcab precursor (*Wcab*) |
|  | DWP | 2008 | 2.4 |  |  |  |  | Ferredoxin-NADP(H) oxidoreductase (*Fe-NADP(H)*) |
| 6A-II | ET0/CSm* | 2007 | 4.0 | wPt-667844 | 48.5 | wmc398b - wPt-7063 | C-0.65S | Rieske iron-sulfur protein (*RISP*) |
| 6A-III | Fv/Fm* | 2008 | 4.1 | csb112(*Dhn5*) | 97.2 | wPt-0902 - wPt-732183 | 0.90-1.00L | Protoporphyrinogen oxidase (Ppo I) (protoporphyrinogen IX oxidase isozyme I) (Ppx I) (*Ppo1(Ppx1)*) |
| 6B-I | ET0/CSm* | 2007 | 6.5 | wPt-2424 | 71.7 | gwm325 - wPt-2000 | C-0.76S |  |
|  | TR0/CSm* | 2007 | 4.4 |  |  |  |  |  |
|  | ABS/CSm* | 2007 | 4.4 |  |  |  |  |  |
|  | SPAD* | 2011 | 4.3 |  |  |  |  |  |
|  | PI* | 2007 | 3.9 |  |  |  |  |  |
|  | Fv/Fm | 2007 | 2.7 |  |  |  |  |  |
|  | GWE | 2007 | 2.0 |  |  |  |  |  |
| 6B-II | ET0/CSm* | 2007 | 7.4 | wg232.4 | 81.9 | wPt-2424 - *GS1* | C-0.76S | Lycopene beta cyclase (*b-Lcy*) |
|  | SPAD* | 2011 | 4.8 |  |  |  |  | Photosystem II 10 kDa polypeptide (*PSII-10kDa*) |
|  | TR0/CSm* | 2007 | 4.3 |  |  |  |  |  |
|  | ABS/CSm* | 2007 | 4.3 |  |  |  |  |  |
|  | PI* | 2007 | 3.9 |  |  |  |  |  |
|  | GWE* | 2007 | 3.6 |  |  |  |  |  |
|  | Fv/Fm | 2007 | 2.9 |  |  |  |  |  |
|  | RC/CSm | 2010 | 2.8 |  |  |  |  |  |
| 6B-III | ET0/CSm* | 2007 | 6.8 | wPt-2564 | 87.5 | wPt-3060 - *GS1* | C-0.36L | Lycopene beta cyclase (*b-Lcy*) |
|  | SPAD* | 2011 | 4.5 |  |  |  |  | Photosystem II 10 kDa polypeptide (*PSII-10kDa*) |
|  | TR0/CSm* | 2007 | 4.2 |  |  |  |  |  |
|  | ABS/CSm* | 2007 | 4.2 |  |  |  |  |  |
|  | RC/CSm | 2010 | 3.9 |  |  |  |  |  |
|  | GWE | 2007 | 2.7 |  |  |  |  |  |
|  | GWE | 2010 | 2.2 |  |  |  |  |  |
| 6B-IV | PI | 2008 | 5.5 | wPt-0406 | 163.6 | gwm608c - wPt-0406 | 0.40-1.00L | Photosystem II 10 kDa polypeptide (*PSII-10kDa*) |
|  | RC/CSm | 2008 | 5.0 |  |  |  |  | Porphobilinogen deaminase (*Pbd*) |
|  | ET0/CSm | 2008 | 2.9 |  |  |  |  |  |
| 6D-I | Car* | 2007 | 3.4 | wPt-665675 | 154.6 | m69p78.10 - wPt-3127 | 0.80-1.00L | Photosystem II 10 kDa polypeptide (*PSII-10kDa*) |
|  | ET0/CSm | 2007 | 2.7 |  |  |  |  |  |
|  | PI | 2007 | 2.6 |  |  |  |  |  |
|  | Car | 2010 | 2.5 |  |  |  |  |  |
|  | RC/CSm | 2010 | 2.1 |  |  |  |  |  |
|  | ABS/CSm | 2007 | 2.0 |  |  |  |  |  |
|  | TR0/CSm | 2007 | 2.0 |  |  |  |  |  |
| 6D-II | Car* | 2007 | 4.2 | wPt-741831 | 166.8 | wPt-3127 - wPt-741831 | 0.80-1.00L | Photosystem II 10 kDa polypeptide (*PSII-10kDa*) |
|  | Car | 2010 | 3.2 |  |  |  |  |  |
|  | ET0/CSm | 2007 | 2.6 |  |  |  |  |  |
|  | PI | 2007 | 2.1 |  |  |  |  |  |
|  | RC/CSm | 2010 | 2.1 |  |  |  |  |  |
|  | ABS/CSm | 2007 | 2.1 |  |  |  |  |  |
|  | TR0/CSm | 2007 | 2.0 |  |  |  |  |  |
| 7A-I | PI* | 2007 | 3.9 | wPt-9641 | 2.0 | wPt-9641 - wPt-666456 | 0.89-1.00S | Plastocyanin chloroplast precursor (*Plc*) |
|  | ET0/CSm | 2007 | 2.9 |  |  |  |  |  |
| 7A-II | ET0/CSm | 2007 | 2.2 | m39p78.4 | 16.9 | wPt-666456 - wmc388c | 0.89-1.00S | Plastocyanin chloroplast precursor (*Plc*) |
|  | PI | 2007 | 1.8 |  |  |  |  | Sucrose synthase 2 (*Sus*) |
| 7A-III | Fv/Fm | 2010 | 2.8 | wmc283a | 77.2 | wPt-9796 - wmc283a | 0.59-0.89S | Glutamyl-tRNA reductase (1st isoform) (*GluTR*) |
|  | Chla+b | 2008 | 2.0 |  |  |  |  | Sucrose synthase type 1 (*Sus*) |
| 7A-IV | Fv/Fm* | 2010 | 4.0 | barc108a | 128.3 | barc174 - m21p76.6 | C-0.39L |  |
|  | GWE* | 2008 | 3.6 |  |  |  |  |  |
|  | DWP | 2008 | 2.9 |  |  |  |  |  |
|  | YP | 2007 | 2.4 |  |  |  |  |  |
| 7A-V | YP | 2010 | 3.8 | wPt-4810 | 151.9 | wmc488b - wg232.6 | 0.39-.071L | Protoporphyrin IX magnesium chelatase subunit (*Xantha-h*) |
|  | DWP | 2010 |  |  |  |  |  | Delta-aminolevulinic acid dehydratase (porphobilinogen synthase) (ALADH) (*Aladh*) |
| 7A-VI | YP* | 2010 | 4.6 | m51p65.7 | 161.2 | wPt-4637 - m68p78.6 | 0.71-0.86L |  |
|  | GWE* | 2007 | 3.4 |  |  |  |  |  |
|  | GWE | 2010 | 2.2 |  |  |  |  |  |
|  | DWP | 2010 | 2.1 |  |  |  |  |  |
| 7A-VII | DI0/CSm | 2011 | 2.0 | psp3094a | 190.2 | psp3001a - wPt-6836 | 0.86-0.90L | Phytoene synthase (*Psy1-A1*) |
| 7B-I | YP | 2010 | 2.6 | wPt-9665 | 146.8 | m49p78.2 - m63p78.7 | 0.78-1.00L | Protoporphyrin IX magnesium chelatase subunit (*Xantha-h*) |
| 7B-II | GWE | 2008 | 1.8 | wmc276 | 221.5 | tPt-7247 | 0.78-1.00L | Phytoene synthase (*Psy1-B1*) |
| 7B-III | DPW | 2007 | 2.1 | wPt-3402 | 251.1 | wPt-4140 - wPt-744187 | 0.78-1.00L | Phytoene synthase (*Psy1-B1*) |
| 7D-I | Chla+b | 2008 | 2.6 | wPt-743332 | 14.6 | gwm635a - wPt-744300 | 0.61-1.00S | Plastocyanin chloroplast precursor (*Plc*) |
|  |  |  |  |  |  |  |  | Sucrose synthase 2 (*Sus*) |
| 7D-II | RC/CSm* | 2010 | 5.1 | barc154 | 71.2 | wmc463 - mgl59 | 0.61-1.00S |  |
|  | Car | 2010 | 2.0 |  |  |  |  |  |
| 7D-III | RC/CSm | 2010 | 3.6 | mgl59 | 83.0 | mgl59 - psp3035 | 0.36-0.61L |  |
|  | Car | 2010 | 2.0 |  |  |  |  |  |
|  | SPAD | 2011 | 1.8 |  |  |  |  |  |
| 7D-IV | PI* | 2007 | 3.4 | psp3094b | 168.1 | psp3045 - wPt-744354 | 0.61-0.82L | Protoporphyrin IX magnesium chelatase subunit (*Xantha-h*) |
| 7D-V | ET0/CSm* | 2007 | 4.8 | wPt-744354 | 179.4 | wmc157 - wPt-2054 | 0.82-1.00L | Phytoene synthase (*Psy1-D1*) |
|  | DI0/CSm* | 2010 | 4.6 |  |  |  |  |  |
|  | PI* | 2007 | 3.4 |  |  |  |  |  |
|  | SPAD | 2011 | 2.9 |  |  |  |  |  |
