# Supplementary material for: Quantitative trait loci for leaf chlorophyll fluorescence parameters, chlorophyll and carotenoid contents in relation to biomass and yield in bread wheat and their chromosome deletion bin assignments
Source: Mol Breed. 2013 Apr 10;32(1):189–210. doi: 10.1007/s11032-013-9862-8 (PMC3684715; doi:10.1007/s11032-013-9862-8)
Supplement: Supplementary file 6 — Supplementary material 6 (DOC 101 kb) [file 11032_2013_9862_MOESM6_ESM.doc]

**Table S6** Correlation matrix using phenotypic mean values for the 12 traits studied in three experiments.

| **E_I_ (2007)** | | **1** | **2** | **3** | **4** | **5** | **6** | **7** | **8** | **9** | **10** | **11** | **12** |
| --- | --- | --- | --- | --- | --- | --- | --- | --- | --- | --- | --- | --- | --- |
| **1** | **Fv/Fm_E_I_** | **1** |  |  |  |  |  |  |  |  |  |  |  |
| **2** | **PI_E_I_** | 0.824 | **1** |  |  |  |  |  |  |  |  |  |  |
| **3** | **ABS/CSm_E_I_** | 0.910 | 0.873 | **1** |  |  |  |  |  |  |  |  |  |
| **4** | **TRo/CSm_E_I_** | 0.935 | 0.875 | 0.998 | **1** |  |  |  |  |  |  |  |  |
| **5** | **ETo/CSm_E_I_** | 0.874 | 0.965 | 0.956 | 0.954 | **1** |  |  |  |  |  |  |  |
| **6** | **DIo/CSm_E_I_** | 0.024 | 0.327 | 0.432 | 0.372 | 0.413 | **1** |  |  |  |  |  |  |
| **7** | **RC/CSm_E_I_** | 0.804 | 0.951 | 0.901 | 0.896 | 0.935 | 0.442 | **1** |  |  |  |  |  |
| **8** | **Chla+b_E_I_** | 0.234 | 0.472 | 0.263 | 0.260 | 0.400 | 0.137 | 0.398 | **1** |  |  |  |  |
| **9** | **Car_E_I_** | 0.177 | 0.461 | 0.225 | 0.220 | 0.371 | 0.164 | 0.371 | 0.820 | **1** |  |  |  |
| **10** | **DWP_E_I_** | -0.033 | -0.040 | -0.044 | -0.043 | -0.051 | -0.035 | -0.066 | -0.086 | -0.071 | **1** |  |  |
| **11** | **GWE_E_I_** | 0.018 | -0.003 | -0.041 | -0.033 | -0.021 | -0.136 | -0.052 | 0.060 | 0.062 | 0.643 | **1** |  |
| **12** | **YP_E_I_** | 0.078 | 0.166 | 0.098 | 0.095 | 0.133 | 0.079 | 0.141 | 0.091 | 0.143 | 0.835 | 0.737 | **1** |
| **E_II_ (2008)** | | **1** | **2** | **3** | **4** | **5** | **6** | **7** | **8** | **9** | **10** | **11** | **12** |
| **1** | **Fv/Fm_E_II_** | **1** |  |  |  |  |  |  |  |  |  |  |  |
| **2** | **PI_E_II_** | 0.705 | **1** |  |  |  |  |  |  |  |  |  |  |
| **3** | **ABS/CSm_E_II_** | 0.926 | 0.794 | **1** |  |  |  |  |  |  |  |  |  |
| **4** | **TRo/CSm_E_II_** | 0.949 | 0.787 | 0.998 | **1** |  |  |  |  |  |  |  |  |
| **5** | **ETo/CSm_E_II_** | 0.810 | 0.956 | 0.907 | 0.900 | **1** |  |  |  |  |  |  |  |
| **6** | **DIo/CSm_E_II_** | -0.174 | 0.231 | 0.205 | 0.138 | 0.256 | **1** |  |  |  |  |  |  |
| **7** | **RC/CSm_E_II_** | 0.735 | 0.948 | 0.839 | 0.829 | 0.908 | 0.287 | **1** |  |  |  |  |  |
| **8** | **Chla+b_E_II_** | 0.085 | 0.396 | 0.148 | 0.138 | 0.320 | 0.170 | 0.358 | **1** |  |  |  |  |
| **9** | **Car_E_II_** | 0.003 | 0.259 | 0.039 | 0.033 | 0.163 | 0.101 | 0.252 | 0.848 | **1** |  |  |  |
| **10** | **DWP_E_II_** | 0.071 | 0.233 | 0.180 | 0.163 | 0.260 | 0.267 | 0.198 | 0.013 | -0.204 | **1** |  |  |
| **11** | **GWE_E_II_** | -0.122 | 0.091 | 0.000 | -0.022 | 0.085 | 0.307 | 0.065 | -0.065 | -0.126 | 0.749 | **1** |  |
| **12** | **YP_E_II_** | 0.070 | 0.241 | 0.161 | 0.147 | 0.264 | 0.228 | 0.180 | 0.134 | -0.060 | 0.927 | 0.661 | **1** |
| **E_III_ (2010)** | | **1** | **2** | **3** | **4** | **5** | **6** | **7** | **8** | **9** | **10** | **11** | **12** |
| **1** | **Fv/Fm_E_III_** | **1** |  |  |  |  |  |  |  |  |  |  |  |
| **2** | **PI_E_III_** | 0.707 | **1** |  |  |  |  |  |  |  |  |  |  |
| **3** | **ABS/CSm_E_III_** | 0.502 | 0.420 | **1** |  |  |  |  |  |  |  |  |  |
| **4** | **TRo/CSm_E_III_** | 0.612 | 0.501 | 0.987 | **1** |  |  |  |  |  |  |  |  |
| **5** | **ETo/CSm_E_III_** | 0.679 | 0.818 | 0.835 | 0.869 | **1** |  |  |  |  |  |  |  |
| **6** | **DIo/CSm_E_III_** | -0.272 | -0.173 | 0.598 | 0.464 | 0.273 | **1** |  |  |  |  |  |  |
| **7** | **RC/CSm_E_III_** | 0.529 | 0.755 | 0.792 | 0.806 | 0.850 | 0.350 | **1** |  |  |  |  |  |
| **8** | **Chla+b_E_III_** | 0.279 | 0.645 | 0.431 | 0.424 | 0.592 | 0.273 | 0.728 | **1** |  |  |  |  |
| **9** | **Car_E_III_** | 0.131 | 0.524 | 0.372 | 0.353 | 0.485 | 0.293 | 0.650 | 0.898 | **1** |  |  |  |
| **10** | **DWP_E_III_** | 0.051 | -0.080 | 0.165 | 0.146 | 0.099 | 0.183 | -0.027 | -0.134 | -0.090 | **1** |  |  |
| **11** | **GWE_E_III_** | 0.063 | -0.007 | 0.212 | 0.195 | 0.156 | 0.196 | 0.087 | 0.017 | 0.106 | 0.688 | **1** |  |
| **12** | **YP_E_III_** | 0.027 | -0.051 | 0.171 | 0.152 | 0.119 | 0.185 | 0.015 | -0.057 | 0.049 | 0.883 | 0.770 | **1** |

| Threshold | 0.203 | 0.264 | 0.334 | 0.384 |  | -0.203 | -0.264 | -0.334 | -0.384 |
| --- | --- | --- | --- | --- | --- | --- | --- | --- | --- |
| Positive | 0.050 | 0.010 | 0.001 | 0.0001 | Negative | 0.050 | 0.010 | 0.001 | 0.0001 |

*Colours and intensity of cell shading indicate significant positive (green) and negative (red) correlations*

*from P<0.05 to P<0.0001.*
