# Supplementary material for: Quantitative trait loci for leaf chlorophyll fluorescence parameters, chlorophyll and carotenoid contents in relation to biomass and yield in bread wheat and their chromosome deletion bin assignments
Source: Mol Breed. 2013 Apr 10;32(1):189–210. doi: 10.1007/s11032-013-9862-8 (PMC3684715; doi:10.1007/s11032-013-9862-8)
Supplement: Supplementary file 7 — Figure S1 Mean decade temperature (a) and humidity (b) during the four growing seasons (2007 - EI, 2008 – EII, 2010 – EIII and 2011 - EIV). The position of dashed and solid arrows is based on mean dates across the four years (EPS 1074 kb) (DOC 46 kb) [file 11032_2013_9862_MOESM7_ESM.doc]

**Quantitative trait loci for leaf chlorophyll fluorescence parameters, chlorophyll and carotenoid contents in relation to biomass and yield in bread wheat and their chromosome deletion bin assignments**

Czyczyło-Mysza I.^1^, Tyrka M.^2^, Marcińska I.^1^, Skrzypek E.^1^, Karbarz M.^3^, Dziurka M.^1^, Hura T.^1^, Dziurka K.^1^, Quarrie S.A.^4^

^1^ The *F. Górski* Institute of Plant Physiology, Polish Academy of Sciences, Kraków, Poland

^2^ Rzeszów University of Technology, Department of Biochemistry and Biotechnology, Poland.

^3^ Institute of Applied Biotechnology and Basic Sciences, University of Rzeszow

^4^ Faculty of Biology, Belgrade University, Serbia, and Visiting Professor, Newcastle University, UK.

Corresponding author: [czyczylo-mysza@wp.pl](javascript:oknoAdresat('napisz.html?to=czyczylo-mysza@wp.pl',10,10,650,540,1);)

measurement of FC, SPAD

and sample collection for

chlorophyll a, b

transfer of plants to pots

(stage: about 2-3 leaves)
