# Supplementary material for: Quantitative trait loci for leaf chlorophyll fluorescence parameters, chlorophyll and carotenoid contents in relation to biomass and yield in bread wheat and their chromosome deletion bin assignments
Source: Mol Breed. 2013 Apr 10;32(1):189–210. doi: 10.1007/s11032-013-9862-8 (PMC3684715; doi:10.1007/s11032-013-9862-8)
Supplement: Supplementary file 11 — Figure S3 Maps for chromosomes 1A to 7D, divided into the genetic map (left), the deletion bin map, break points and gene locations (middle) and CIM LOD and additive traces for traits (right). On the genetic map marker types are identified by colour: AFLPs - black, RFLPs - blue, SSRs - red, known-function markers - orange, DArTs - green. Best estimates of the location of chromosome centromeres are indicated with a black circle. DArT markers with previously-known chromosome assignments are identified with the chromosome assignment. To aid clarity, the prefix “X”, denoting markers of unknown function, is deleted from marker names. Markers that were assigned to specific deletion bins are identified with the fraction length for the bin, followed by a lower case letter indicating how the bin was identified (explained in Supplementary Table S1). C, S and L on bin fraction lengths indicate the chromosome centromere, short arm and long arm, respectively. On the middle map, chromosome break points and bin fraction lengths are selected and coloured according to Sourdille et al. (2004). White areas indicate uncertainty in the location of the break point. Centromeres are identified as a black line crossing the chromosome. Genes (explained in Supplementary Table S2) are located either in the predicted part of the bin or in the centre of the bin if a more precise location was not identified. Genes are coloured according to type of function: photosynthetic light reactions - blue, chlorophyll and carotenoid synthesis and metabolism - red, and biomass (carbohydrate) productivity - green, gene abbreviations explained in Supplementary Table S2. The right-hand map shows the CIM LOD output only for those traits giving a LOD maximum approaching a maximum of 1.8 or more. The dotted black line indicates a LOD score of 2.0. Underneath the LOD traces, the additive effects are shown as fractions of ±1 S.D. Additive effects show the direction of the QTL: positive where the Chinese Spring allel [file 11032_2013_9862_MOESM11_ESM.ppt]

## Slide 1
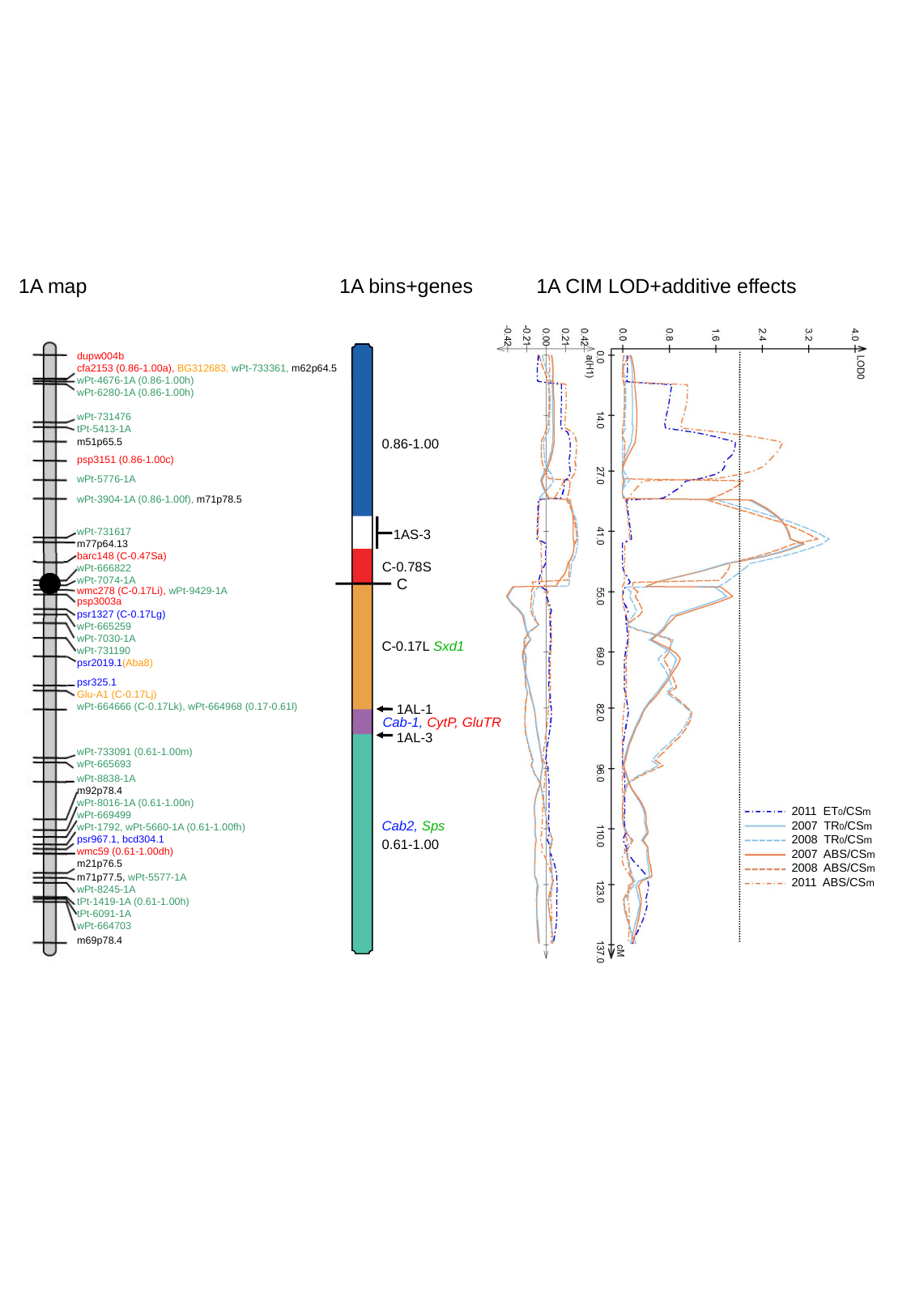

1A map
1A bins+genes
1A CIM LOD+additive effects
dupw004b
cfa2153 (0.86-1.00a), BG312683, wPt-733361, m62p64.5
wPt-4676-1A (0.86-1.00h)
wPt-6280-1A (0.86-1.00h)
wPt-731476
tPt-5413-1A
m51p65.5
psp3151 (0.86-1.00c)
wPt-5776-1A
wPt-3904-1A (0.86-1.00f), m71p78.5
wPt-731617
m77p64.13
barc148 (C-0.47Sa)
wPt-666822
wPt-7074-1A
wmc278 (C-0.17Li), wPt-9429-1A
psp3003a
psr1327 (C-0.17Lg)
wPt-665259
wPt-7030-1A
wPt-731190
psr2019.1(Aba8)
psr325.1
Glu-A1 (C-0.17Lj)
wPt-664666 (C-0.17Lk), wPt-664968 (0.17-0.61l)
wPt-733091 (0.61-1.00m)
wPt-665693
wPt-8838-1A
m92p78.4
wPt-8016-1A (0.61-1.00n)
wPt-669499
wPt-1792, wPt-5660-1A (0.61-1.00fh)
psr967.1, bcd304.1
wmc59 (0.61-1.00dh)
m21p76.5
m71p77.5, wPt-5577-1A
wPt-8245-1A
tPt-1419-1A (0.61-1.00h)
tPt-6091-1A
wPt-664703
m69p78.4
0.86-1.00
1AS-3
C-0.78S
C
C-0.17L Sxd1
1AL-1
Cab-1, CytP, GluTR
1AL-3
2011 ET0/CSm
2007 TR0/CSm
2008 TR0/CSm
2007 ABS/CSm
2008 ABS/CSm
2011 ABS/CSm
Cab2, Sps
0.61-1.00

## Slide 2
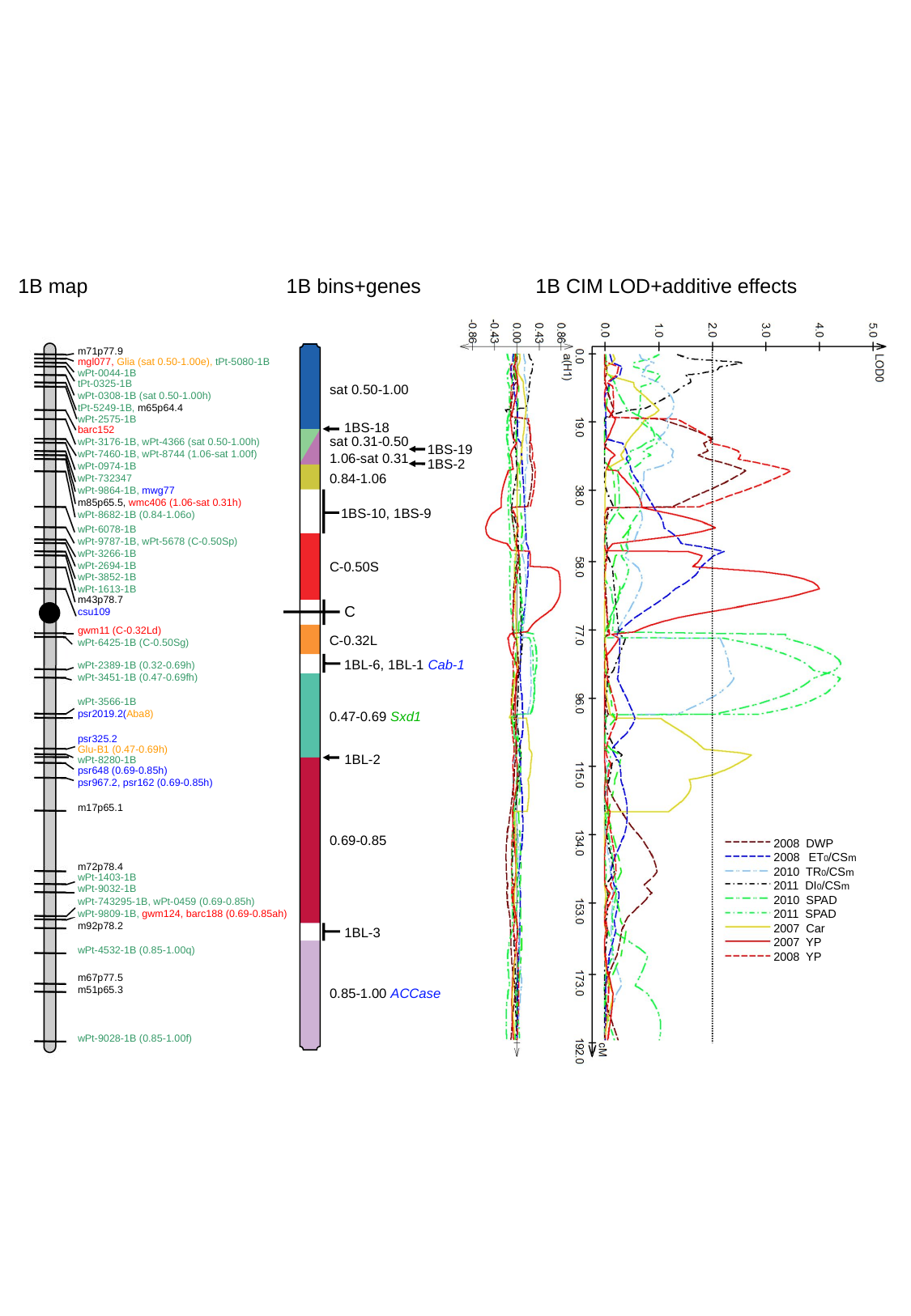

1B map
1B bins+genes
1B CIM LOD+additive effects
m71p77.9
mgl077, Glia (sat 0.50-1.00e), tPt-5080-1B
wPt-0044-1B
tPt-0325-1B
wPt-0308-1B (sat 0.50-1.00h)
tPt-5249-1B, m65p64.4
wPt-2575-1B
barc152
wPt-3176-1B, wPt-4366 (sat 0.50-1.00h)
wPt-7460-1B, wPt-8744 (1.06-sat 1.00f)
wPt-0974-1B
wPt-732347
wPt-9864-1B, mwg77
m85p65.5, wmc406 (1.06-sat 0.31h)
wPt-8682-1B (0.84-1.06o)
wPt-6078-1B
wPt-9787-1B, wPt-5678 (C-0.50Sp)
wPt-3266-1B
wPt-2694-1B
wPt-3852-1B
wPt-1613-1B
m43p78.7
csu109
gwm11 (C-0.32Ld)
wPt-6425-1B (C-0.50Sg)
wPt-2389-1B (0.32-0.69h)
wPt-3451-1B (0.47-0.69fh)
wPt-3566-1B
psr2019.2(Aba8)
psr325.2
Glu-B1 (0.47-0.69h)
wPt-8280-1B
psr648 (0.69-0.85h)
psr967.2, psr162 (0.69-0.85h)
m17p65.1
m72p78.4
wPt-1403-1B
wPt-9032-1B
wPt-743295-1B, wPt-0459 (0.69-0.85h)
wPt-9809-1B, gwm124, barc188 (0.69-0.85ah)
m92p78.2
wPt-4532-1B (0.85-1.00q)
m67p77.5
m51p65.3
wPt-9028-1B (0.85-1.00f)
sat 0.50-1.00
1BS-18
sat 0.31-0.50
1BS-19
1.06-sat 0.31
1BS-2
0.84-1.06
1BS-10, 1BS-9
C-0.50S
C
C-0.32L
1BL-6, 1BL-1 Cab-1
0.47-0.69 Sxd1
1BL-2
0.69-0.85
2008 DWP
 ET0/CSm
2010 TR0/CSm
2011 DI0/CSm
2010 SPAD
2011 SPAD
2007 Car
2007 YP
2008 YP
1BL-3
0.85-1.00 ACCase

## Slide 3
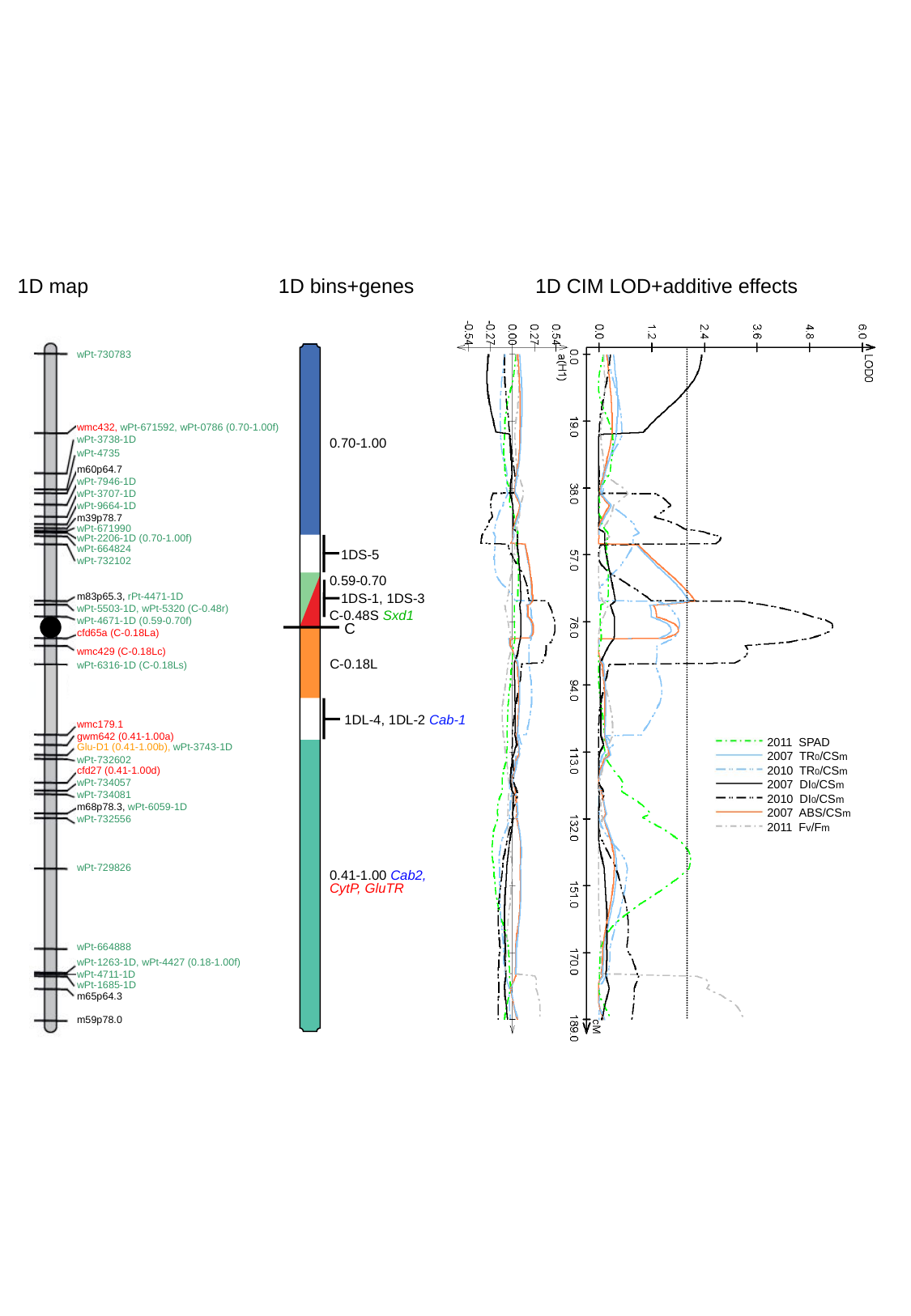

1D map
1D bins+genes
1D CIM LOD+additive effects
wPt-730783
wmc432, wPt-671592, wPt-0786 (0.70-1.00f)
wPt-3738-1D
wPt-4735
m60p64.7
wPt-7946-1D
wPt-3707-1D
wPt-9664-1D
m39p78.7
wPt-671990
wPt-2206-1D (0.70-1.00f)
wPt-664824
wPt-732102
m83p65.3, rPt-4471-1D
wPt-5503-1D, wPt-5320 (C-0.48r)
wPt-4671-1D (0.59-0.70f)
cfd65a (C-0.18La)
wmc429 (C-0.18Lc)
wPt-6316-1D (C-0.18Ls)
wmc179.1
gwm642 (0.41-1.00a)
Glu-D1 (0.41-1.00b), wPt-3743-1D
wPt-732602
cfd27 (0.41-1.00d)
wPt-734057
wPt-734081
m68p78.3, wPt-6059-1D
wPt-732556
wPt-729826
wPt-664888
wPt-1263-1D, wPt-4427 (0.18-1.00f)
wPt-4711-1D
wPt-1685-1D
m65p64.3
m59p78.0
0.70-1.00
1DS-5
0.59-0.70
1DS-1, 1DS-3
C-0.48S Sxd1
C
C-0.18L
1DL-4, 1DL-2 Cab-1
2011 SPAD
2007 TR0/CSm
2010 TR0/CSm
2007 DI0/CSm
2010 DI0/CSm
2007 ABS/CSm
2011 Fv/Fm
0.41-1.00 Cab2,
CytP, GluTR

## Slide 4
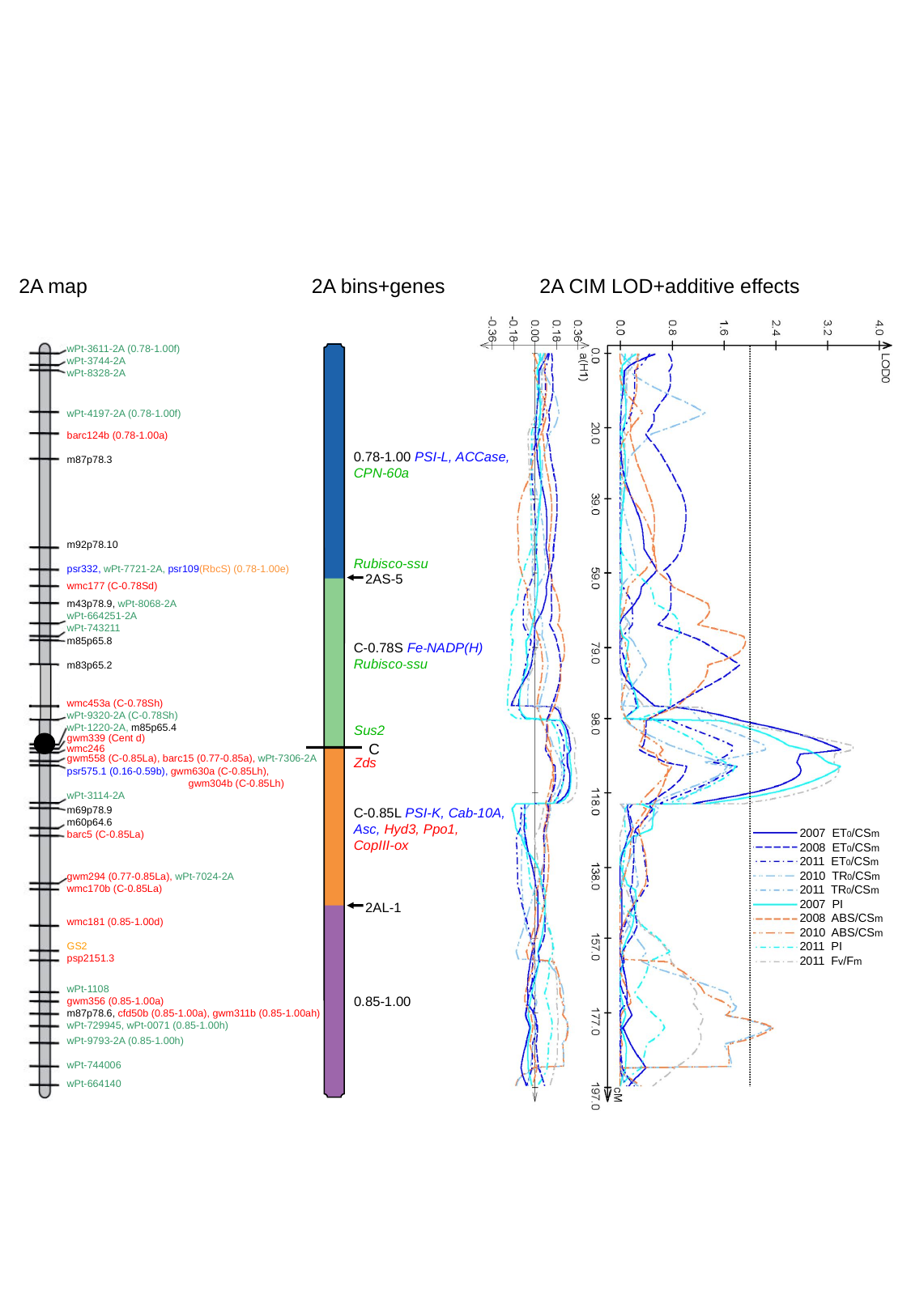

2A map
2A bins+genes
2A CIM LOD+additive effects
wPt-3611-2A (0.78-1.00f)
wPt-3744-2A
wPt-8328-2A
wPt-4197-2A (0.78-1.00f)
barc124b (0.78-1.00a)
m87p78.3
m92p78.10
psr332, wPt-7721-2A, psr109(RbcS) (0.78-1.00e)
wmc177 (C-0.78Sd)
m43p78.9, wPt-8068-2A
wPt-664251-2A
wPt-743211
m85p65.8
m83p65.2
wmc453a (C-0.78Sh)
wPt-9320-2A (C-0.78Sh)
wPt-1220-2A, m85p65.4
gwm339 (Cent d)
wmc246
gwm558 (C-0.85La), barc15 (0.77-0.85a), wPt-7306-2A
psr575.1 (0.16-0.59b), gwm630a (C-0.85Lh),
	gwm304b (C-0.85Lh)
wPt-3114-2A
m69p78.9
m60p64.6
barc5 (C-0.85La)
gwm294 (0.77-0.85La), wPt-7024-2A
wmc170b (C-0.85La)
wmc181 (0.85-1.00d)
GS2
psp2151.3
wPt-1108
gwm356 (0.85-1.00a)
m87p78.6, cfd50b (0.85-1.00a), gwm311b (0.85-1.00ah)
wPt-729945, wPt-0071 (0.85-1.00h)
wPt-9793-2A (0.85-1.00h)
wPt-744006
wPt-664140
0.78-1.00 PSI-L, ACCase,
CPN-60a
Rubisco-ssu
2AS-5
C-0.78S Fe-NADP(H)
Rubisco-ssu
Sus2
C
Zds
C-0.85L PSI-K, Cab-10A,
Asc, Hyd3, Ppo1,
CopIII-ox
2007 ET0/CSm
2008 ET0/CSm
2011 ET0/CSm
2010 TR0/CSm
2011 TR0/CSm
2007 PI
2008 ABS/CSm
2010 ABS/CSm
2011 PI
2011 Fv/Fm
2AL-1
0.85-1.00

## Slide 5
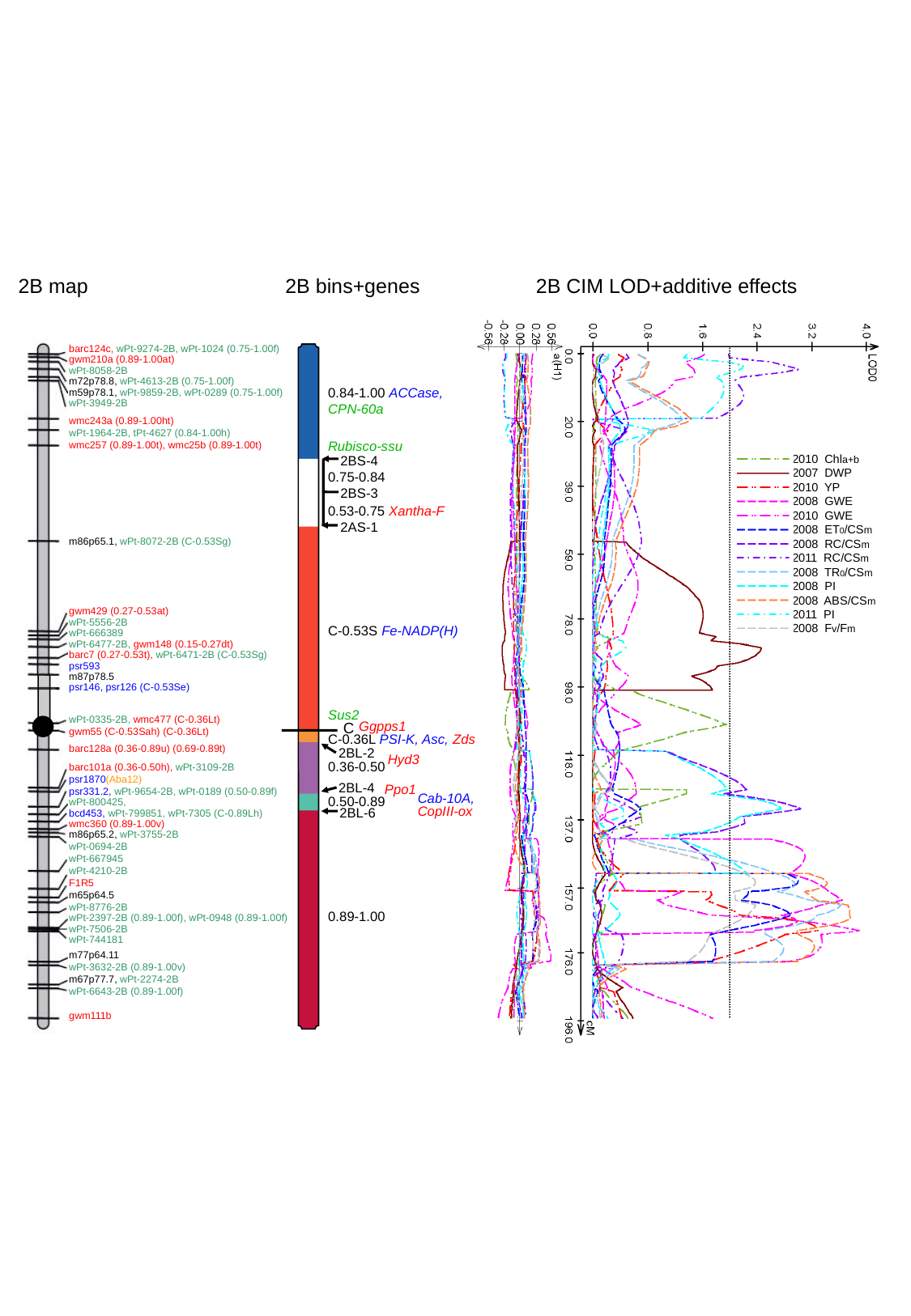

2B map
2B bins+genes
2B CIM LOD+additive effects
barc124c, wPt-9274-2B, wPt-1024 (0.75-1.00f)
gwm210a (0.89-1.00at)
wPt-8058-2B
m72p78.8, wPt-4613-2B (0.75-1.00f)
m59p78.1, wPt-9859-2B, wPt-0289 (0.75-1.00f)
wPt-3949-2B
wmc243a (0.89-1.00ht)
wPt-1964-2B, tPt-4627 (0.84-1.00h)
wmc257 (0.89-1.00t), wmc25b (0.89-1.00t)
m86p65.1, wPt-8072-2B (C-0.53Sg)
gwm429 (0.27-0.53at)
wPt-5556-2B
wPt-666389
wPt-6477-2B, gwm148 (0.15-0.27dt)
barc7 (0.27-0.53t), wPt-6471-2B (C-0.53Sg)
psr593
m87p78.5
psr146, psr126 (C-0.53Se)
wPt-0335-2B, wmc477 (C-0.36Lt)
gwm55 (C-0.53Sah) (C-0.36Lt)
barc128a (0.36-0.89u) (0.69-0.89t)
barc101a (0.36-0.50h), wPt-3109-2B
psr1870(Aba12)
psr331.2, wPt-9654-2B, wPt-0189 (0.50-0.89f)
wPt-800425,
bcd453, wPt-799851, wPt-7305 (C-0.89Lh)
wmc360 (0.89-1.00v)
m86p65.2, wPt-3755-2B
wPt-0694-2B
wPt-667945
wPt-4210-2B
F1R5
m65p64.5
wPt-8776-2B
wPt-2397-2B (0.89-1.00f), wPt-0948 (0.89-1.00f)
wPt-7506-2B
wPt-744181
m77p64.11
wPt-3632-2B (0.89-1.00v)
m67p77.7, wPt-2274-2B
wPt-6643-2B (0.89-1.00f)
gwm111b
0.84-1.00 ACCase,
CPN-60a
Rubisco-ssu
2010 Chla+b
2007 DWP
2010 YP
2008 GWE
2010 GWE
2008 ET0/CSm
2008 RC/CSm
2011 RC/CSm
2008 TR0/CSm
2008 PI
2008 ABS/CSm
2011 PI
2008 Fv/Fm
2BS-4
0.75-0.84
2BS-3
0.53-0.75 Xantha-F
2AS-1
C-0.53S Fe-NADP(H)
Sus2
Ggpps1
C
C-0.36L PSI-K, Asc, Zds
2BL-2
Hyd3
0.36-0.50
2BL-4
Ppo1
0.50-0.89
Cab-10A,
CopIII-ox
2BL-6
0.89-1.00

## Slide 6
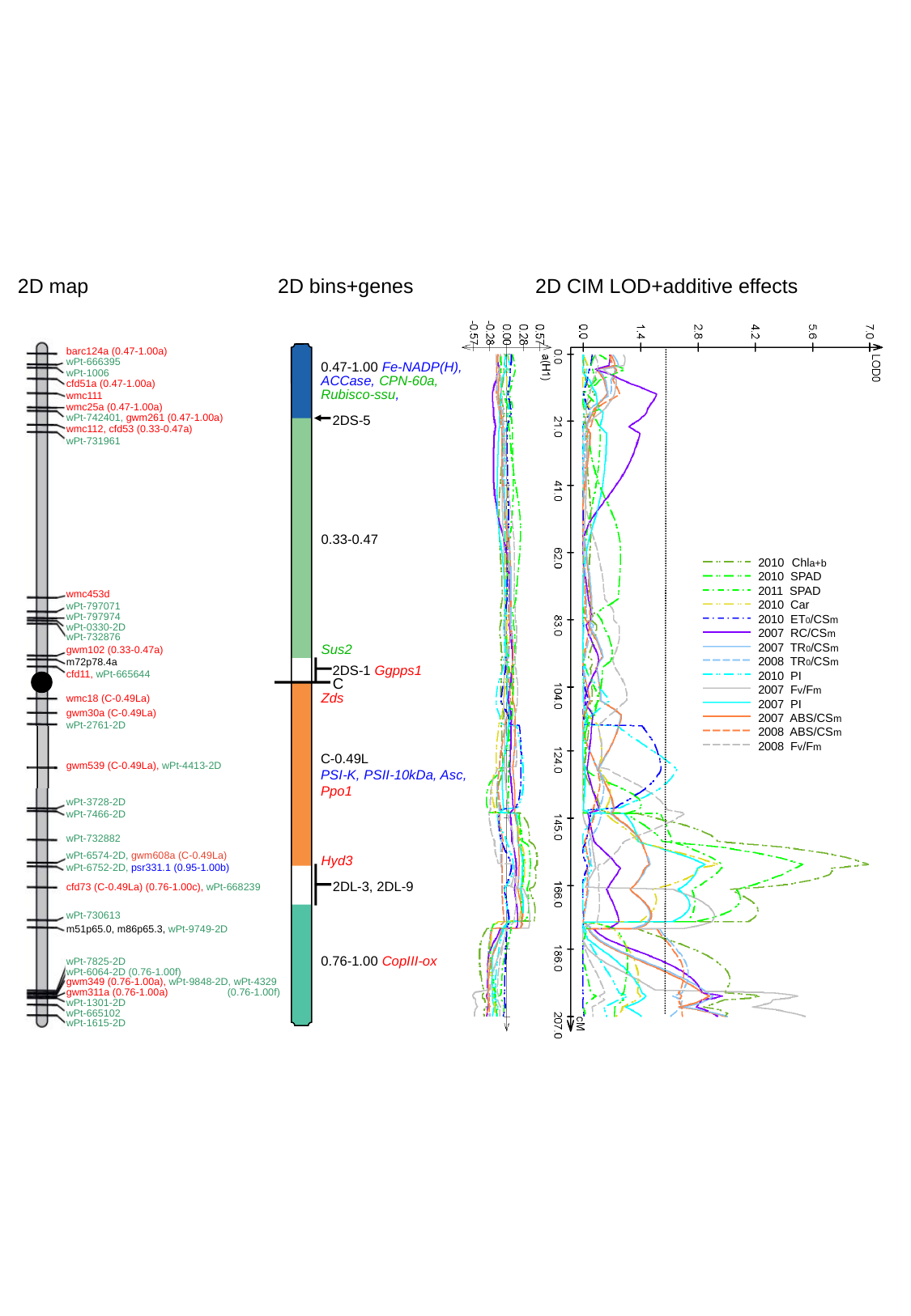

2D map
2D bins+genes
2D CIM LOD+additive effects
barc124a (0.47-1.00a)
wPt-666395
wPt-1006
cfd51a (0.47-1.00a)
wmc111
wmc25a (0.47-1.00a)
wPt-742401, gwm261 (0.47-1.00a)
wmc112, cfd53 (0.33-0.47a)
wPt-731961
wmc453d
wPt-797071
wPt-797974
wPt-0330-2D
wPt-732876
gwm102 (0.33-0.47a)
m72p78.4a
cfd11, wPt-665644
wmc18 (C-0.49La)
gwm30a (C-0.49La)
wPt-2761-2D
gwm539 (C-0.49La), wPt-4413-2D
wPt-3728-2D
wPt-7466-2D
wPt-732882
wPt-6574-2D, gwm608a (C-0.49La)
wPt-6752-2D, psr331.1 (0.95-1.00b)
cfd73 (C-0.49La) (0.76-1.00c), wPt-668239
wPt-730613
m51p65.0, m86p65.3, wPt-9749-2D
wPt-7825-2D
wPt-6064-2D (0.76-1.00f)
gwm349 (0.76-1.00a), wPt-9848-2D, wPt-4329
gwm311a (0.76-1.00a)	 (0.76-1.00f)
wPt-1301-2D
wPt-665102
wPt-1615-2D
0.47-1.00 Fe-NADP(H),
ACCase, CPN-60a,
Rubisco-ssu,
2DS-5
0.33-0.47
 Chla+b
2010 SPAD
2011 SPAD
2010 Car
2010 ET0/CSm
2007 RC/CSm
2007 TR0/CSm
2008 TR0/CSm
2010 PI
2007 Fv/Fm
2007 PI
2007 ABS/CSm
2008 ABS/CSm
2008 Fv/Fm
Sus2
2DS-1 Ggpps1
C
Zds
C-0.49L
PSI-K, PSII-10kDa, Asc,
Ppo1
Hyd3
2DL-3, 2DL-9
0.76-1.00 CopIII-ox

## Slide 7
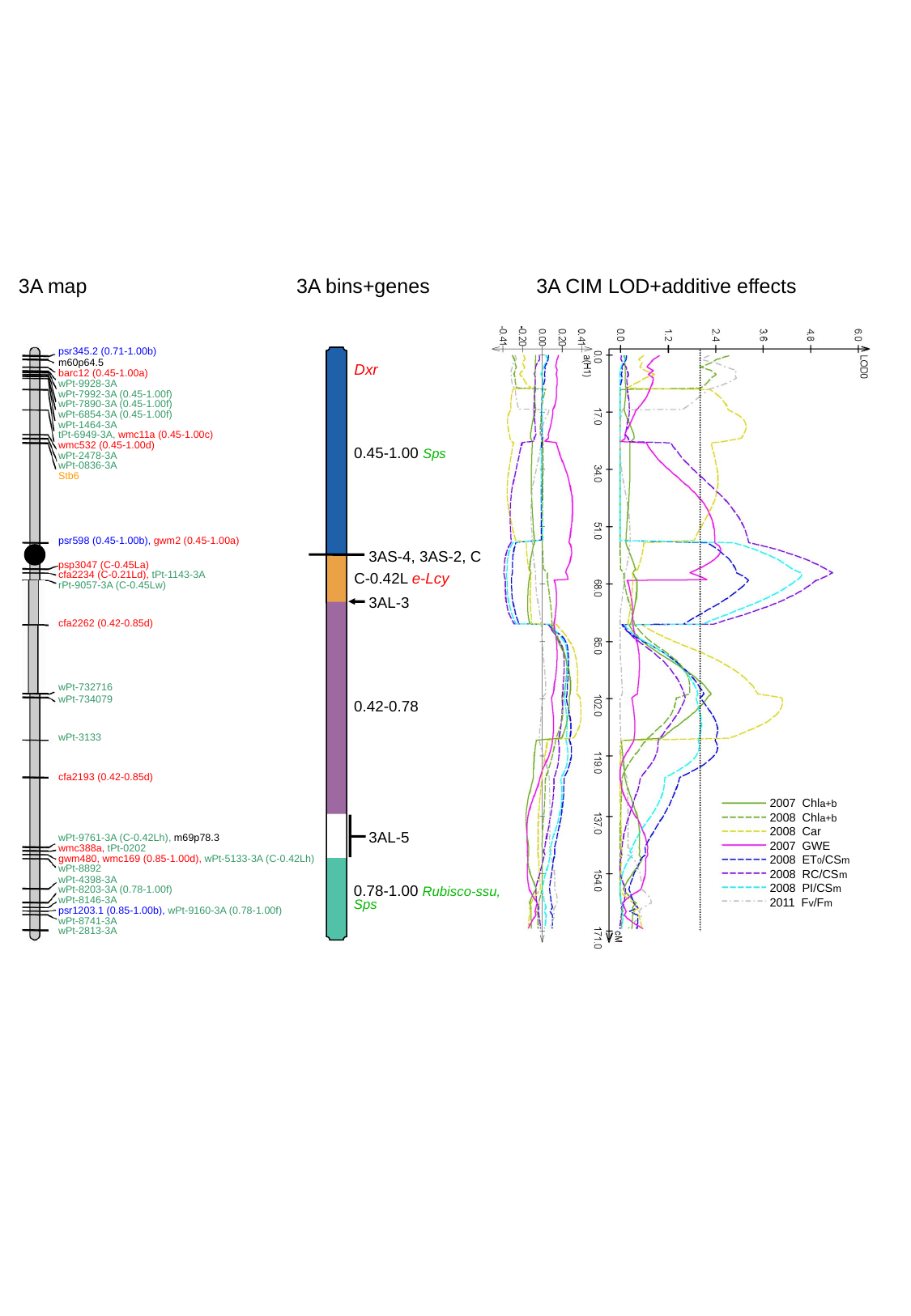

3A map
3A bins+genes
3A CIM LOD+additive effects
psr345.2 (0.71-1.00b)
m60p64.5
barc12 (0.45-1.00a)
wPt-9928-3A
wPt-7992-3A (0.45-1.00f)
wPt-7890-3A (0.45-1.00f)
wPt-6854-3A (0.45-1.00f)
wPt-1464-3A
tPt-6949-3A, wmc11a (0.45-1.00c)
wmc532 (0.45-1.00d)
wPt-2478-3A
wPt-0836-3A
Stb6
psr598 (0.45-1.00b), gwm2 (0.45-1.00a)
psp3047 (C-0.45La)
cfa2234 (C-0.21Ld), tPt-1143-3A
rPt-9057-3A (C-0.45Lw)
cfa2262 (0.42-0.85d)
wPt-732716
wPt-734079
wPt-3133
cfa2193 (0.42-0.85d)
wPt-9761-3A (C-0.42Lh), m69p78.3
wmc388a, tPt-0202
gwm480, wmc169 (0.85-1.00d), wPt-5133-3A (C-0.42Lh)
wPt-8892
wPt-4398-3A
wPt-8203-3A (0.78-1.00f)
wPt-8146-3A
psr1203.1 (0.85-1.00b), wPt-9160-3A (0.78-1.00f)
wPt-8741-3A
wPt-2813-3A
Dxr
0.45-1.00 Sps
3AS-4, 3AS-2, C
C-0.42L e-Lcy
3AL-3
0.42-0.78
2007 Chla+b
2008 Chla+b
2008 Car
2007 GWE
2008 ET0/CSm
2008 RC/CSm
2008 PI/CSm
2011 Fv/Fm
3AL-5
0.78-1.00 Rubisco-ssu,
Sps

## Slide 8
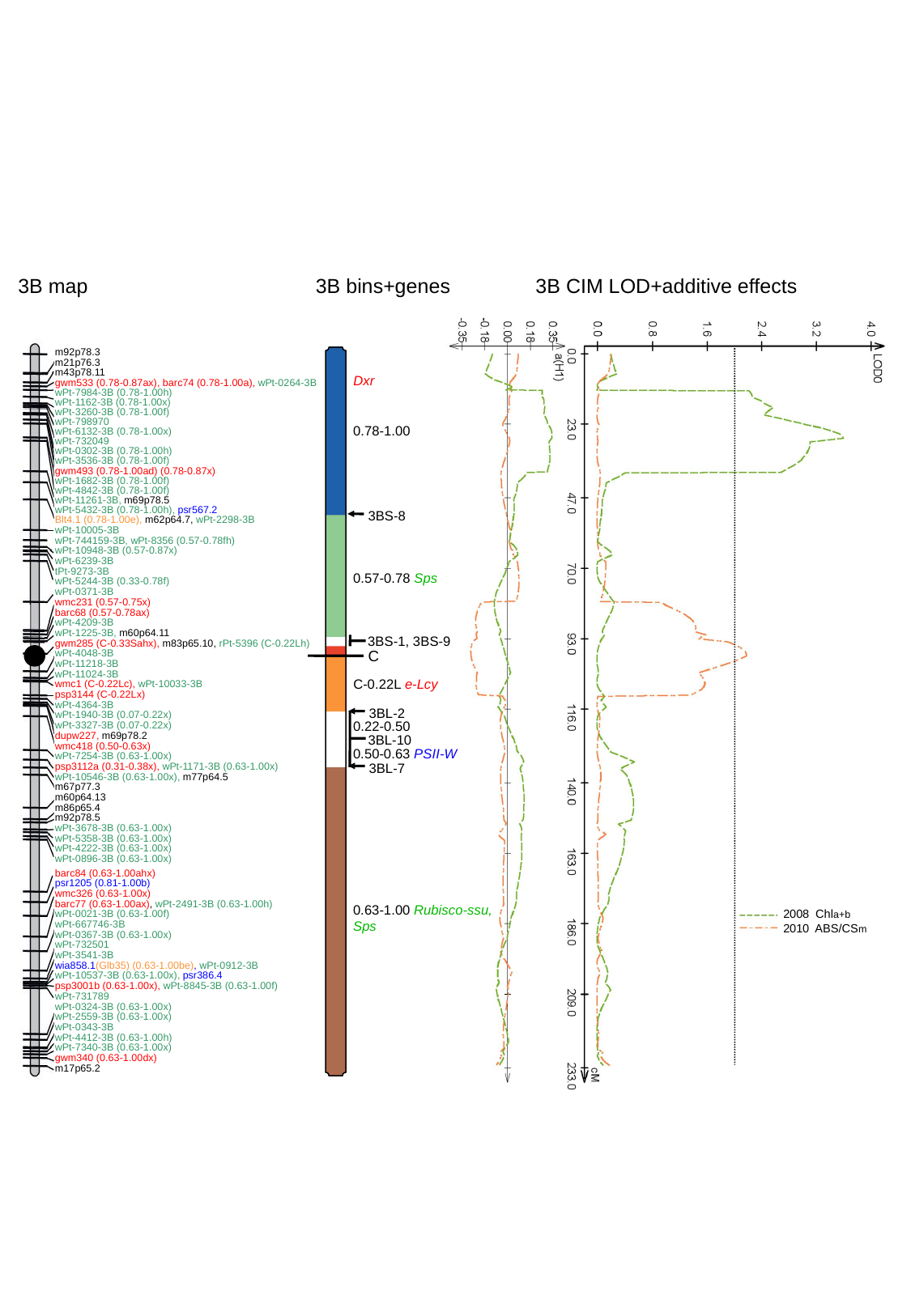

3B map
3B bins+genes
3B CIM LOD+additive effects
m92p78.3
m21p76.3
m43p78.11
gwm533 (0.78-0.87ax), barc74 (0.78-1.00a), wPt-0264-3B
wPt-7984-3B (0.78-1.00h)
wPt-1162-3B (0.78-1.00x)
wPt-3260-3B (0.78-1.00f)
wPt-798970
wPt-6132-3B (0.78-1.00x)
wPt-732049
wPt-0302-3B (0.78-1.00h)
wPt-3536-3B (0.78-1.00f)
gwm493 (0.78-1.00ad) (0.78-0.87x)
wPt-1682-3B (0.78-1.00f)
wPt-4842-3B (0.78-1.00f)
wPt-11261-3B, m69p78.5
wPt-5432-3B (0.78-1.00h), psr567.2
Blt4.1 (0.78-1.00e), m62p64.7, wPt-2298-3B
wPt-10005-3B
wPt-744159-3B, wPt-8356 (0.57-0.78fh)
wPt-10948-3B (0.57-0.87x)
wPt-6239-3B
tPt-9273-3B
wPt-5244-3B (0.33-0.78f)
wPt-0371-3B
wmc231 (0.57-0.75x)
barc68 (0.57-0.78ax)
wPt-4209-3B
wPt-1225-3B, m60p64.11
gwm285 (C-0.33Sahx), m83p65.10, rPt-5396 (C-0.22Lh)
wPt-4048-3B
wPt-11218-3B
wPt-11024-3B
wmc1 (C-0.22Lc), wPt-10033-3B
psp3144 (C-0.22Lx)
wPt-4364-3B
wPt-1940-3B (0.07-0.22x)
wPt-3327-3B (0.07-0.22x)
dupw227, m69p78.2
wmc418 (0.50-0.63x)
wPt-7254-3B (0.63-1.00x)
psp3112a (0.31-0.38x), wPt-1171-3B (0.63-1.00x)
wPt-10546-3B (0.63-1.00x), m77p64.5
m67p77.3
m60p64.13
m86p65.4
m92p78.5
wPt-3678-3B (0.63-1.00x)
wPt-5358-3B (0.63-1.00x)
wPt-4222-3B (0.63-1.00x)
wPt-0896-3B (0.63-1.00x)
barc84 (0.63-1.00ahx)
psr1205 (0.81-1.00b)
wmc326 (0.63-1.00x)
barc77 (0.63-1.00ax), wPt-2491-3B (0.63-1.00h)
wPt-0021-3B (0.63-1.00f)
wPt-667746-3B
wPt-0367-3B (0.63-1.00x)
wPt-732501
wPt-3541-3B
wia858.1(Glb35) (0.63-1.00be), wPt-0912-3B
wPt-10537-3B (0.63-1.00x), psr386.4
psp3001b (0.63-1.00x), wPt-8845-3B (0.63-1.00f)
wPt-731789
wPt-0324-3B (0.63-1.00x)
wPt-2559-3B (0.63-1.00x)
wPt-0343-3B
wPt-4412-3B (0.63-1.00h)
wPt-7340-3B (0.63-1.00x)
gwm340 (0.63-1.00dx)
m17p65.2
Dxr
0.78-1.00
3BS-8
0.57-0.78 Sps
3BS-1, 3BS-9
C
C-0.22L e-Lcy
3BL-2
0.22-0.50
3BL-10
0.50-0.63 PSII-W
3BL-7
0.63-1.00 Rubisco-ssu,
Sps
2008 Chla+b
2010 ABS/CSm

## Slide 9
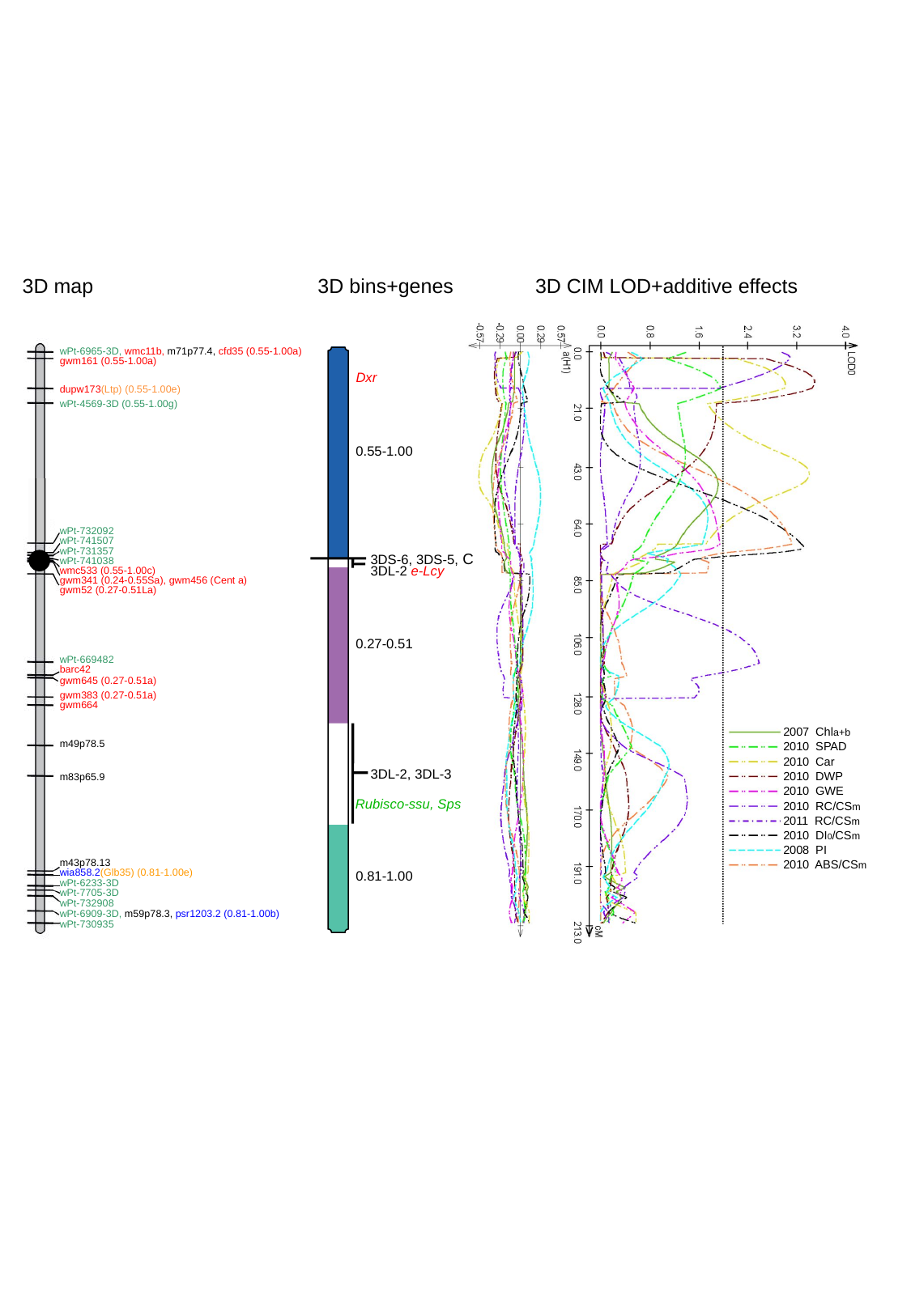

3D map
3D bins+genes
3D CIM LOD+additive effects
wPt-6965-3D, wmc11b, m71p77.4, cfd35 (0.55-1.00a)
gwm161 (0.55-1.00a)
dupw173(Ltp) (0.55-1.00e)
wPt-4569-3D (0.55-1.00g)
wPt-732092
wPt-741507
wPt-731357
wPt-741038
wmc533 (0.55-1.00c)
gwm341 (0.24-0.55Sa), gwm456 (Cent a)
gwm52 (0.27-0.51La)
wPt-669482
barc42
gwm645 (0.27-0.51a)
gwm383 (0.27-0.51a)
gwm664
m49p78.5
m83p65.9
m43p78.13
wia858.2(Glb35) (0.81-1.00e)
wPt-6233-3D
wPt-7705-3D
wPt-732908
wPt-6909-3D, m59p78.3, psr1203.2 (0.81-1.00b)
wPt-730935
Dxr
0.55-1.00
3DS-6, 3DS-5, C
3DL-2 e-Lcy
0.27-0.51
2007 Chla+b
2010 SPAD
2010 Car
2010 DWP
2010 GWE
2010 RC/CSm
2011 RC/CSm
2010 DI0/CSm
2008 PI
2010 ABS/CSm
3DL-2, 3DL-3
Rubisco-ssu, Sps
0.81-1.00

## Slide 10
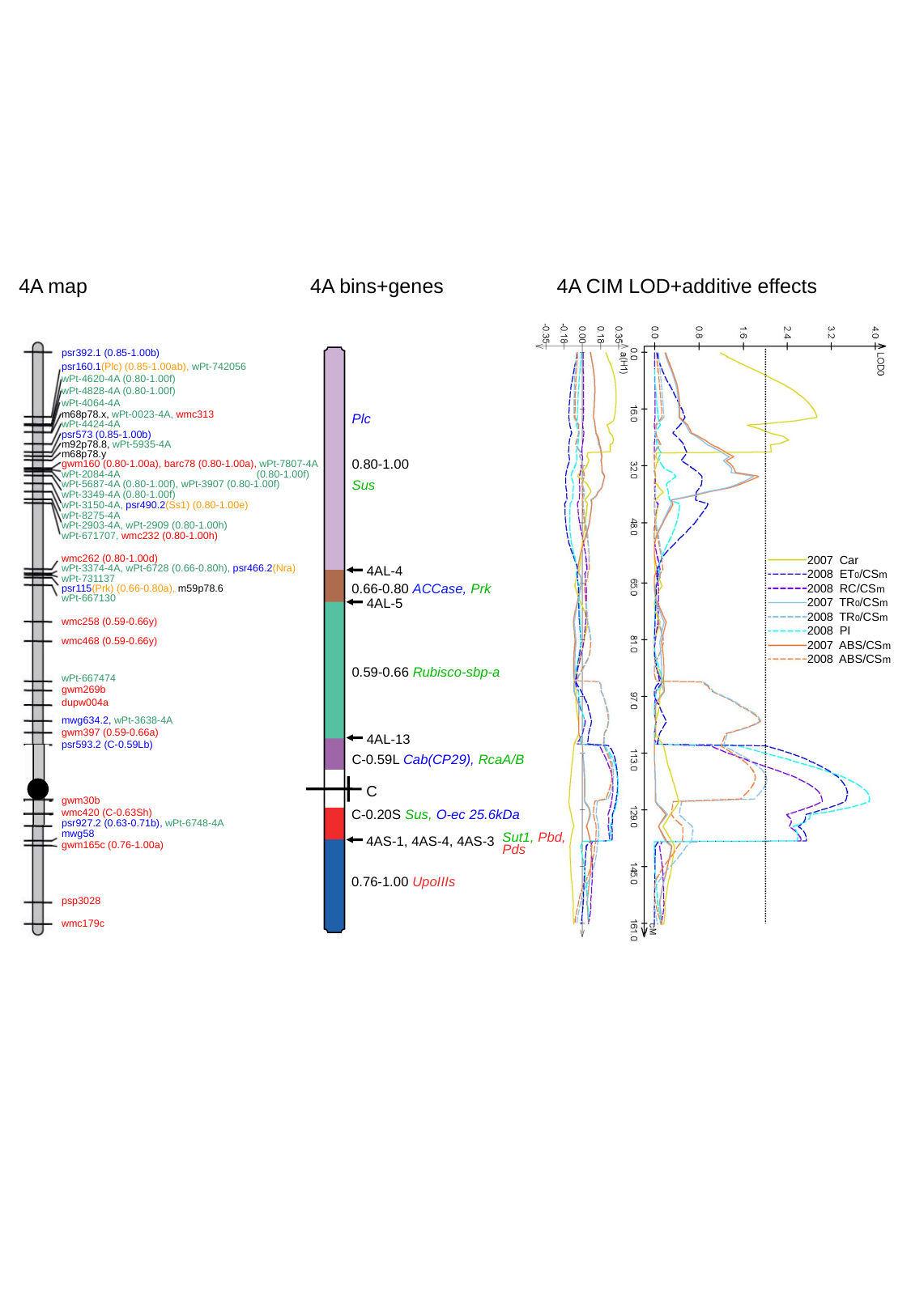

4A map
4A bins+genes
4A CIM LOD+additive effects
psr392.1 (0.85-1.00b)
psr160.1(Plc) (0.85-1.00ab), wPt-742056
wPt-4620-4A (0.80-1.00f)
wPt-4828-4A (0.80-1.00f)
wPt-4064-4A
m68p78.x, wPt-0023-4A, wmc313
wPt-4424-4A
psr573 (0.85-1.00b)
m92p78.8, wPt-5935-4A
m68p78.y
gwm160 (0.80-1.00a), barc78 (0.80-1.00a), wPt-7807-4A
wPt-2084-4A	 (0.80-1.00f)
wPt-5687-4A (0.80-1.00f), wPt-3907 (0.80-1.00f)
wPt-3349-4A (0.80-1.00f)
wPt-3150-4A, psr490.2(Ss1) (0.80-1.00e)
wPt-8275-4A
wPt-2903-4A, wPt-2909 (0.80-1.00h)
wPt-671707, wmc232 (0.80-1.00h)
wmc262 (0.80-1.00d)
wPt-3374-4A, wPt-6728 (0.66-0.80h), psr466.2(Nra)
wPt-731137
psr115(Prk) (0.66-0.80a), m59p78.6
wPt-667130
wmc258 (0.59-0.66y)
wmc468 (0.59-0.66y)
wPt-667474
gwm269b
dupw004a
mwg634.2, wPt-3638-4A
gwm397 (0.59-0.66a)
psr593.2 (C-0.59Lb)
gwm30b
wmc420 (C-0.63Sh)
psr927.2 (0.63-0.71b), wPt-6748-4A
mwg58
gwm165c (0.76-1.00a)
psp3028
wmc179c
Plc
0.80-1.00
Sus
2007 Car
2008 ET0/CSm
2008 RC/CSm
2007 TR0/CSm
2008 TR0/CSm
2008 PI
2007 ABS/CSm
2008 ABS/CSm
4AL-4
0.66-0.80 ACCase, Prk
4AL-5
0.59-0.66 Rubisco-sbp-a
4AL-13
C-0.59L Cab(CP29), RcaA/B
C
C-0.20S Sus, O-ec 25.6kDa
Sut1, Pbd,
Pds
4AS-1, 4AS-4, 4AS-3
0.76-1.00 UpoIIIs

## Slide 11
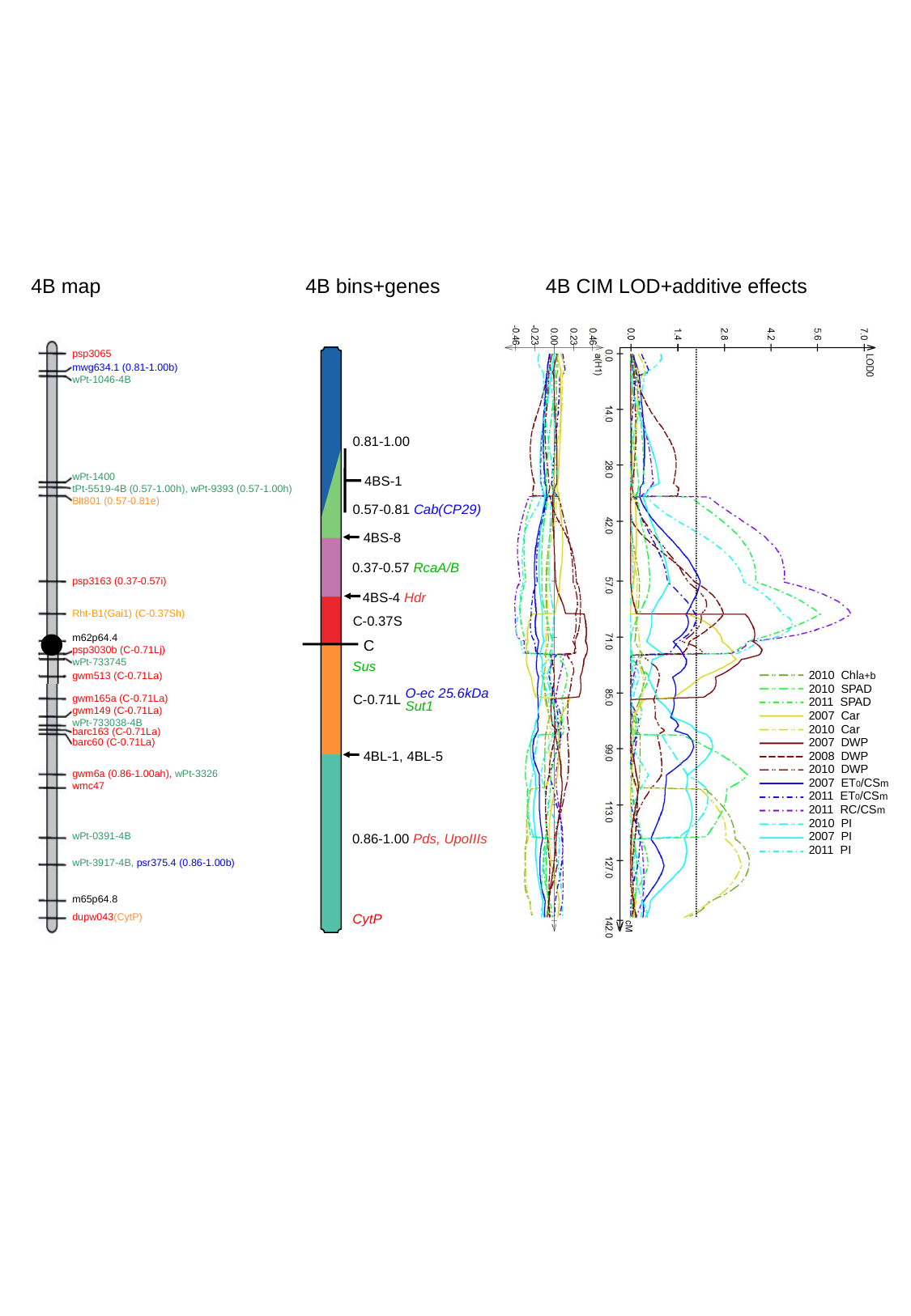

4B map
4B bins+genes
4B CIM LOD+additive effects
psp3065
mwg634.1 (0.81-1.00b)
wPt-1046-4B
wPt-1400
tPt-5519-4B (0.57-1.00h), wPt-9393 (0.57-1.00h)
Blt801 (0.57-0.81e)
psp3163 (0.37-0.57i)
Rht-B1(Gai1) (C-0.37Sh)
m62p64.4
psp3030b (C-0.71Lj)
wPt-733745
gwm513 (C-0.71La)
gwm165a (C-0.71La)
gwm149 (C-0.71La)
wPt-733038-4B
barc163 (C-0.71La)
barc60 (C-0.71La)
gwm6a (0.86-1.00ah), wPt-3326
wmc47
wPt-0391-4B
wPt-3917-4B, psr375.4 (0.86-1.00b)
m65p64.8
dupw043(CytP)
0.81-1.00
4BS-1
0.57-0.81 Cab(CP29)
4BS-8
0.37-0.57 RcaA/B
4BS-4 Hdr
C-0.37S
C
Sus
2010 Chla+b
2010 SPAD
2011 SPAD
2007 Car
2010 Car
2007 DWP
2008 DWP
2010 DWP
2007 ET0/CSm
2011 ET0/CSm
2011 RC/CSm
2010 PI
2007 PI
2011 PI
O-ec 25.6kDa
Sut1
C-0.71L
4BL-1, 4BL-5
0.86-1.00 Pds, UpoIIIs
CytP

## Slide 12
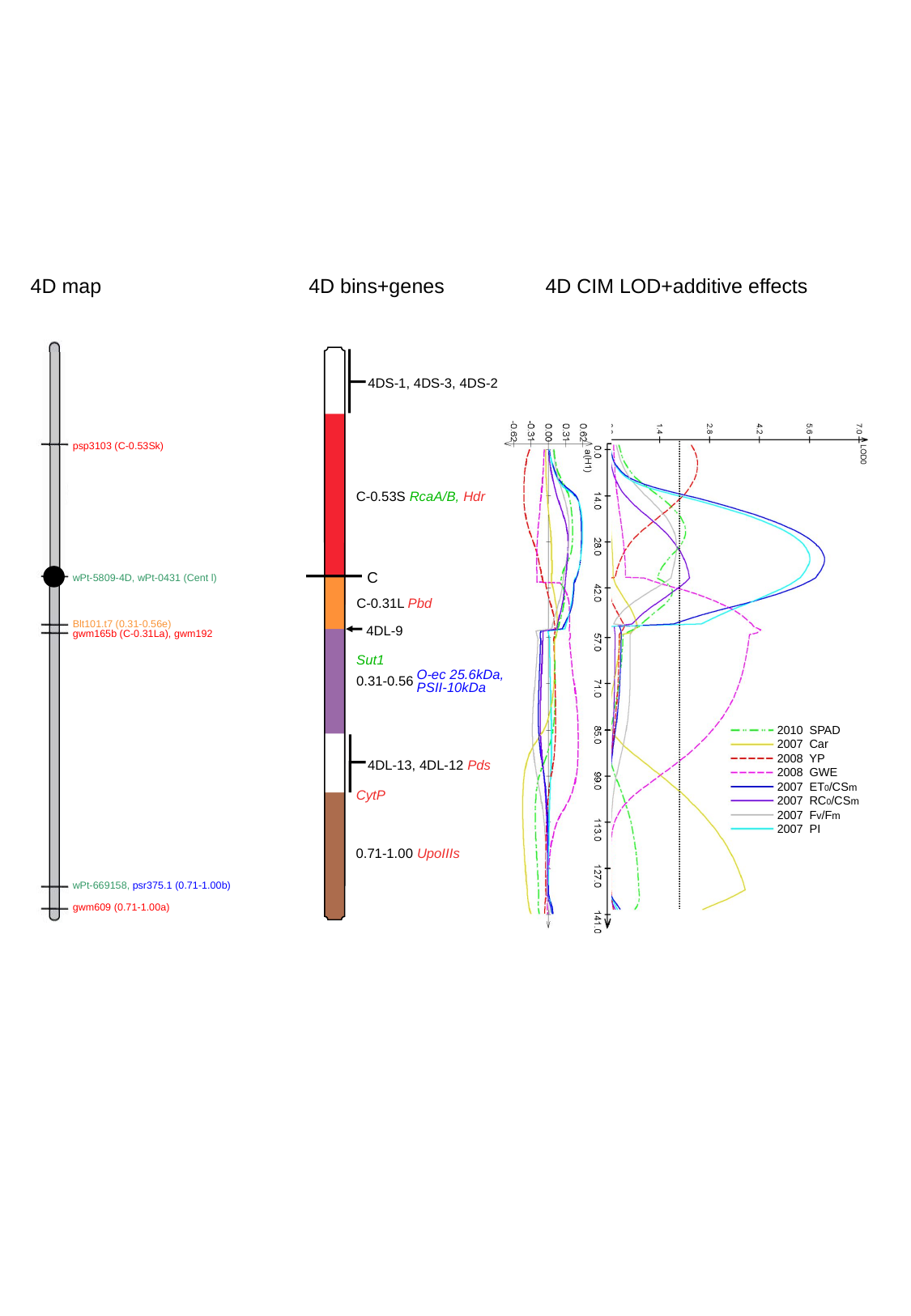

4D map
4D bins+genes
4D CIM LOD+additive effects
4DS-1, 4DS-3, 4DS-2
psp3103 (C-0.53Sk)
wPt-5809-4D, wPt-0431 (Cent l)
Blt101.t7 (0.31-0.56e)
gwm165b (C-0.31La), gwm192
wPt-669158, psr375.1 (0.71-1.00b)
gwm609 (0.71-1.00a)
C-0.53S RcaA/B, Hdr
C
C-0.31L Pbd
4DL-9
Sut1
O-ec 25.6kDa,
PSII-10kDa
0.31-0.56
2010 SPAD
2007 Car
2008 YP
2008 GWE
2007 ET0/CSm
2007 RC0/CSm
2007 Fv/Fm
2007 PI
4DL-13, 4DL-12 Pds
CytP
0.71-1.00 UpoIIIs

## Slide 13
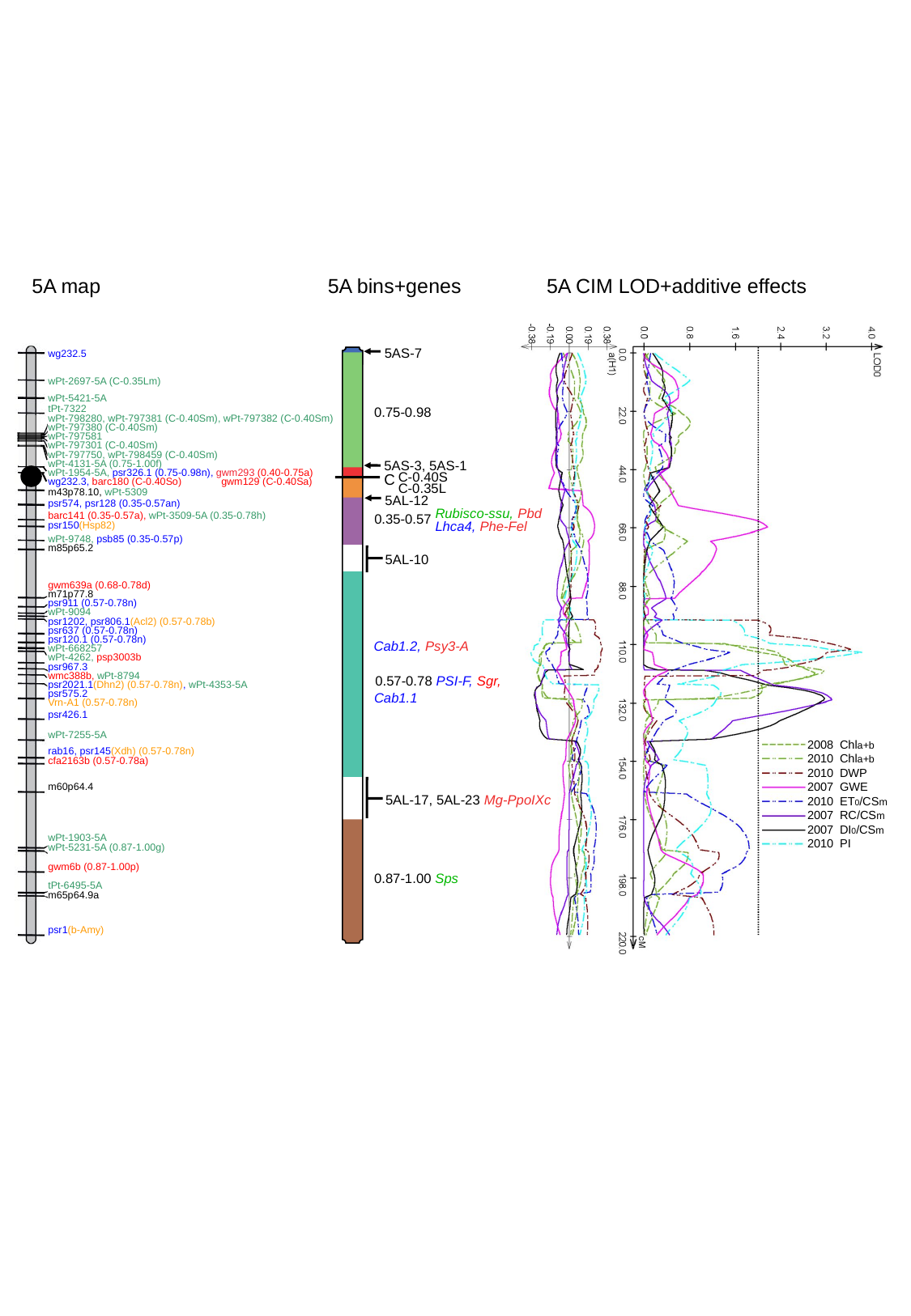

5A map
5A bins+genes
5A CIM LOD+additive effects
5AS-7
wg232.5
wPt-2697-5A (C-0.35Lm)
wPt-5421-5A
tPt-7322
wPt-798280, wPt-797381 (C-0.40Sm), wPt-797382 (C-0.40Sm)
wPt-797380 (C-0.40Sm)
wPt-797581
wPt-797301 (C-0.40Sm)
wPt-797750, wPt-798459 (C-0.40Sm)
wPt-4131-5A (0.75-1.00f)
wPt-1954-5A, psr326.1 (0.75-0.98n), gwm293 (0.40-0.75a)
wg232.3, barc180 (C-0.40So) gwm129 (C-0.40Sa)
m43p78.10, wPt-5309
psr574, psr128 (0.35-0.57an)
barc141 (0.35-0.57a), wPt-3509-5A (0.35-0.78h)
psr150(Hsp82)
wPt-9748, psb85 (0.35-0.57p)
m85p65.2
gwm639a (0.68-0.78d)
m71p77.8
psr911 (0.57-0.78n)
wPt-9094
psr1202, psr806.1(Acl2) (0.57-0.78b)
psr637 (0.57-0.78n)
psr120.1 (0.57-0.78n)
wPt-668257
wPt-4262, psp3003b
psr967.3
wmc388b, wPt-8794
psr2021.1(Dhn2) (0.57-0.78n), wPt-4353-5A
psr575.2
Vrn-A1 (0.57-0.78n)
psr426.1
wPt-7255-5A
rab16, psr145(Xdh) (0.57-0.78n)
cfa2163b (0.57-0.78a)
m60p64.4
wPt-1903-5A
wPt-5231-5A (0.87-1.00g)
gwm6b (0.87-1.00p)
tPt-6495-5A
m65p64.9a
psr1(b-Amy)
0.75-0.98
5AS-3, 5AS-1
C-0.40S
C
C-0.35L
5AL-12
Rubisco-ssu, Pbd
Lhca4, Phe-Fel
0.35-0.57
5AL-10
Cab1.2, Psy3-A
0.57-0.78 PSI-F, Sgr,
Cab1.1
2008 Chla+b
2010 Chla+b
2010 DWP
2007 GWE
2010 ET0/CSm
2007 RC/CSm
2007 DI0/CSm
2010 PI
5AL-17, 5AL-23 Mg-PpoIXc
0.87-1.00 Sps

## Slide 14
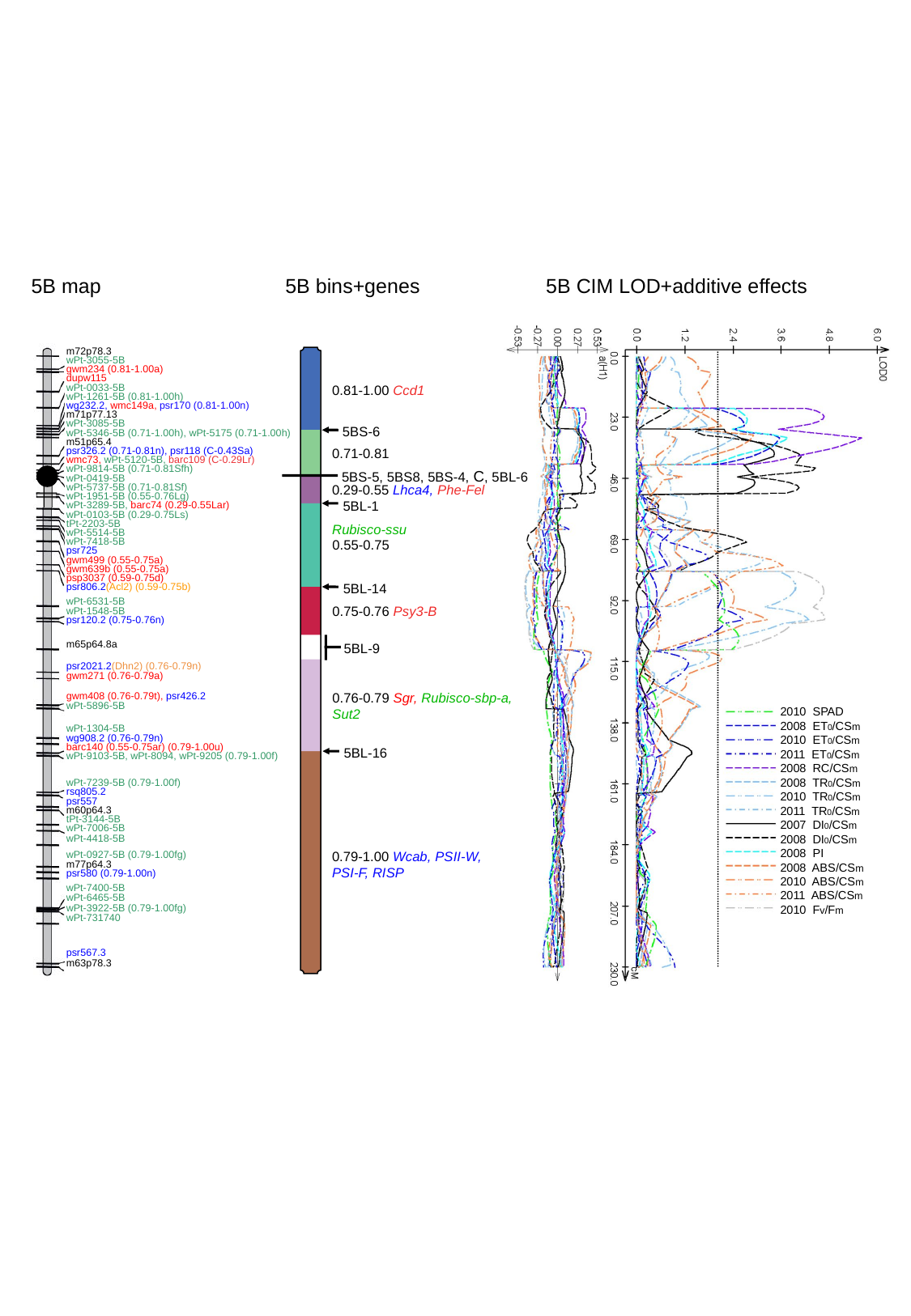

5B map
5B bins+genes
5B CIM LOD+additive effects
m72p78.3
wPt-3055-5B
gwm234 (0.81-1.00a)
dupw115
wPt-0033-5B
wPt-1261-5B (0.81-1.00h)
wg232.2, wmc149a, psr170 (0.81-1.00n)
m71p77.13
wPt-3085-5B
wPt-5346-5B (0.71-1.00h), wPt-5175 (0.71-1.00h)
m51p65.4
psr326.2 (0.71-0.81n), psr118 (C-0.43Sa)
wmc73, wPt-5120-5B, barc109 (C-0.29Lr)
wPt-9814-5B (0.71-0.81Sfh)
wPt-0419-5B
wPt-5737-5B (0.71-0.81Sf)
wPt-1951-5B (0.55-0.76Lg)
wPt-3289-5B, barc74 (0.29-0.55Lar)
wPt-0103-5B (0.29-0.75Ls)
tPt-2203-5B
wPt-5514-5B
wPt-7418-5B
psr725
gwm499 (0.55-0.75a)
gwm639b (0.55-0.75a)
psp3037 (0.59-0.75d)
psr806.2(Acl2) (0.59-0.75b)
wPt-6531-5B
wPt-1548-5B
psr120.2 (0.75-0.76n)
m65p64.8a
psr2021.2(Dhn2) (0.76-0.79n)
gwm271 (0.76-0.79a)
gwm408 (0.76-0.79t), psr426.2
wPt-5896-5B
wPt-1304-5B
wg908.2 (0.76-0.79n)
barc140 (0.55-0.75ar) (0.79-1.00u)
wPt-9103-5B, wPt-8094, wPt-9205 (0.79-1.00f)
wPt-7239-5B (0.79-1.00f)
rsq805.2
psr557
m60p64.3
tPt-3144-5B
wPt-7006-5B
wPt-4418-5B
wPt-0927-5B (0.79-1.00fg)
m77p64.3
psr580 (0.79-1.00n)
wPt-7400-5B
wPt-6465-5B
wPt-3922-5B (0.79-1.00fg)
wPt-731740
psr567.3
m63p78.3
0.81-1.00 Ccd1
5BS-6
0.71-0.81
5BS-5, 5BS8, 5BS-4, C, 5BL-6
0.29-0.55 Lhca4, Phe-Fel
5BL-1
Rubisco-ssu
0.55-0.75
5BL-14
0.75-0.76 Psy3-B
5BL-9
0.76-0.79 Sgr, Rubisco-sbp-a,
Sut2
2010 SPAD
2008 ET0/CSm
2010 ET0/CSm
2011 ET0/CSm
2008 RC/CSm
2008 TR0/CSm
2010 TR0/CSm
2011 TR0/CSm
2007 DI0/CSm
2008 DI0/CSm
2008 PI
2008 ABS/CSm
2010 ABS/CSm
2011 ABS/CSm
2010 Fv/Fm
5BL-16
0.79-1.00 Wcab, PSII-W,
PSI-F, RISP

## Slide 15
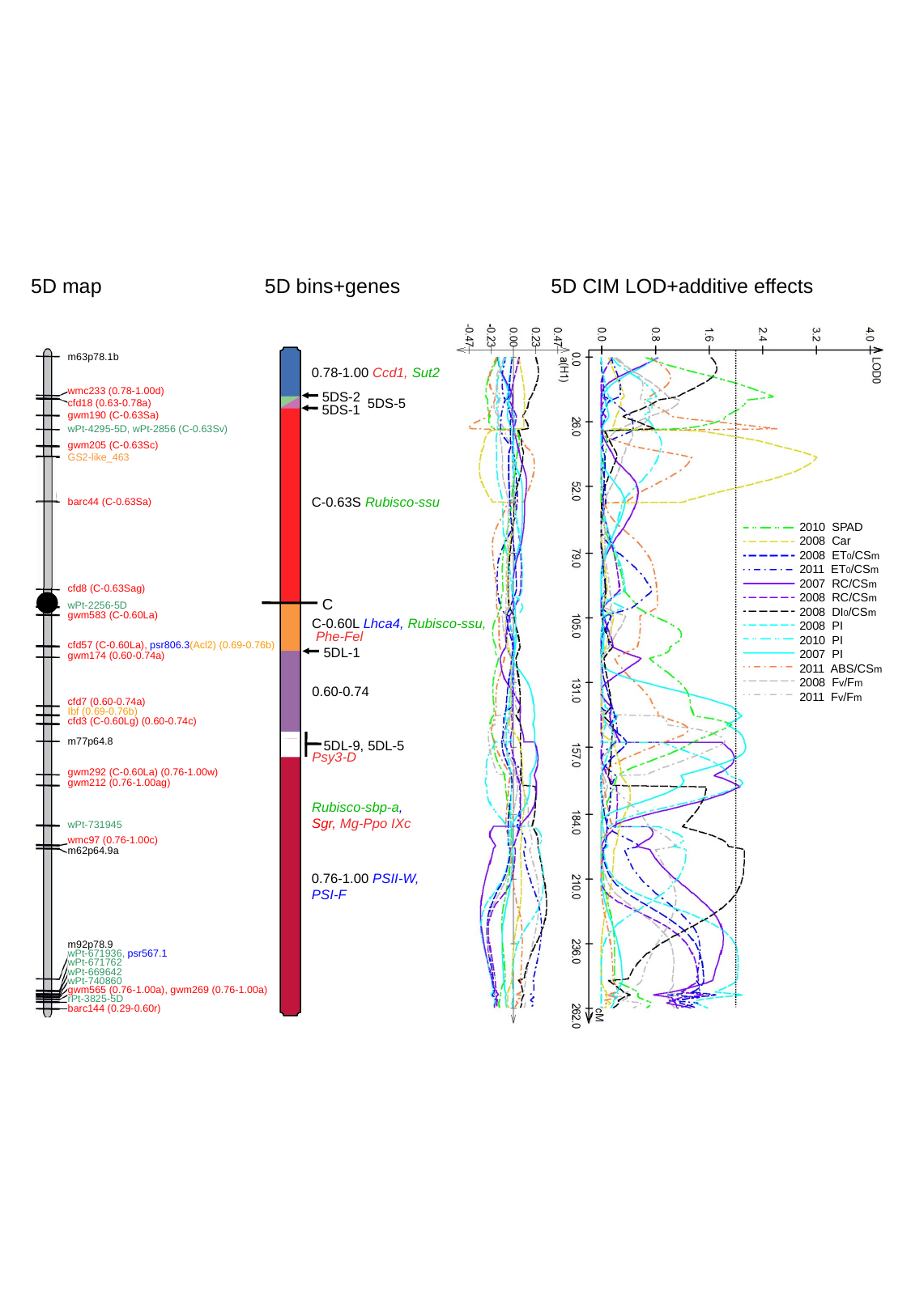

5D map
5D bins+genes
0.78-1.00 Ccd1, Sut2
5DS-2
5DS-5
5DS-1
C-0.63S Rubisco-ssu
C
C-0.60L Lhca4, Rubisco-ssu,
 Phe-Fel
5DL-1
0.60-0.74
5DL-9, 5DL-5
Psy3-D
Rubisco-sbp-a,
Sgr, Mg-Ppo IXc
0.76-1.00 PSII-W,
PSI-F
5D CIM LOD+additive effects
m63p78.1b
wmc233 (0.78-1.00d)
cfd18 (0.63-0.78a)
gwm190 (C-0.63Sa)
wPt-4295-5D, wPt-2856 (C-0.63Sv)
gwm205 (C-0.63Sc)
GS2-like_463
barc44 (C-0.63Sa)
cfd8 (C-0.63Sag)
wPt-2256-5D
gwm583 (C-0.60La)
cfd57 (C-0.60La), psr806.3(Acl2) (0.69-0.76b)
gwm174 (0.60-0.74a)
cfd7 (0.60-0.74a)
Ibf (0.69-0.76b)
cfd3 (C-0.60Lg) (0.60-0.74c)
m77p64.8
gwm292 (C-0.60La) (0.76-1.00w)
gwm212 (0.76-1.00ag)
wPt-731945
wmc97 (0.76-1.00c)
m62p64.9a
m92p78.9
wPt-671936, psr567.1
wPt-671762
wPt-669642
wPt-740860
gwm565 (0.76-1.00a), gwm269 (0.76-1.00a)
rPt-3825-5D
barc144 (0.29-0.60r)
2010 SPAD
2008 Car
2008 ET0/CSm
2011 ET0/CSm
2007 RC/CSm
2008 RC/CSm
2008 DI0/CSm
2008 PI
2010 PI
2007 PI
2011 ABS/CSm
2008 Fv/Fm
2011 Fv/Fm

## Slide 16
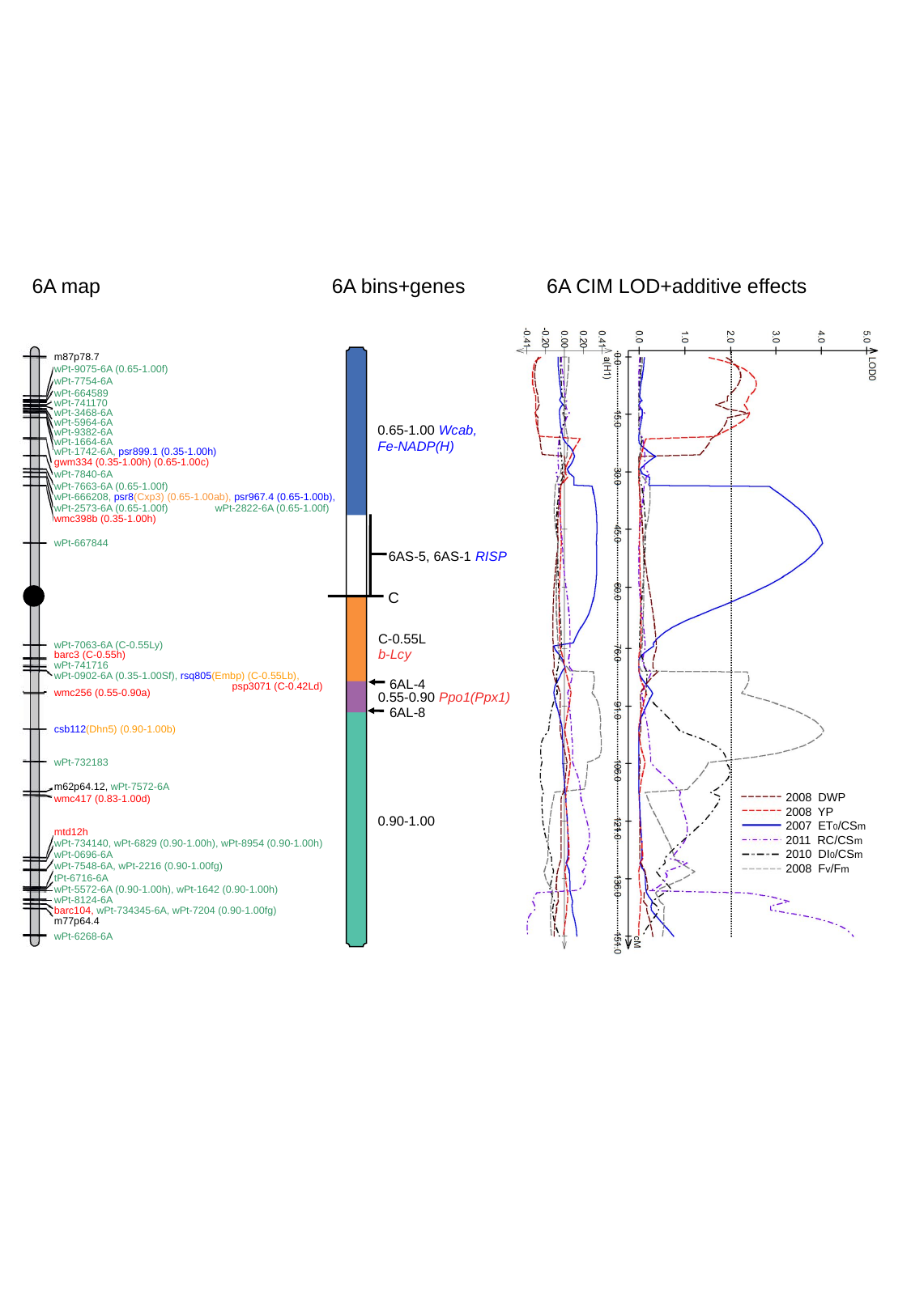

6A map
6A bins+genes
6A CIM LOD+additive effects
m87p78.7
wPt-9075-6A (0.65-1.00f)
wPt-7754-6A
wPt-664589
wPt-741170
wPt-3468-6A
wPt-5964-6A
wPt-9382-6A
wPt-1664-6A
wPt-1742-6A, psr899.1 (0.35-1.00h)
gwm334 (0.35-1.00h) (0.65-1.00c)
wPt-7840-6A
wPt-7663-6A (0.65-1.00f)
wPt-666208, psr8(Cxp3) (0.65-1.00ab), psr967.4 (0.65-1.00b),
wPt-2573-6A (0.65-1.00f)	 wPt-2822-6A (0.65-1.00f)
wmc398b (0.35-1.00h)
wPt-667844
wPt-7063-6A (C-0.55Ly)
barc3 (C-0.55h)
wPt-741716
wPt-0902-6A (0.35-1.00Sf), rsq805(Embp) (C-0.55Lb), 	 psp3071 (C-0.42Ld)
csb112(Dhn5) (0.90-1.00b)
wPt-732183
m62p64.12, wPt-7572-6A
wmc417 (0.83-1.00d)
mtd12h
wPt-734140, wPt-6829 (0.90-1.00h), wPt-8954 (0.90-1.00h)
wPt-0696-6A
wPt-7548-6A, wPt-2216 (0.90-1.00fg)
tPt-6716-6A
wPt-5572-6A (0.90-1.00h), wPt-1642 (0.90-1.00h)
wPt-8124-6A
barc104, wPt-734345-6A, wPt-7204 (0.90-1.00fg)
m77p64.4
wPt-6268-6A
0.65-1.00 Wcab,
Fe-NADP(H)
6AS-5, 6AS-1 RISP
C
C-0.55L
b-Lcy
6AL-4
wmc256 (0.55-0.90a)
0.55-0.90 Ppo1(Ppx1)
6AL-8
2008 DWP
2008 YP
2007 ET0/CSm
2011 RC/CSm
2010 DI0/CSm
2008 Fv/Fm
0.90-1.00

## Slide 17
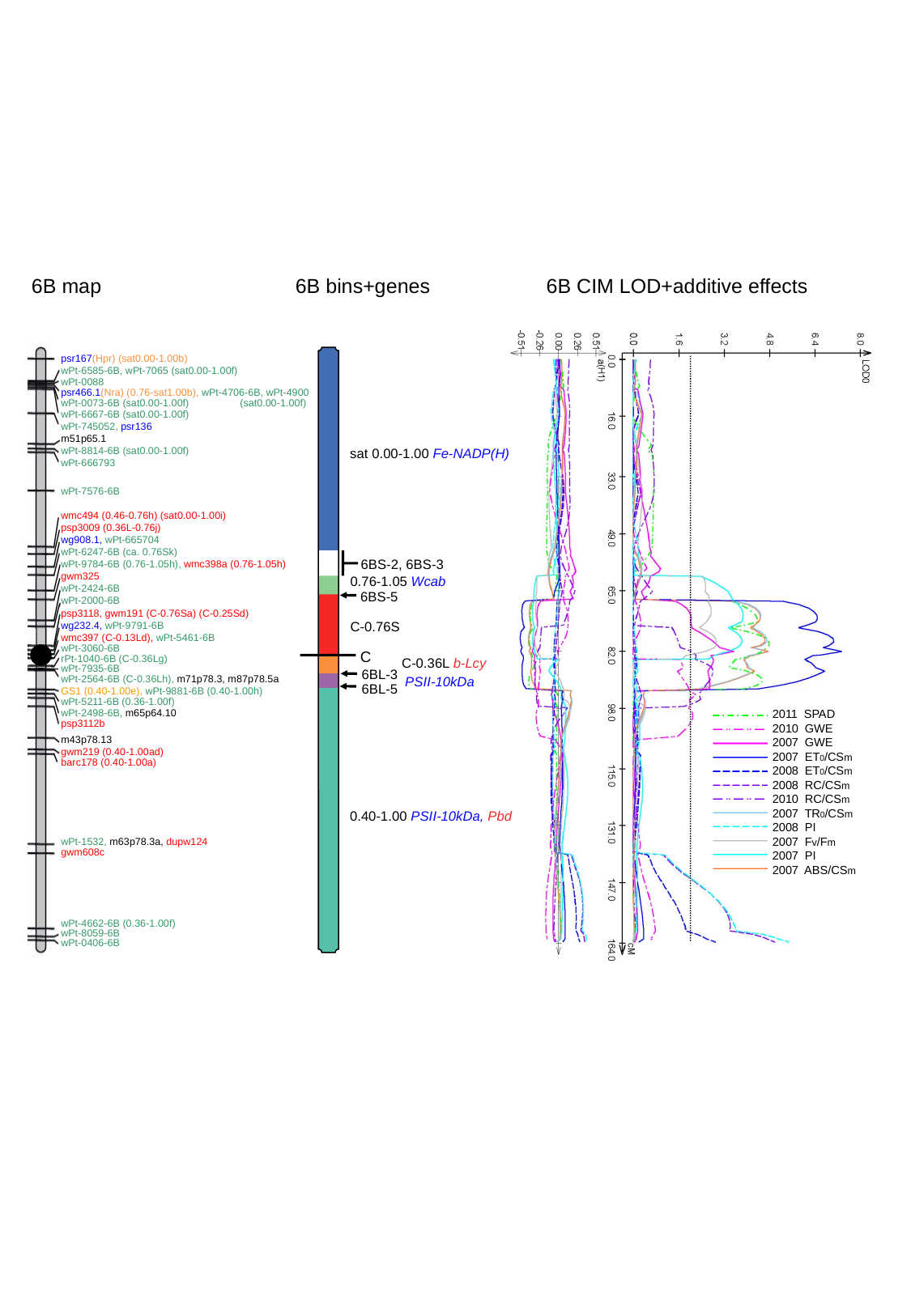

6B map
6B bins+genes
6B CIM LOD+additive effects
psr167(Hpr) (sat0.00-1.00b)
wPt-6585-6B, wPt-7065 (sat0.00-1.00f)
wPt-0088
psr466.1(Nra) (0.76-sat1.00b), wPt-4706-6B, wPt-4900
wPt-0073-6B (sat0.00-1.00f) (sat0.00-1.00f)
wPt-6667-6B (sat0.00-1.00f)
wPt-745052, psr136
m51p65.1
wPt-8814-6B (sat0.00-1.00f)
wPt-666793
wPt-7576-6B
wmc494 (0.46-0.76h) (sat0.00-1.00i)
psp3009 (0.36L-0.76j)
wg908.1, wPt-665704
wPt-6247-6B (ca. 0.76Sk)
wPt-9784-6B (0.76-1.05h), wmc398a (0.76-1.05h)
gwm325
wPt-2424-6B
wPt-2000-6B
psp3118, gwm191 (C-0.76Sa) (C-0.25Sd)
wg232.4, wPt-9791-6B
wmc397 (C-0.13Ld), wPt-5461-6B
wPt-3060-6B
rPt-1040-6B (C-0.36Lg)
wPt-7935-6B
wPt-2564-6B (C-0.36Lh), m71p78.3, m87p78.5a
GS1 (0.40-1.00e), wPt-9881-6B (0.40-1.00h)
wPt-5211-6B (0.36-1.00f)
wPt-2498-6B, m65p64.10
psp3112b
m43p78.13
gwm219 (0.40-1.00ad)
barc178 (0.40-1.00a)
wPt-1532, m63p78.3a, dupw124
gwm608c
wPt-4662-6B (0.36-1.00f)
wPt-8059-6B
wPt-0406-6B
sat 0.00-1.00 Fe-NADP(H)
6BS-2, 6BS-3
0.76-1.05 Wcab
6BS-5
C-0.76S
C
C-0.36L b-Lcy
6BL-3
PSII-10kDa
6BL-5
2011 SPAD
2010 GWE
2007 GWE
2007 ET0/CSm
2008 ET0/CSm
2008 RC/CSm
2010 RC/CSm
2007 TR0/CSm
2008 PI
2007 Fv/Fm
2007 PI
2007 ABS/CSm
0.40-1.00 PSII-10kDa, Pbd

## Slide 18
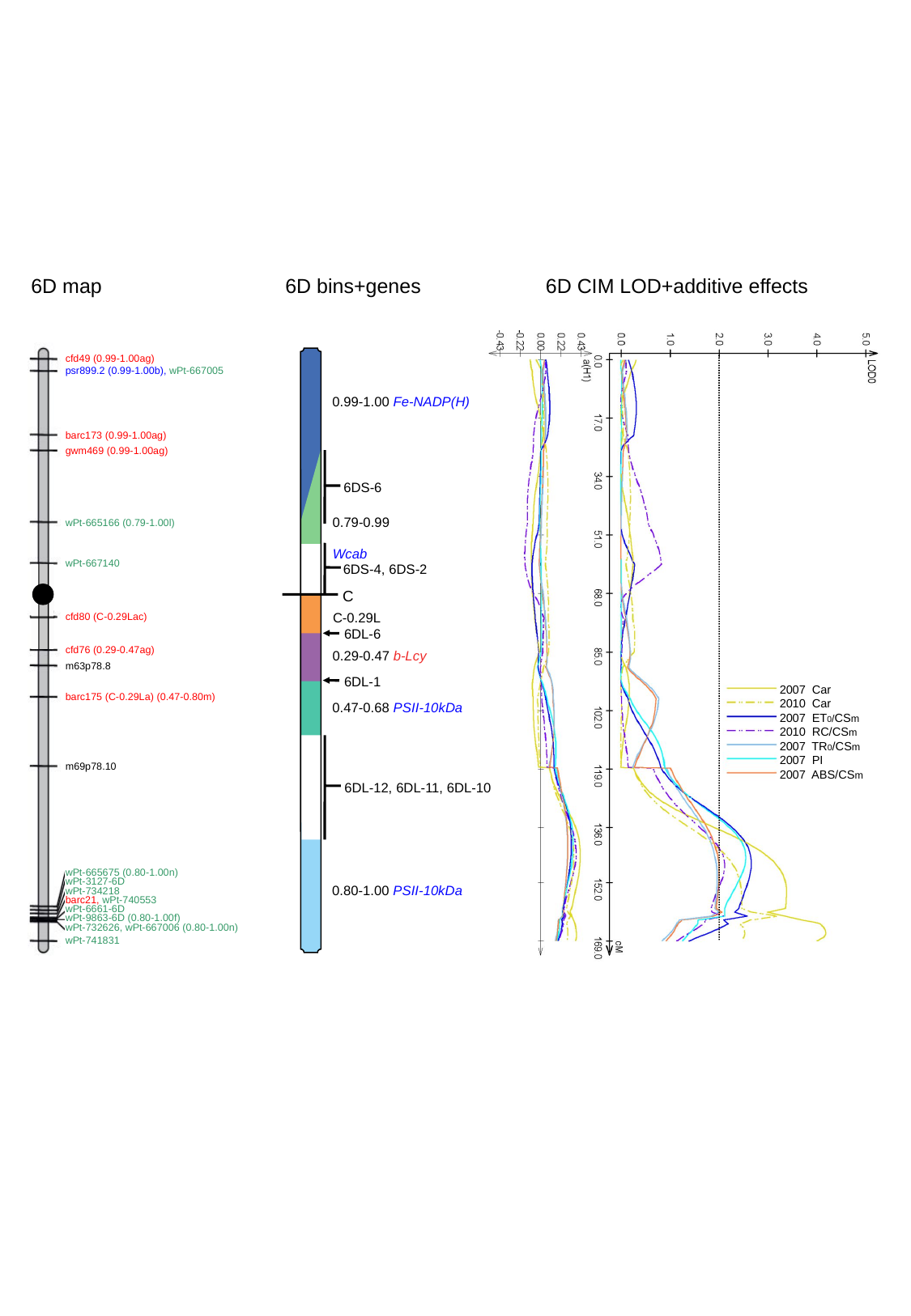

6D map
6D bins+genes
6D CIM LOD+additive effects
cfd49 (0.99-1.00ag)
psr899.2 (0.99-1.00b), wPt-667005
barc173 (0.99-1.00ag)
gwm469 (0.99-1.00ag)
wPt-665166 (0.79-1.00l)
wPt-667140
cfd80 (C-0.29Lac)
cfd76 (0.29-0.47ag)
m63p78.8
barc175 (C-0.29La) (0.47-0.80m)
m69p78.10
wPt-665675 (0.80-1.00n)
wPt-3127-6D
wPt-734218
barc21, wPt-740553
wPt-6661-6D
wPt-9863-6D (0.80-1.00f)
wPt-732626, wPt-667006 (0.80-1.00n)
wPt-741831
0.99-1.00 Fe-NADP(H)
6DS-6
0.79-0.99
Wcab
6DS-4, 6DS-2
C
C-0.29L
6DL-6
0.29-0.47 b-Lcy
6DL-1
2007 Car
2010 Car
2007 ET0/CSm
2010 RC/CSm
2007 TR0/CSm
2007 PI
2007 ABS/CSm
0.47-0.68 PSII-10kDa
6DL-12, 6DL-11, 6DL-10
0.80-1.00 PSII-10kDa

## Slide 19
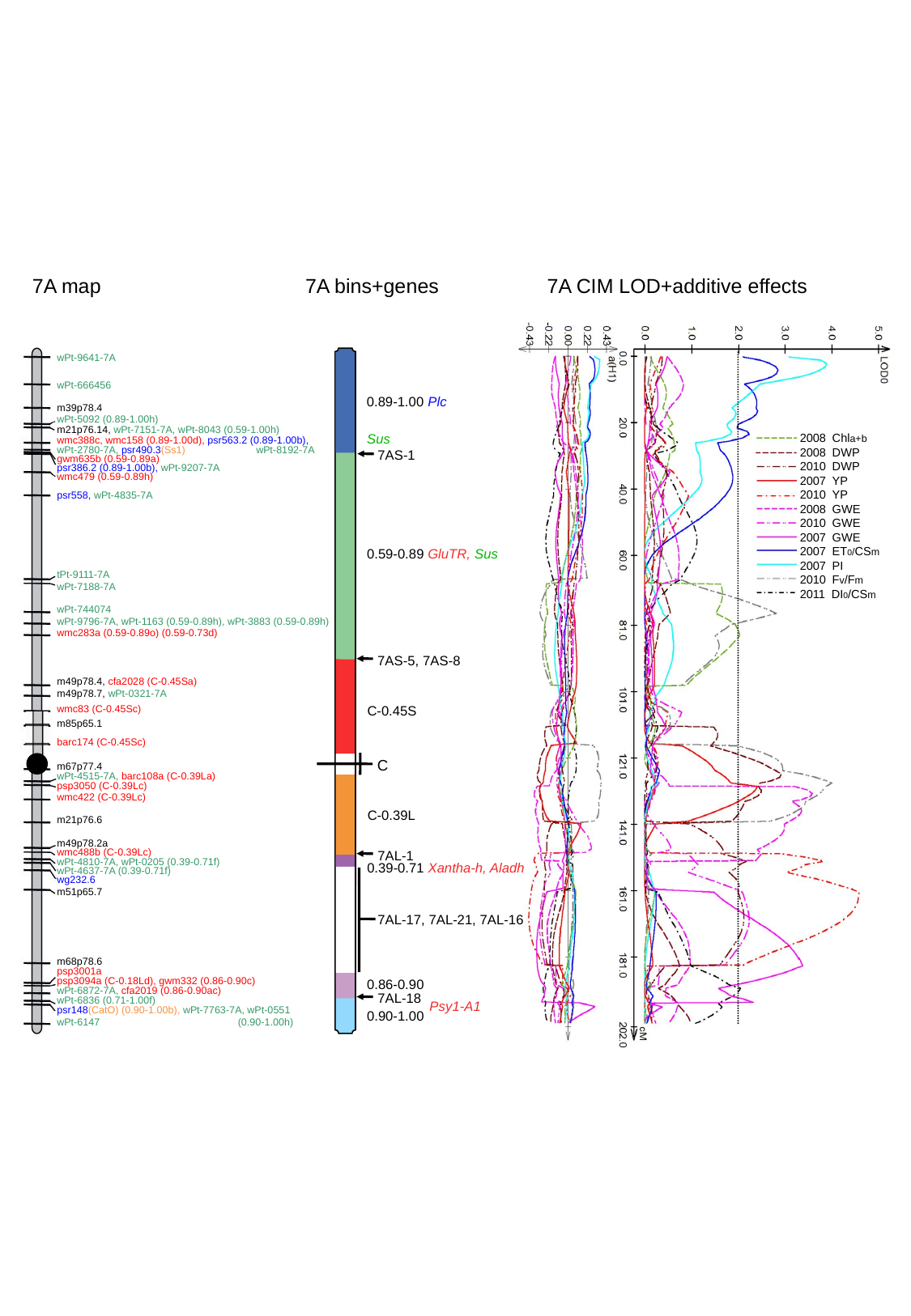

7A map
7A bins+genes
7A CIM LOD+additive effects
wPt-9641-7A
wPt-666456
m39p78.4
wPt-5092 (0.89-1.00h)
m21p76.14, wPt-7151-7A, wPt-8043 (0.59-1.00h)
wmc388c, wmc158 (0.89-1.00d), psr563.2 (0.89-1.00b),
wPt-2780-7A, psr490.3(Ss1) wPt-8192-7A
gwm635b (0.59-0.89a)
psr386.2 (0.89-1.00b), wPt-9207-7A
wmc479 (0.59-0.89h)
psr558, wPt-4835-7A
tPt-9111-7A
wPt-7188-7A
wPt-744074
wPt-9796-7A, wPt-1163 (0.59-0.89h), wPt-3883 (0.59-0.89h)
wmc283a (0.59-0.89o) (0.59-0.73d)
m49p78.4, cfa2028 (C-0.45Sa)
m49p78.7, wPt-0321-7A
wmc83 (C-0.45Sc)
m85p65.1
barc174 (C-0.45Sc)
m67p77.4
wPt-4515-7A, barc108a (C-0.39La)
psp3050 (C-0.39Lc)
wmc422 (C-0.39Lc)
m21p76.6
m49p78.2a
wmc488b (C-0.39Lc)
wPt-4810-7A, wPt-0205 (0.39-0.71f)
wPt-4637-7A (0.39-0.71f)
wg232.6
m51p65.7
m68p78.6
psp3001a
psp3094a (C-0.18Ld), gwm332 (0.86-0.90c)
wPt-6872-7A, cfa2019 (0.86-0.90ac)
wPt-6836 (0.71-1.00f)
psr148(CatO) (0.90-1.00b), wPt-7763-7A, wPt-0551
wPt-6147	 (0.90-1.00h)
0.89-1.00 Plc
Sus
2008 Chla+b
2008 DWP
2010 DWP
2007 YP
2010 YP
2008 GWE
2010 GWE
2007 GWE
2007 ET0/CSm
2007 PI
2010 Fv/Fm
2011 DI0/CSm
7AS-1
0.59-0.89 GluTR, Sus
7AS-5, 7AS-8
C-0.45S
C
C-0.39L
7AL-1
0.39-0.71 Xantha-h, Aladh
7AL-17, 7AL-21, 7AL-16
0.86-0.90
7AL-18
Psy1-A1
0.90-1.00

## Slide 20
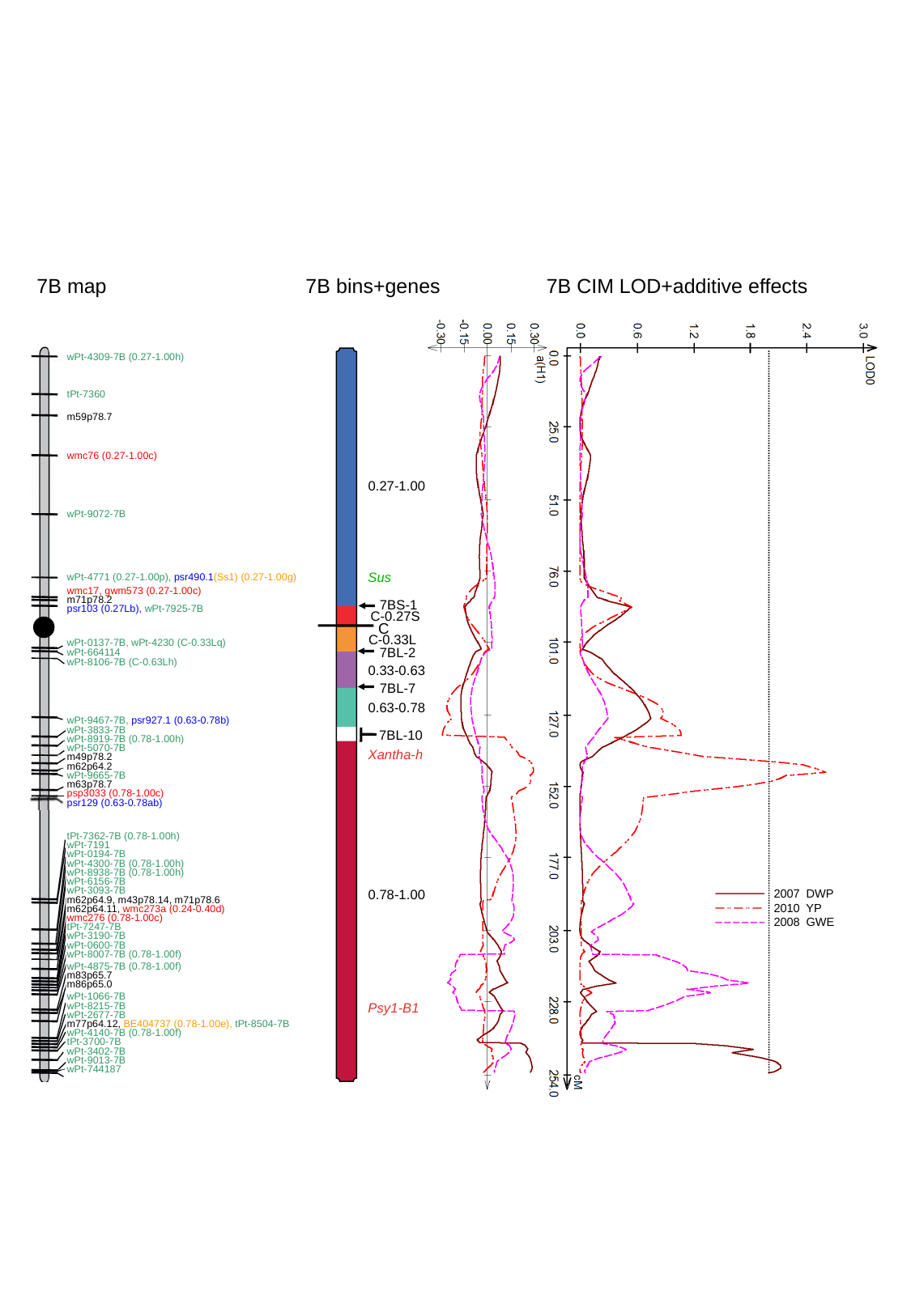

7B map
7B bins+genes
7B CIM LOD+additive effects
wPt-4309-7B (0.27-1.00h)
tPt-7360
m59p78.7
wmc76 (0.27-1.00c)
wPt-9072-7B
wPt-4771 (0.27-1.00p), psr490.1(Ss1) (0.27-1.00g)
wmc17, gwm573 (0.27-1.00c)
m71p78.2
psr103 (0.27Lb), wPt-7925-7B
wPt-0137-7B, wPt-4230 (C-0.33Lq)
wPt-664114
wPt-8106-7B (C-0.63Lh)
wPt-9467-7B, psr927.1 (0.63-0.78b)
wPt-3833-7B
wPt-8919-7B (0.78-1.00h)
wPt-5070-7B
m49p78.2
m62p64.2
wPt-9665-7B
m63p78.7
psp3033 (0.78-1.00c)
psr129 (0.63-0.78ab)
tPt-7362-7B (0.78-1.00h)
wPt-7191
wPt-0194-7B
wPt-4300-7B (0.78-1.00h)
wPt-8938-7B (0.78-1.00h)
wPt-6156-7B
wPt-3093-7B
m62p64.9, m43p78.14, m71p78.6
m62p64.11, wmc273a (0.24-0.40d)
wmc276 (0.78-1.00c)
tPt-7247-7B
wPt-3190-7B
wPt-0600-7B
wPt-8007-7B (0.78-1.00f)
wPt-4875-7B (0.78-1.00f)
m83p65.7
m86p65.0
wPt-1066-7B
wPt-8215-7B
wPt-2677-7B
m77p64.12, BE404737 (0.78-1.00e), tPt-8504-7B
wPt-4140-7B (0.78-1.00f)
tPt-3700-7B
wPt-3402-7B
wPt-9013-7B
wPt-744187
0.27-1.00
Sus
7BS-1
C-0.27S
C
C-0.33L
7BL-2
0.33-0.63
7BL-7
0.63-0.78
7BL-10
Xantha-h
0.78-1.00
2007 DWP
2010 YP
2008 GWE
Psy1-B1

## Slide 21
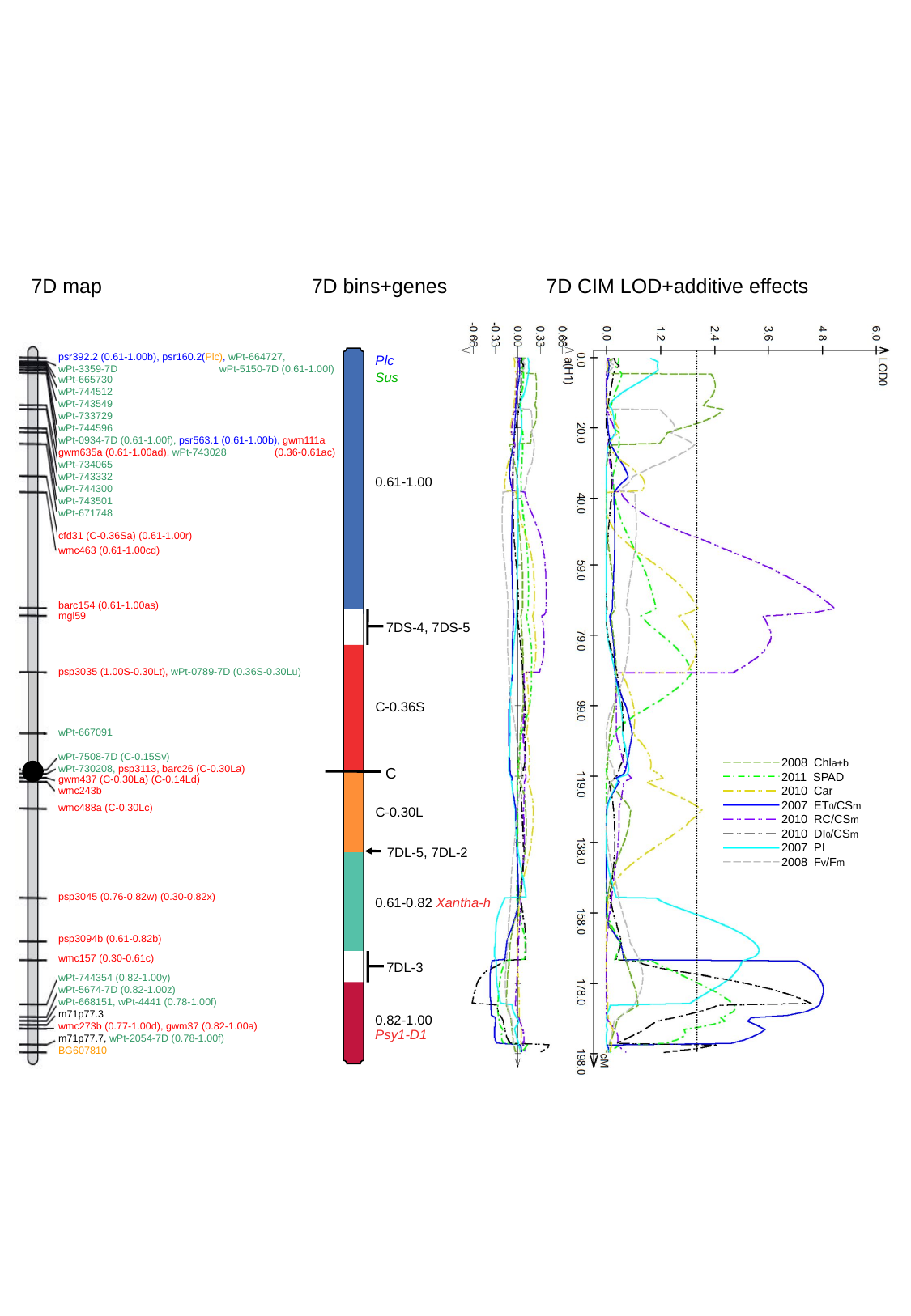

7D map
7D bins+genes
7D CIM LOD+additive effects
psr392.2 (0.61-1.00b), psr160.2(Plc), wPt-664727,
wPt-3359-7D	 wPt-5150-7D (0.61-1.00f)
wPt-665730
wPt-744512
wPt-743549
wPt-733729
wPt-744596
wPt-0934-7D (0.61-1.00f), psr563.1 (0.61-1.00b), gwm111a
gwm635a (0.61-1.00ad), wPt-743028 (0.36-0.61ac)
wPt-734065
wPt-743332
wPt-744300
wPt-743501
wPt-671748
cfd31 (C-0.36Sa) (0.61-1.00r)
wmc463 (0.61-1.00cd)
barc154 (0.61-1.00as)
mgl59
psp3035 (1.00S-0.30Lt), wPt-0789-7D (0.36S-0.30Lu)
wPt-667091
wPt-7508-7D (C-0.15Sv)
wPt-730208, psp3113, barc26 (C-0.30La)
gwm437 (C-0.30La) (C-0.14Ld)
wmc243b
wmc488a (C-0.30Lc)
psp3045 (0.76-0.82w) (0.30-0.82x)
psp3094b (0.61-0.82b)
wmc157 (0.30-0.61c)
wPt-744354 (0.82-1.00y)
wPt-5674-7D (0.82-1.00z)
wPt-668151, wPt-4441 (0.78-1.00f)
m71p77.3
wmc273b (0.77-1.00d), gwm37 (0.82-1.00a)
m71p77.7, wPt-2054-7D (0.78-1.00f)
BG607810
Plc
Sus
0.61-1.00
7DS-4, 7DS-5
C-0.36S
2008 Chla+b
2011 SPAD
2010 Car
2007 ET0/CSm
2010 RC/CSm
2010 DI0/CSm
2007 PI
2008 Fv/Fm
C
C-0.30L
7DL-5, 7DL-2
0.61-0.82 Xantha-h
7DL-3
0.82-1.00
Psy1-D1
